# Conformation of the Ester Group Governs the Photophysics of Highly Polarized Benzo[*g*]coumarins

**DOI:** 10.1021/jacsau.3c00169

**Published:** 2023-06-26

**Authors:** Kamil Szychta, Beata Koszarna, Marzena Banasiewicz, Andrzej Sobolewski, Omar O’Mari, John A. Clark, Valentine I. Vullev, Cristina A. Barboza, Daniel T. Gryko

**Affiliations:** † Institute of Organic Chemistry of Polish Academy of Sciences, Kasprzaka 44/52, 01-224 Warsaw, Poland; ‡ Institute of Physics of Polish Academy of Sciences, Al. Lotników 32/46, 02-668 Warsaw, Poland; § Department of Physical and Quantum Chemistry, Faculty of Chemistry, Wroclaw University of Science and Technology, Wrocław 50-370, Poland; ∥ Department of Bioengineering, University of California, Riverside, California 92521, United States; ⊥ Department of Chemistry, Department of Biochemistry, and Materials Science and Engineering Program, University of California, Riverside, California 92521, United States

**Keywords:** charge transfer, benzo[g]coumarins, dual emission, coumarins, twisted intramolecular charge-transfer state

## Abstract

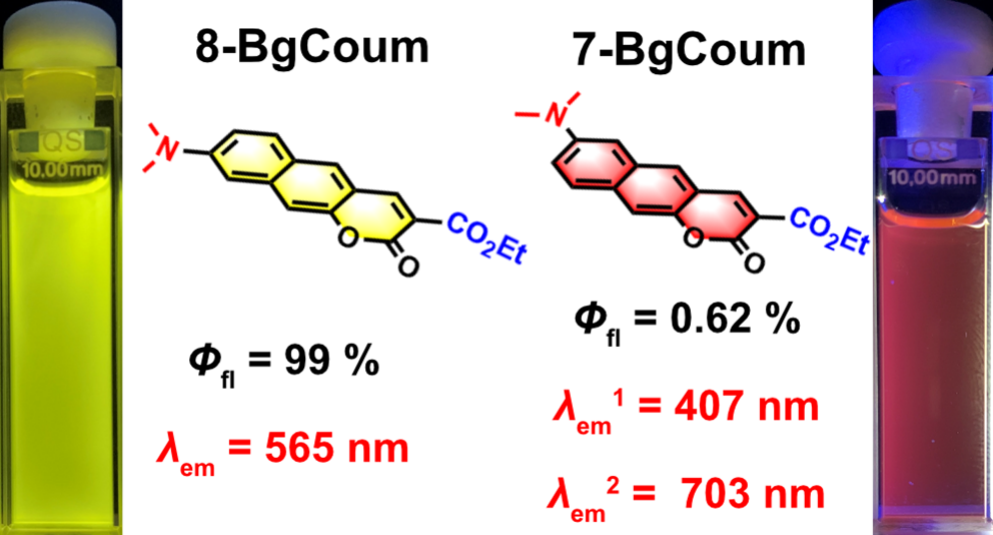

Photosensitizers that display “unusual” emission from upper electronically excited states offer possibilities for initiating higher-energy processes than what the governing Kasha’s rule postulates. Achieving conditions for dual fluorescence from multiple states of the same species requires molecular design and conditions that favorably tune the excited-state dynamics. Herein, we switch the position of the electron-donating NMe_2_ group around the core of benzo[*g*]coumarins (BgCoum) and tune the electronic coupling and the charge-transfer character of the fluorescent excited states. For solvents with intermediate polarity, three of the four regioisomers exhibit fluorescence from two different excited states with bands that are well separated in the visible and the near-infrared spectral regions. Computational analysis, employing ab initio methods, reveals that the orientation of an ester on the pyrone ring produces two conformers responsible for the observed dual fluorescence. Studies with solid solvating media, which restricts the conformational degrees of freedom, concur with the computational findings. These results demonstrate how “seemingly inconsequential” auxiliary substituents, such as the esters on the pyrone coumarin rings, can have profound effects leading to “anti-Kasha” photophysical behavior important for molecular photonics, materials engineering, and solar-energy science.

## Introduction

For decades, Kasha’s rule, along with its subsequent Vavilov’s rule, have served as fundamental guidelines in molecular photonics.^1^ They are corollary of the inherently fast internal conversion (IC) between electronically excited states making the deactivation of the lowest excited states with the same multiplicity the rate-limiting step.^2^ This photophysical behavior prevents harvesting the full scale of optical excitation energy and accessing reaction pathways originating from upper excited states.^3−5^ Therefore, systems that do not follow Kasha’s rule and show, for example, two fluorescence bands from excited state with different energies, i.e., dual fluorescence, present important paradigms for areas such as solar-energy science, optical imaging, and biosensing.^6−9^

Azulene is the “poster child” of chromophores that violate Kasha’s rule.^10^ The rate of radiative deactivation of its S_2_ state is comparable to that of S_2_ → S_1_ IC, resulting in strong S_2_ → S_0_ fluorescence.^11,12^ Similarly, other chromophores, such as ketocyanine dyes and sub-porphyrins can exhibit fluorescence from their S_2_ and S_3_ states.^13,14^

Modulating the rates of excited-state charge transfer (CT), intersystem crossing (ISC) and proton-transfer (PT)-mediated tautomerization can also produce two emissive states with different energies that undergo radiative decay with equal efficacy. Chromophores with emissive (CT) and locally excited (LE) states display dual fluorescence when the rates of CT are comparable to the rates of deactivation of the LE state.^15,16^ In solids, ISC from upper states can provide a means for populating states with different multiplicity and attaining dual emission encompassing fluorescence and phosphorescence bands.^17,18^ Excited-state PT producing multiple tautomers can similarly lead to dual fluorescence from the populations with different energy gaps between their emissive and ground states.^19^ Tautomerization of free-base corroles allows picosecond access to higher energies than that of their lowest excited states. Overall, excited-state isomerization that populates two local minima of emissive structures with different energies above the Franck–Condon (FC) ground states provides a plausible means for attaining dual-fluorescence behavior.^20^

In the context of excited-state CT and conformational dynamics that may lead to dual fluorescence, we turn our attention to aminocoumarins. Developed as strongly fluorescent and photostable laser dyes, they have become popular photoprobes for biomedical imaging.^21,22^ The amine and the electron-deficient pyrone ring form a donor–acceptor pair efficiently mediating ICT in the excited state and producing broad CT fluorescence with a huge Stokes’ shift.^23−25^ Although studied for more than a century, coumarins and π-expanded coumarins are well-known to produce emission from their low-lying CT states with no traces of dual fluorescence from upper states.

Coumarin was first isolated in 1820 from the beans of *Dipteryx odorata*, which gave its name.^26^ The first π-expanded coumarin was synthesized by von Pechmann in 1887.^27^ Nevertheless, this field has only catapulted to prominence over the last decade. In this short time period, multiple classes of synthesized and characterized π-expanded coumarins, including conjoined and helical coumarins, have emerged.^28−38^

The fluorescence of 7-aminocoumarins is one of the most intensively studied and puzzling topics in the field of photophysics of organic chromophores.^39^ Important early contributions by Jones^40−42^ revealed that substituents on and in the proximity of the amino-nitrogen atom strongly affect their emission characteristics. These reports were followed by breakthrough analysis by numerous authors^43−46^ with critical contributions coming from Rettig^47^ and Cole.^48^ Recently two structural motifs that retain reasonably high fluorescence quantum yields of 7-aminocoumarins in very polar solvents emerged.^49−51^ At the same time the work by Cole, Maciejewski, and others revealed that shifting the position of the amino group to position 6 has a profound effect on the photophysics, typically enlarging the Stokes’ shift and decreasing the fluorescence quantum yield.^25,52−55^

**Figure 1:**
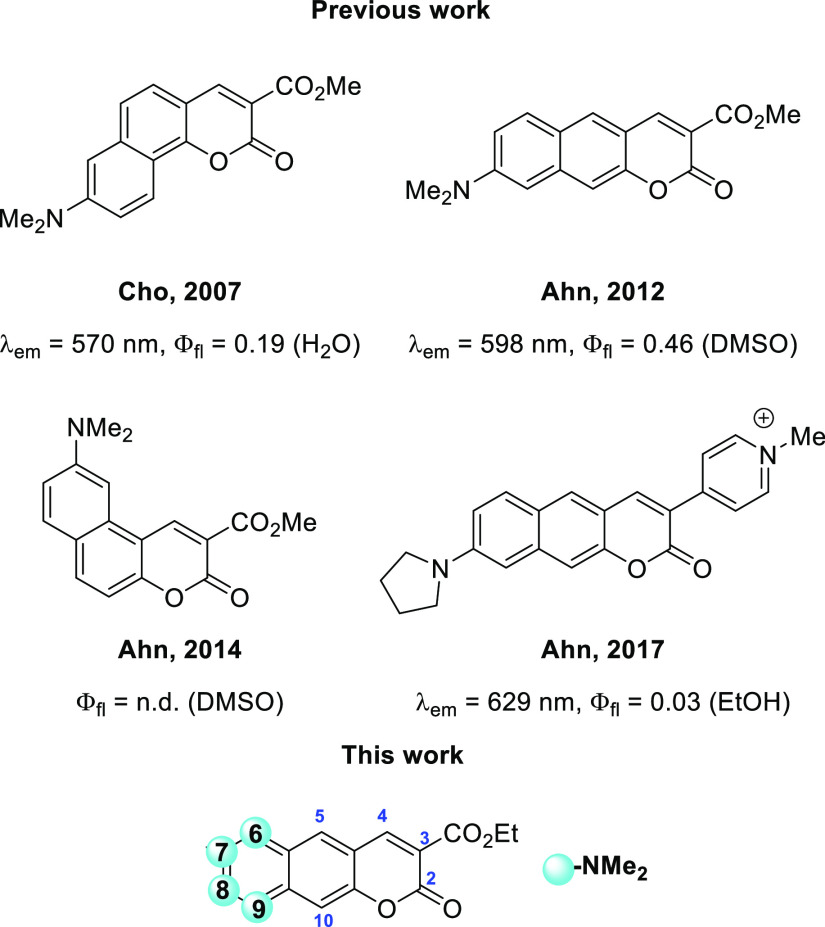
State-of-the art in strongly polarized benzocoumarins.

The first example of benzocoumarin possessing NMe_2_ group was published by Cho and co-workers in 2007.^56^ This benzo[*h*]coumarin (Figure 1) displayed remarkably strong fluorescence in water and moderate bathochromic shift of emission. In the breakthrough discovery, Ahn was the first to recognize that linear expansion of coumarin into benzo[*g*]coumarin while maintaining the presence of the dialkylamino group (Figure 1) leads to superb photophysical properties (i.e., large fluorescence quantum yields, reasonable Stokes’ shift, and so forth) accompanied by a redshift of the emission band to ca. 600 nm.^57^ Indeed, the methyl ester of 8-dimethylaminobenzo[*g*]coumarin has very promising properties from the point of view of bioimaging, featuring strong emission even in polar solvents.^58^ The subsequent systematic study reveals that further modification toward benzo[*f*]coumarin series has a detrimental effect on emission intensity.^59^ The 8-dialkylaminobenzo[*g*]coumarin motive has been subsequently explored by Ahn and co-workers in numerous contributions addressing, e.g., autofluorescence in deep tissue imaging^58,60^ and discrepancy of fluorescence properties in solutions and in cells.^61^ The discovery of 8-dimethylamino-benzo[*g*]coumarin led to numerous papers exploring this dye as next generation fluorescent probes.^61−64^

Considering this wealth of knowledge, the question at hand is, can altering the position of the amine in π-expanded coumarins tune its electronic coupling with the aromatic core in order to attain dual fluorescence from different electronic states?

Herein, we report a discovery of dual fluorescence from benzo[*g*]coumarin (**BgCoum**) derivatives (Figure 1). While methyl 8-dimethylaminobenzo[*g*]coumarin-3-carboxylate (**8-BgCoum**) shows a single broad fluorescence band exhibiting pronounced solvatochromism and a large Stokes’ shift consistent with radiative decay of a CT state,^65^ moving the amine substituent to positions 6, 7, and 9 induces the emergence of short-wavelength emission for solvents with intermediate polarity. As important as the amine position is, to our surprise, it is the rotation of an ester substituent on the pyrone ring that defines the emergence of the dual fluorescence, as ab initio computational studies reveal. This unexpected paradigm of photoinduced dynamics makes π-expanded coumarins promising candidates for exploring initiation of intermolecular processes from upper excited states.

## Results and Discussion

### Molecular Design and Synthesis

Introducing a dimethylamino group (NMe_2_) at positions **6**, **7**, **8**, and **9** of a benzo[*g*]coumarin scaffold allows examining the effects of the position of strong electron-donating substituents on the photophysics of this class of π-expanded dyes. Such alterations of the position of NMe_2_ varies its electronic coupling with the coumarin core and, especially, with the electron-deficient pyrone ring modified with an ethoxycarbonyl group (CO_2_Et). As an electron withdrawing group with Hammett constants of 0.37 and 0.45, CO_2_Et enhances the propensity of the pyrone ring to act as an electron acceptor.^66^ Nevertheless, while the Swain–Lupton (SL) field constant of CO_2_Et is 0.34, consistent with its electron-withdrawing property, the SW resonance constant of ethoxycarbonyl is only 0.11,^67^ suggesting relatively weak mesomeric effects and perturbation of the coumarin electronic transitions involving frontier orbitals with a π-character.

**Scheme 1:**
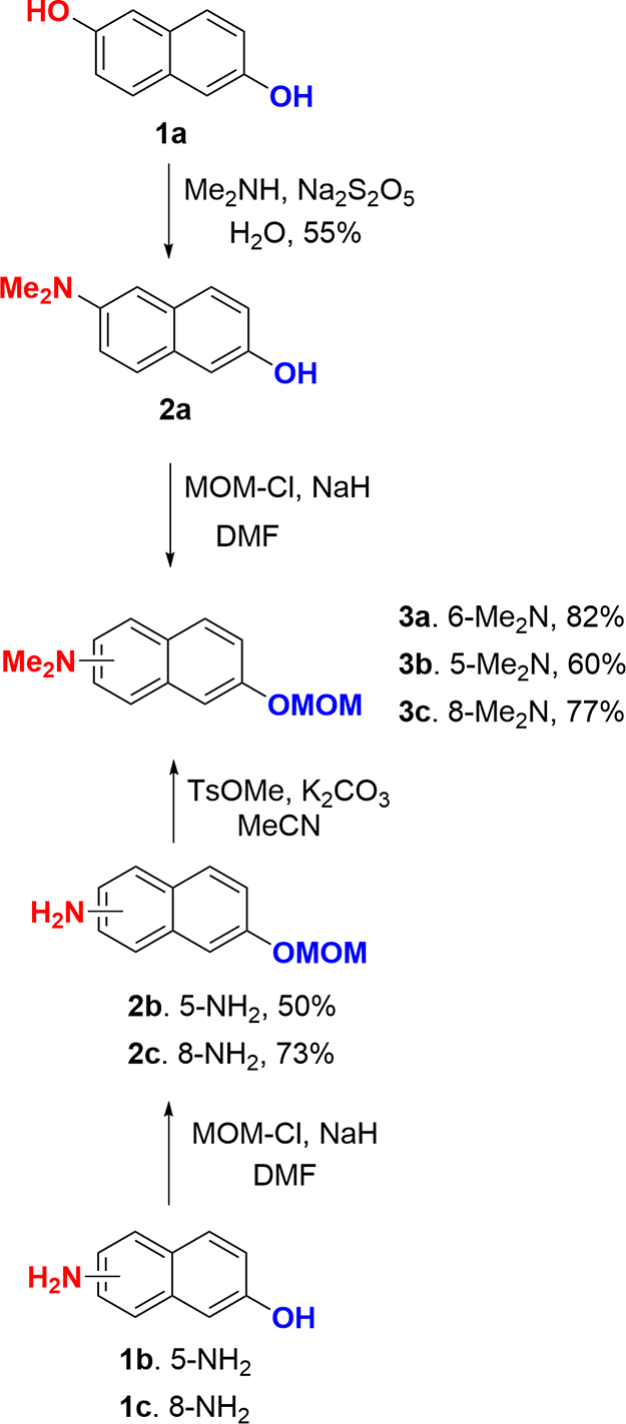
Synthesis of Compounds **3a–c**

In addition to the **8-BgCoum** derivative that we have already reported,^65^ we synthesize the new 6, 7, and 9 regioisomeric benzo[*g*]coumarins in four steps using a method analogous to that described in the literature.^58^ Monosubstitution of 2,6-dihydroxynaphthalene with dimethylamine under Bucherer reaction conditions yields compound **2a** (Scheme 1). For coumarins **6-BgCoum** and **9-BgCoum**, we use the commercially available 5-amino-2-hydroxynaphthalene and 8-amino-2-hydroxynaphthalene as substrates (Scheme 1). Employing chloromethyl methyl ether (MOM-Cl) allows protecting the hydroxyl group to afford compounds **2b** and **2c**. Using the same method to protect the hydroxyl group of **2a**, it is converted into the methoxymethyl ether **3a**. Methylation of the amino group using methyl *p*-toluenesulfonate leads to compounds **3b** and **3c** with an overall efficiency of 50%. The use of other methylation reagents gives significantly lower yields.

**Scheme 2:**
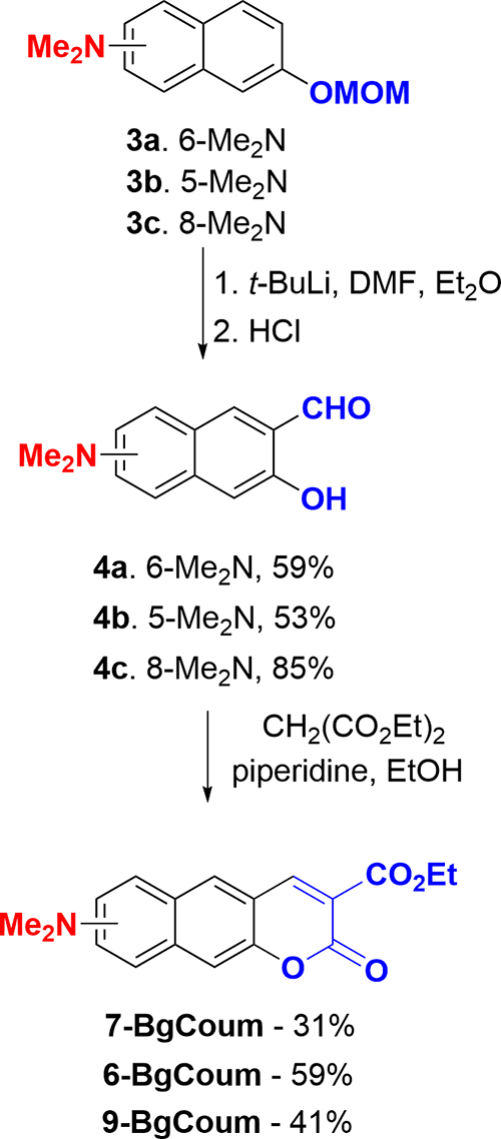
Synthesis of Dyes **6-BgCoum**, **7-BgCoum**, and **9-BgCoum** from the Compounds **3a–c**

Ortho-formylation relative to the protected hydroxyl group of the dimethylaminonaphthalene derivatives leads to the next key intermediates **4a–c**. Specifically, introducing methoxymethyl to the hydroxyl group allowed for the Snieckus-type lithiation of **3** (Scheme 2). Treating these lithioorganic derivatives with *N*,*N*-dimethylformamide (DMF) as a formylation agent, and afterward with an aqueous solution of HCl, yields the aldehydes **4a–c** directly, without the need to separately deprotect the hydroxyl group. The Knoevenagel condensation of diethyl malonate with **4** results in the formation of compounds **6-BgCoum**, **7-BgCoum**, and **9-BgCoum** in yields of 59, 31, and 41%, respectively (Scheme 2).

### Photophysical Behavior: Unusual Dual Fluorescence

**Figure 2:**
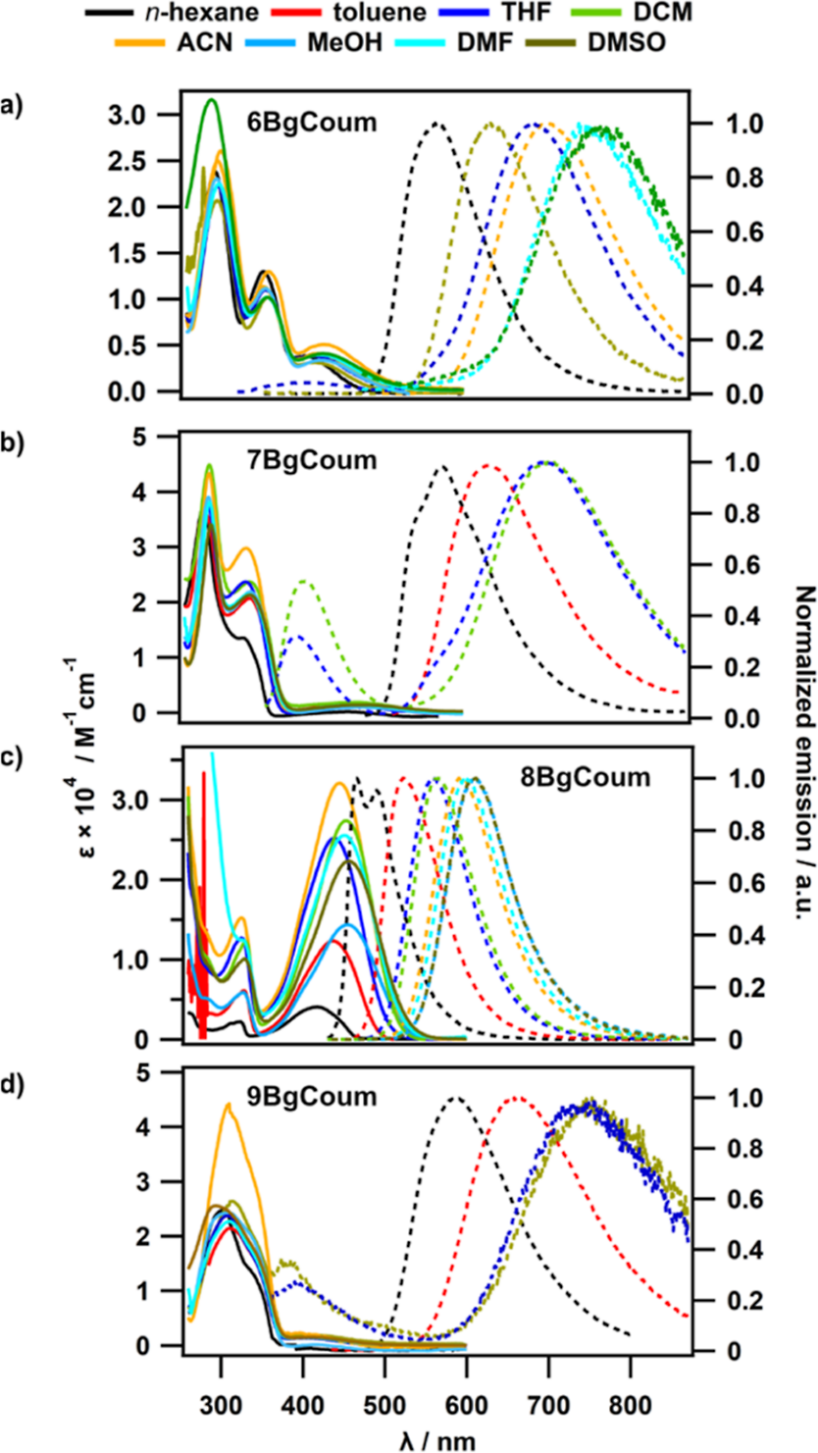
Absorption (solid lines) and emission (dashed lines) spectra of (a) **6-BgCoum** (excited at 345 nm), (b) **7-BgCoum** (excited at 330 nm), (c) **8-BgCoum** (excited at 340 nm),^65^ and (d) **9-BgCoum** (excited at 317 nm).

All four **BgCoum** NMe_2_ derivatives show a broad fluorescence band that manifests solvatochromic behavior (Figure 2). In addition to the bathochromic shifts of the emission, an increase in solvent polarity induces a decrease in the fluorescence quantum yields (Φ_f_) of the **BgCoum** compounds (Table 1). The origin of the observed broad fluorescence band, therefore, is consistent with radiative deactivation of an excited state with a pronounced CT character, i.e., S_1_^(CT)^ → S_0_. That is, an increase in solvent polarity stabilizes the emissive CT states of the aminocoumarins, bringing them closer to the conical intersection with the S_0_ state and drastically increasing the non-radiative-decay rate constants, *k*_nr_ (Table 1).

**Table 1:** Photophysical Data of **6-BgCoum**, **7-BgCoum**, **8-BgCoum**, and **9-BgCoum** Measured in Solution and in Crystalline State

solvent^a^	Φ_f_ (Φ_f_ ^SW^, Φ_f_ ^LW^) [%]	τ [ns]	k_r_·10^–7^ [s^–1^]	k_nr_·10^–7^ [s^–1^]
**6-BgCoum**				
*n*-hexane	50.5	13.4	3.76	3.69
toluene	26.1	6.0	4.33	12.3
DCM	3.5	0.9	12.2	96.7
THF	5.9 (0.2, 11.2)	1.0	3.35	91.9
ACN	0.6	0.2	2.94	465.0
DMSO	0.7	0.19	3.67	519
DMF	0.7	0.24	2.92	415
propyl butyrate	0.1 (0.02, 0.91)	2.4	0.38	41.3
SOA	5.6 (0.21, 5.8)	7.16	0.81	13.2
**7-BgCoum**				
*n*-hexane	14.4	13.1	1.10	6.54
toluene	8.9	9.9	0.82	9.27
DCM	0.62 (0.1, 3.9)	1.5	1.38	63.3
THF	0.95 (0.02, 2.1)	3.9	0.99	24.7
DMSO				
DMF				
propyl butyrate	0.54 (0.2, 0.13)	5.27	0.02	19.0
SOA	2.1 (0.39, 0.61)	12.8	0.05	7.8
**8-BgCoum**				
*n*-hexane	11.0	0.76	14.0	118.0
toluene	98.0	4.2	23.0	0.3
DCM	99.9	4.65	21.5	0.02
THF	95.0	4.76	20.0	1.1
ACN	86.0	4.8	17.0	2.9
DMSO	73.2	4.18	17.5	6.4
DMF	72.9	4.28	17.0	6.33
propyl butyrate	34.0	4.38	7.8	15.1
SOA	49.2	4.24	11.6	12.0
**9-BgCoum**				
*n*-hexane	8.3	6.3	1.31	14.5
toluene	0.90	1.6	0.55	62.6
DCM	0.09 (0.003, 0.11)	2.8	0.27	35.0
THF	0.08 (0.006, 0.09)	2.6	0.031	38.22
DMSO				
DMF				
propyl butyrate	0.03 (0.01, 0.01)	0.70	0.02	142.8
SOA	0.80 (0.12, 1.2)	5.85	0.21	16.9

a Onsager polarity, *f*_O_(ε, *n*^2^), of the used solvents: hexane—0.00, toluene—0.03, sucrose octaacetate (SOA)—0.27, propyl butyrate—0.30, tetrahydrofuran (THF)—0.41, dichloromethane (DCM)—0.43, acetonitrile (ACN)—0.46, dimethyl sulfoxide (DMSO)—0.53, *N*,*N*-dimethylformamide (DMF)—0.55. *f*_O_(ε, *n*^2^) = *f*_O_(ε) – *f*_O_(*n*^2^), where *f*_O_(*x*) = 2(*x* – 1) (2*x* + 1)^−1^.

The absorption spectrum of **8-BgCoum** shows a large-amplitude band around 420–450 nm, consistent with a S_0_ → S_1_^(CT)^ transition, along with a weak ultraviolet (UV) signal originating from excitation to upper singlet state (Figure 2c). The solvatochromic response of the S_0_ → S_1_^(CT)^ absorption, however, is not as pronounced as that of the S_1_^(CT)^ → S_0_ emission, and the Stokes’ shift (Δν̃) increases with an increase in solvent polarity (Table 1). Enhancing the charge separation (CS), therefore, appears to follow the initial photoexcitation to the Franck–Condon (FC) CT state.

The UV transitions dominate the absorption spectra of **6-BgCoum**, **7-BgCoum**, and **9-BgCoum**, and they do not show as strong CT bands in the 400 nm region as **8-BgCoum** (Figure 2). The overlap between the natural transition orbitals (NTOs) for the S_0_ → S_1_^(CT)^ excitation is, thus, smaller in **6-BgCoum** than in **8-BgCoum**, and it appears especially minute in **7-BgCoum** and **9-BgCoum**.

Most importantly, ultraviolet (UV) excitation of **6-BgCoum**, **7-BgCoum**, and **9-BgCoum** in solvent with intermediate polarity, such as dichloromethane (DCM), tetrahydrofuran (THF), and propyl butyrate (PrBu), leads to the emergence of a new additional fluorescence band around 400 nm that is hypsochromically shifted in relevance to the CT absorption (Figure 2). In the emission spectra of the weakly fluorescent **7-BgCoum** and **9-BgCoum**, the amplitude of this newly emerged short-wavelength band is only about two-to-four times smaller than that of the broad CT band. Conversely, the 400 nm emission of **6-BgCoum** in THF is barely visible on the background of the strong CT fluorescence. For **8-BgCoum**, which generally exhibits the largest Φ_f_ among these four dyes, we do not observe dual fluorescence. It is noteworthy to emphasize that in polar solvents (MeOH, DMF, and DMSO), emission of **7-BgCoum** and **9-BgCoum** is below the detection limit. Fluorescence intensity of **6-BgCoum** in DMF and DMSO is low but on the same level as that in ACN. On the other hand, **8-BgCoum** in accordance to previous studies^57,58^ is strongly emissive across the solvents polarity scale (Table S1).

The photophysical properties of these novel benzo[*g*]coumarins ought to be compared with known benzo[*f*]coumarin and benzo[*h*]coumarin possessing NMe_2_ group (Figure 1).^56,59^ In analogy to methyl 9-(dimethylamino)-benzo[*f*]coumarin-2-carboxylate, **6-BgCoum** has negligible emission in DMSO and in DMF, whereas its λ_abs_^max^ = 360/427 nm (Tables 1, and S1). In the case of **7-BgCoum** and **9-BgCoum**, fluorescence in these solvents is below any detection limit. Also, methyl 8-(dimethylamino)-benzo[*h*]coumarin-3-carboxylate has similar absorption and emission data. The dual emission has not, however, been observed for these previously reported, π-expanded coumarins. Moreover, their emission in low-polarity solvents is less bathochromically shifted in comparison to that of **6-BgCoum**, **7-BgCoum**, and **9**-**BgCoum**.

Overall, these π-expanded aminocoumarin fall into two subgroups based on their photophysical behavior: (1) *type i* dyes, which include **6-** and **8-BgCoum**, that show strong CT emission and high-amplitude CT-absorption bands with relatively week or completely undetected double fluorescence; and (2) *type ii* dyes, which include the weakly fluorescent **7-** and **9-BgCoum**, that show dual fluorescence with substantial contributions from the short-wavelength bands and CT absorption with quite small extinction coefficients (Figure 2).

The photostability of the four benzo[*g*]coumarins was studied in toluene using a xenon light source (Figure S5). All investigated dyes displayed superb stability as demonstrated by comparing with coumarin 153. In particular, **9-BgCoum** displayed the highest photostability. Even prolonged exposure to a light source did not cause any noticeable changes in the absorption spectrum of **9-BgCoum**.

Using UV–vis absorption allows estimating the solubility of benzo[*g*]coumarins in H_2_O. The results show that **6-BgCoum** (3.5 μM) is the most water-soluble, followed by **9-BgCoum** (2.5 μM), **7-BgCoum** (2.5 μM), and **8-BgCoum** (0.2 μM).

### Electrochemical Properties and Redox Energy Gaps

Cyclic-voltammetry (CV) analysis provides estimates of the reduction potentials of the dyes and their oxidized forms, i.e.,  and , respectively.^68^ For the four aminocoumarins in different solvents,  ranges between −1.6 and −1.2 V vs SCE and —between 0.8 and 1.2 V vs SCE, making them moderately good electron acceptors and electron donors. Among them, the *type i***8-BgCoum** stands out as the worst electron acceptor with  about 100 mV more negative than those of the other three dyes. Similarly, the *type ii***7-BgCoum** appears to be the best electron donor with the smallest  (see Table S3). At position 8, thus, the electron-donating amine has the strongest effect on the lowest unoccupied molecular orbital (LUMO), and at position 7—on the highest occupied molecular orbitals (HOMO).

While it should be used with caution, Koopmans’ theorem correlates  and  with the LUMO and HOMO energy levels, respectively, of the dyes.^69^ The redox, or electrochemical, energy gaps, ε_EC_ = *F*, of the four π-expanded aminocoumarins range between 2.2 and 2.5 eV, corresponding to about 500 to 560 nm, which is between the CT absorption and emission bands (see Figure S9 in the Supporting Information). That is, ε_EC_ of the four aminocoumarins is similar to their zero-to-zero energy, ε_00_, corresponding to the optical HOMO–LUMO gaps. Therefore, the S_0_ → S_1_^(CT)^ and S_1_^(CT)^ → S_0_ radiative transitions involve predominantly the HOMOs and the LUMOs of these dyes. Since ε_00_ is not much smaller than ε_EC_, the electron–hole electrostatic interactions in the emissive CT do not provide significant stabilization, which concurs with a large extent of CS.

### Computational Analysis: How Important is the Ester Group?

To elucidate the origin of the dual fluorescence and the nature of the excited states responsible for the short-wavelength emission bands, we resort to ab initio calculations of the four aminocoumarins, implemented at the MP2/ADC(2)/cc-pVDZ level of theory using the Turbomole 7.3 software package.^70−75^ It is advantageous for the computational studies to truncate the alkyl chain of the ester substituent to a methyl. In addition to the gas-phase studies, employing the conductor-like screening model (COSMO) allows us to introduce DCM as a solvating medium.^76^

**Figure 3:**
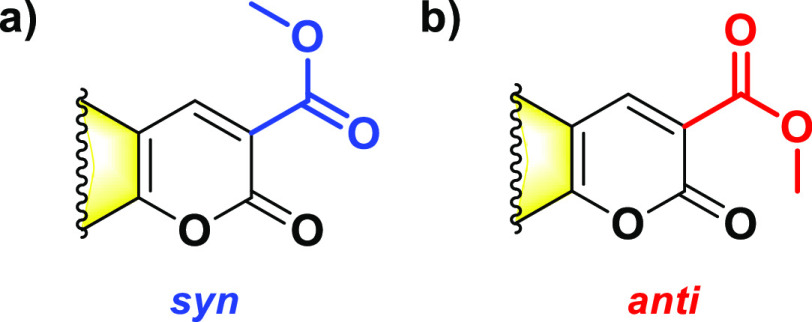
Ester-defined *syn* and *anti* conformers.

Ground-state optimizations in the gas phase leads to the emergence of two distinct conformers of the **BgCoum** structures with different orientations of the esters attached to their pyrone rings in position 3 (Figure 1): (1) *syn*, with the C=O carbonyl bonds of the ester and the lactone pointing in the same direction, and (2) *anti*, with the ester and lactone C=O bonds oriented in the opposite directions (Figure 3). Electronic conjugation between the ester and the aromatic ring appears to warrant a preference for planar conformations.

**Figure 4:**
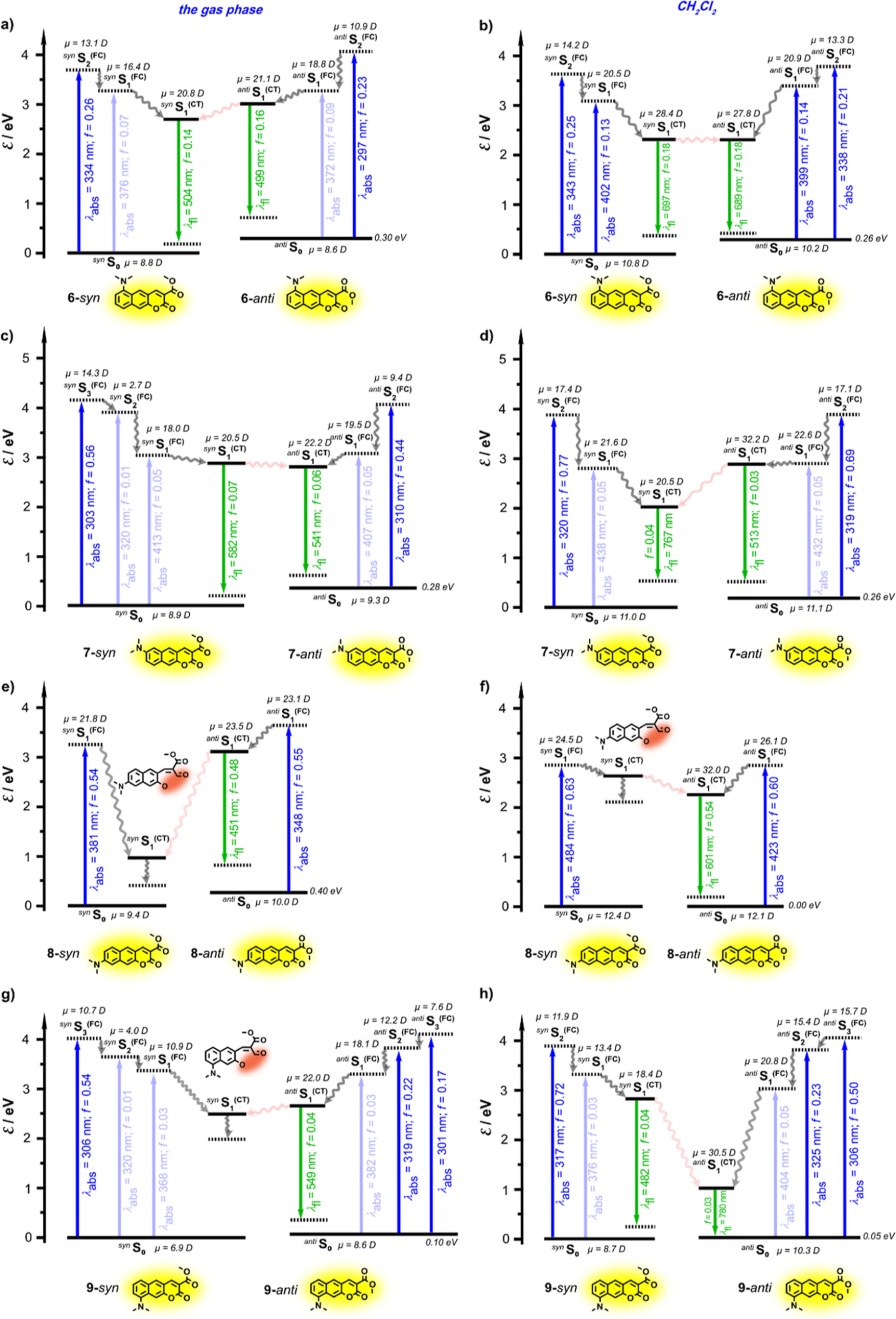
Jablonski energy diagram for **6-BgCoum**, **7-BgCoum**, **8-BgCoum**, and **9-BgCoum** planar *C*_*s*_ structures and respective S_*n*_–S_0_ density difference maps computed at MP2/ADC(2)/cc-pVDZ level of theory. Solid lines represent vertical excitation/emission energies of a given state computed at the respective ground/excited state minimum. The dashed black line represents vertical energy of the ground state computed at the optimized geometry of the respective compound. Numbers denote relevant energy in eV.

The *syn* conformers are overall more stable than the *anti* ones, precluding the latter from always providing detectable contributions to the ground-state optical absorption spectra. In the *anti* structures, the ester methyl crowds the carbonyl oxygen of the lactone. Since the methyl hydrogens are not acidic enough, this spatial proximity should not lead to hydrogen bonding but rather to steric hindrance that destabilizes the *anti* geometries. In the gas phase, the ground-state energy levels of the *anti* conformers are higher than those of the *syn* ones (Figure 4a,c,e,g). Introducing DCM as a solvating medium decreases this energy difference (Figure 4), which still leads to negligibly small equilibrium population of ***anti*-6-BgCoum** and ***anti*-7-BgCoum** in DCM, amounting to only about 0.003% at room temperature (Figure 4b,d). Conversely, the S_0_ populations of the *syn* and *anti* conformers of **8-BgCoum** in DCM are practically equal (Figure 4f), and that of ***anti*-9-BgCoum** in DCM amounts to 12% (Figure 4h). These trends suggest that for **6-BgCoum** and **7-BgCoum**, showing dual fluorescence, the excited-state dynamics appears to originate from photoexcitation of only their *anti* conformers.^77^ Nevertheless, **7-BgCoum** in DCM displays a short-wavelength fluorescence band (Figure 2b) that ought to originate from its *syn* conformer. A possible transition from the S_2_ state of the *syn* conformer to the S_1_ state of the *anti* one can account for the observation. Such estimates of conformer populations extracted from calculated energies of their ground-state structures, however, warrant caution because the inherent error of these computational methods can exceed 0.2 eV. This uncertainty offers an alternative explanation, i.e., the energy differences between the *anti syn* conformers of **7-BgCoum** can very well be significantly smaller than 10*k*_B_*T* as the computational results show.

Implementing vertical excitations on **8-BgCoum** structures in the gas phase and DCM reveals allowed S_0_ → S_1_^(FC)^ transitions with oscillator strengths, *f*, exceeding 0.5 and similar energies for the two conformers (Figure 4e,f), which is consistent with the large single absorption band observed between 400 and 500 nm (Figure 2c). The relaxation of the S_1_^(FC)^ state of the low-energy *syn* conformer leads to breaking of the C–O bond of the lactone and opening of the pyrone ring (Figure 4e,f). Coumarins are prone to such a ring-opening photoreaction, which provides a pathway for non-radiative decay competing with the radiative deactivation. The pyrone ring in unsubstituted coumarin is the most susceptible to this photoreaction according to calculations and spectroscopic evidence.^78^

Introducing DCM solvation medium does not prevent the ring opening of the excited-state ***syn*-8-BgCoum**. Nevertheless, the solvation substantially stabilizes the relaxed ***anti*-8-BgCoum** S_1_ structure which, with an electric dipole moment μ of 32 D, has a substantial CT character. This polarity-induced drop of the energy of the S_1_^(CT)^ state of ***anti*-8-BgCoum** below that of ***syn*-8-BgCoum** S_1_ state opens pathways for preventing the ring-opening reaction by *syn*-to-*anti* rotation of the ester group in the S_1_ manifold.

These computational finding are in an excellent agreement with the spectroscopic results. For *n*-hexane, offering solvating conditions close to those of the “non-polar” gas phase, the relatively small Φ_f_ of **8-BgCoum** (Φ_f_ ≈ 0.1, Table 1) is consistent with an efficient ring-opening pathway responsible for the fluorescence quenching. Even a slight increase in solvent polarity, decreases *k*_nr_ by 3 orders of magnitude resulting in quantitative Φ_f_ (Table 1). A further increase in medium polarity lowers the energy of the highly polarized S_1_^(CT)^ state resulting in the observed bathochromic shifts in the fluorescence of **8-BgCoum**. This polarity-induced drop of the S_1_^(CT)^ energy brings it close to that of the ground state and opens efficient non-radiative IC pathways via back CT, which is consistent with the observed decrease in Φ_f_ for polar solvents. In order to evaluate if the replacement of methyl group with other substituents can alter ring-opening pathway responsible for fluorescence quenching, we computed analogs of **8-BgCoum** possessing alternative ester groups (see Supporting Information). It turned out the same trend is also predicted for ethyl or benzyl esters for which only ***anti*-8-BgCoum** are expected to be emissive.

Computationally, the other *type i* dye, **6-BgCoum**, shows similar behavior to that of **8-BgCoum**. For the ***syn*-6-BgCoum**, the oscillator strength of the S_0_ → S_1_ transition is about two-to-four times smaller than that for the S_0_ → S_2_ one (Figure 4a,b). This finding is consistent with its absorption spectra of **6-BgCoum** where the UV features dominate, showing larger amplitudes than the band at around 400 nm that extends into the visible region (Figure 2a). The transitions from the ground to the S_1_^(FC)^ states for both conformers lead to doubling their dipoles, which further increase upon relaxation to the emissive S_1_^(CT)^ structures. Upon relaxation of their S_1_^(FC)^ states, however, neither of the **6-BgCoum** conformers undergoes ring opening. Instead, the relaxed S_1_^(CT)^ states of **6-BgCoum** are prone to radiative deactivation to S_0_ with *f* > 0.1 and similar transition energies for the ***syn*** and ***anti*** conformers. Furthermore, the energies of the vertical S_1_^(CT)^ → S_0_^(FC)^ transitions substantially decrease with DCM solvation (Figure 4b). These computational results are consistent with the experimentally observed single-fluorescence band of **6-BgCoum** that exhibits a pronounced solvatochromism (Figure 2a). Our computational findings, however, do not preclude the possibility of solvents, such as THF and PrBu, to induce different S_1_^(CT)^ → S_0_^(FC)^ transition energies for the ***syn*** and ***anti*** conformers, resulting in the observed dual fluorescence of **6-BgCoum** in such media (Figure 2a).

The two *type ii* dyes, **7-BgCoum** and **9-BgCoum**, show quite weak S_0_ → S_1_^(FC)^ vertical transitions with *f* between 0.01 and 0.05 (Figure 4c,d,g,h), which is consistent with the immensely small amplitudes observed for the 400 nm bands in their absorption spectra (Figure 2). That is, S_0_ → S_2_^(FC)^ and S_0_ → S_3_^(FC)^ transitions dominate the absorption spectra of **7-BgCoum** and **9-BgCoum** (Figures 2a,d and 4c,d,g,h). In the gas phase, each of these two dyes show only a single radiative transition, corresponding to a single fluorescence band for non-polar solvents: (1) from the S_1_^(CT)^ → S_0_^(FC)^ of ***syn*-7-BgCoum**, because the S_1_^(CT)^ state of ***anti*-7-BgCoum** is energetically unaccusable; and (2) from the S_1_^(CT)^ → S_0_^(FC)^ of ***anti*-9-BgCoum**, because the S_1_ state of ***syn*-9-BgCoum** undergoes ring opening (Figure 4c,g).

Introducing DCM solvation to **7-BgCoum** and **9-BgCoum** stabilizes their S_1_^(CT)^ states and makes the energies of the S_1_^(CT)^ → S_0_^(FC)^ transitions substantially different for the ***syn*** and ***anti*** structures. The energy gap between the S_1_^(CT)^ and S_0_^(FC)^ states of the ***syn*-7-BgCoum** is smaller than that of the ***anti*-BgCoum** (Figure 4d). Although the ground-state population of the ***anti*-7-BgCoum** appear to be negligibly small, excited-state ***syn***–***anti*** transformation allows access to the ^***anti***^S_1_^(CT)^ state from the ***syn*** manifold. For the **9-BgCoum**, on the other hand, the S_1_^(CT)^ → S_0_^(FC)^ transition energy of the ***anti*** conformer is smaller than that of the ***syn***. These computational results for the *type ii* dyes in DCM agree well with the observed double fluorescence that they produce.

Overall, the *type ii* dyes, **7-BgCoum** and **9-BgCoum**, are weakly fluorescent, as apparent from the small Φ_f_ and *k*_f_ values obtained for them from steady-state and time-resolved spectroscopy studies (Table 1). Consistently, the calculated oscillator strengths for the radiative–deactivation transitions of **7-BgCoum** and **9-BgCoum** (with and without solvation) are relatively small, i.e., the values of *f* range between 0.03 and 0.07 (Figure 4c,d,g,h). Indeed, the rate of radiative deactivation of the higher energy conformer has to be comparable to the rate of transitions between the *syn* and *anti* structures for the double fluorescence to be detectable.

**Figure 5:**
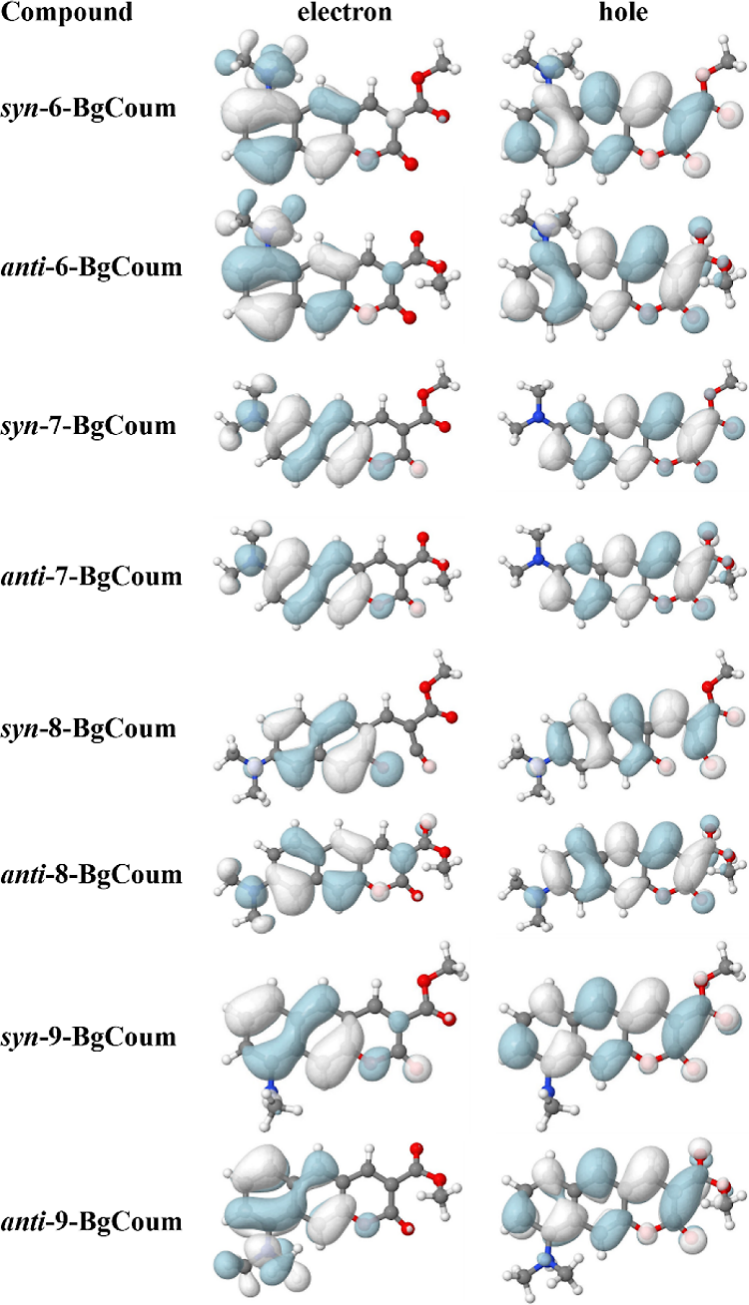
Natural transition orbitals (NTOs) for the radiative S_1_→S_0_ deactivation of the *anti* and *syn* conformers of the four benzo[g]coumarins, obtained from the optimized S_1_ excited state computed at ADC(2)/cc-pVDZ level of theory.

Examining the NTOs for the S_1_^(CT)^ → S_0_^(FC)^ transitions reveals that for the *type i* dyes the electron and the hole orbitals extend over the NMe_2_ substituent. For the *type ii* dyes, however, only the hole has distribution over NMe_2_ and the electron does not (Figures 5, and S14). Hence, enhancing the CS between the amine and the coumarin rings for the *type ii***BgCoum** derivatives decreases *f*, *k*_f_, and Φ_f_.

Overall, the position of the amine, changing its electronic coupling with the coumarin rings, governs the fluorescence efficiency of the π-expanded aminocoumarins. Conversely, the conformational degrees of freedom of the ester substituent on the pyrone ring govern the formation of multiple populations of emissive CT excited states responsible for the observed dual fluorescence.

Even though the quantum yields of the short-wavelength fluorescence, Φ_f_^SW^, are small (Table 1), the detectable emission from the upper excited states implies that they live long enough to drive useful processes such as charge transfer and energy transfer. The estimated radiative-decay rate constants, *k*_r_, hardly exceeds 10^7^ s^–1^ for the regioisomers that manifest dual emission. The oscillator strengths for the S_1_ → S_0_ transitions are relatively small and similar for the *syn*- and *anti*-conformers of each compound (Table 1, Figure 3). These considerations suggest sub-nanosecond non-radiative deactivation of the conformers with higher lying S_1_ states, rendering them feasible for driving picosecond reactions efficiently.

### Effects of Rigidity of the Solvating Micro Environment

**Figure 6:**
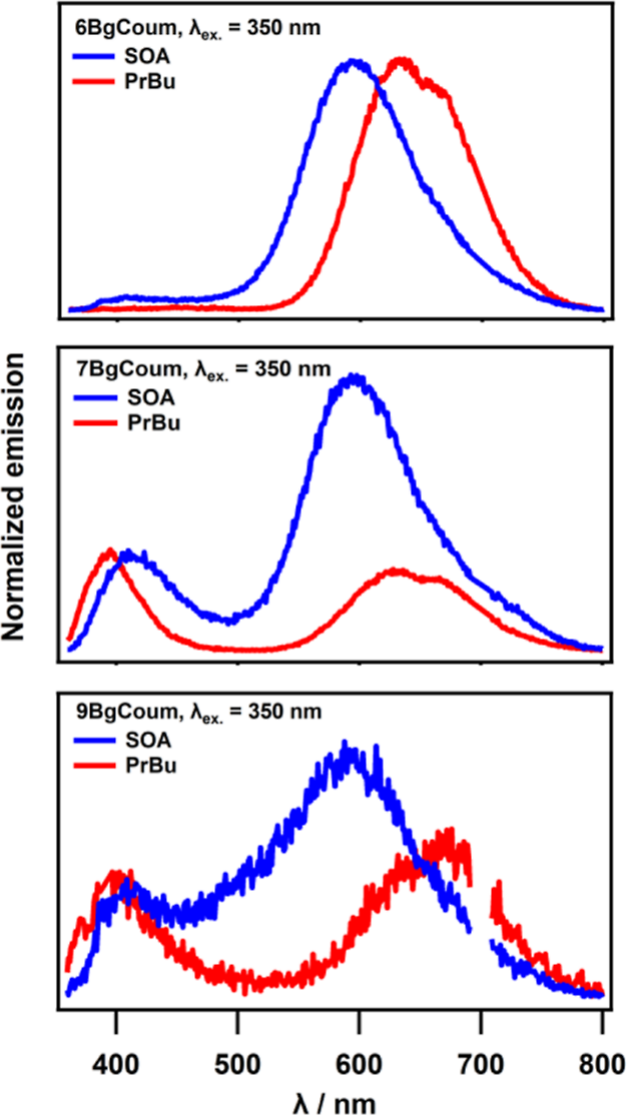
Emission spectra for **6-BgCoum**, **7 BgCoum**, and **9-BgCoum** in liquid propyl butyrate (PrBu) and solid sucrose octaacetate (SOA). Samples were excited at 350 nm in both media. A few crystals of compounds were dissolved in SOA while it was heating and then cooled down to obtain solid sample with appropriate isomer.

To elucidate which photoinduced transitions depend on large-ampliated conformational changes, we examine the effects of the rigidity of the solvating media on the dye photophysics. Sucrose octaacetate (SOA) forms transparent glass at room temperature making it a good choice as a solid solvent for optical spectroscopy tests.^15,79^ The polarity of SOA is similar to that of PrBu, which induces double fluorescence as a solvent of **6-**, **7-**, and **9-BgCoum** (Figure 6, Table 1).

Transferring these dyes from the liquid PrBu to the solid SOA medium enhances the quantum yields not only of their principal long-wavelength fluorescence, ϕ_f_^(LW)^, but also of the short-wavelength emission, ϕ_f_^(SW)^. Twisting the dimethylamine substituents away from the plane of the aromatic ring decreases the NTO overlap and the rates of radiative deactivation. Therefore, the SOA-induced enhancement of ϕ_f_^(LW)^ and ϕ_f_^(SW)^ can originate from suppressing the NMe_2_ dihedral rotation around its bond with the aromatic ring. Suppressing the rotation of the ester substituent in the solid medium presents another cause for the observed increase in ϕ_f_^(SW)^, but not in ϕ_f_^(LW)^, because transitions between the ***anti*** and ***syn*** conformers provide a non-radiative pathway for the decay of the upper emissive CT state.

The photophysical characteristics **8-BgCoum**, i.e., Φ_f_, τ, *k*_f_, and *k*_nr_, are quite similar for SOA and PrBu (Table 1). The SOA-induced suppression of the NMe_2_ twist should enhance *k*_f_. The prevention of the ester rotation and the *syn*-to-*anti* transition in the solid SOA, on the other hand, should leave the ring opening as the only pathway of deactivation resulting in substantial fluorescence quenching, which is not what the experimental results show. The rigid solvating medium, however, can also affect the efficiency of the ring opening and prevent the breaking of the lactone bond, which opens a pathway of radiative deactivation of the *syn* conformer responsible for the observed fluorescence of **8-BgCoum** in SOA (Figure S4e).

For **6-BgCoum**, on the other hand, SOA enhances ϕ_f_^(SW)^ by an order of magnitude and ϕ_f_^(LW)^ by a factor of 5 and decreases *k*_nr_ of the lower emissive CT state by a factor of 4 (Table 1). The effects of SOA on the photophysics of **7-BgCoum** are comparable to those on **6-BgCoum** (Table 1). Therefore, the dihedral rotation of NMe_2_ and the ester appear to play a key role in the excited-state dynamics of **6-BgCoum** and **7-BgCoum**.

The effects of the solid medium are the most pronounced for the weakly fluorescent **9-BgCoum** where the enhancement of ϕ_f_^(SW)^ and ϕ_f_^(LW)^ exceed an order of magnitude (Table 1). That is, large-amplitude conformational changes contribute significantly to the non-radiative deactivation of **9-BgCoum** in liquid solvents.

The observed short-wavelength fluorescence of **7-BgCoum** for SOA appears to contradict the computational findings. The energy level of the *anti* conformer is more than 10*k*_B_*T* above that of the *syn* one for room temperature (Figure 4d). Therefore, the photophysical phenomena should originate from excitation of *syn*-**7-BgCoum**, which dominates the ground-state population. The solid SOA medium precludes any *syn* to *anti* conformational transitions within the short lifetimes of the excited states of **7-BgCoum**. Indeed, the calculations are not for SOA solvent. Nevertheless, the results for the gas phase and for DCM are quite similar and SOA is less polar than DCM. Hence, it is safe to assume that the results for the S_0_ energies in SOA should be similar to those in DCM and in the gas phase. Another consideration involves the preparation of the SOA samples that require melting of the medium at about 90 °C, which increases *k*_B_*T*, but only by about 6 meV, which would hardly affect the change the populations with energy differences exceeding 0.2 eV. Based on these considerations, recalling that the inherent error of the employed ab initio methodology can exceed 0.2 eV, it is safe to assume that the calculated energy difference between the *anti* and *syn* ground states is considerably less than 0.26 eV.

### Solid-State Photophysics

**Figure 7:**
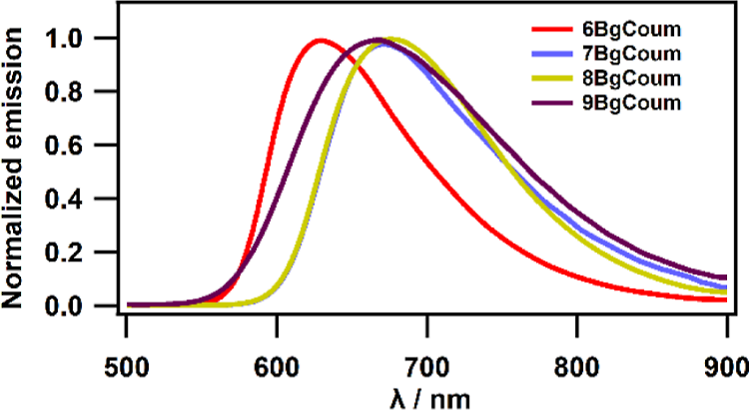
Emission spectra of all regioisomers of benzo[*g*]coumarins in solid-state. Powder samples were excited at 320 nm (except of **9BgCoum**—λ_ex_ = 400 nm).

In addition to the restrictive nature of solid media, excitonic coupling plays an important role when the dyes are packed together in crystals. In the solid state, each of the four dyes shows a single fluorescence band with Φ_f_ comparable to that for toluene (Table 2, Figure 7). Specifically, **7-BgCoum** exhibits the smallest Φ_f_ among the four dyes, and **6-BgCoum**—the highest, which is similar to their behavior in liquid solvents (Table 1).

**Table 2:** Quantum Yield of Fluorescence of benzo[*g*]coumarin Isomers in Powder

cmpd	λ_em_^max^ [nm]	Φ_f_ [%]
**6BgCoum**	625	26.0
**7BgCoum**	668	4.0
**8BgCoum**	676	16.0
**9BgCoum**	668	0.83

The relatively large Φ_f_ values that we obtain for the powder samples are consistent with the rigidity of the crystal packing, along with the inherently low polarity of the solid media because of the elimination of the orientational polarization. This polarity decrease in the solid state is consistent with the hypsochromically shifted emission of the powder samples in comparison with their fluorescence in liquid solutions. For example, **6-BgCoum** in acetonitrile shows an emission maximum at 755 nm, while in the solid-state, it is at 625 nm (Tables 1 and 2). The same is the case for **7-BgCoum**, i.e., λ_em_^(max)^ = 700 nm for THF, and λ_em_^(max)^ = 668 nm for solid state.

**Figure 8:**
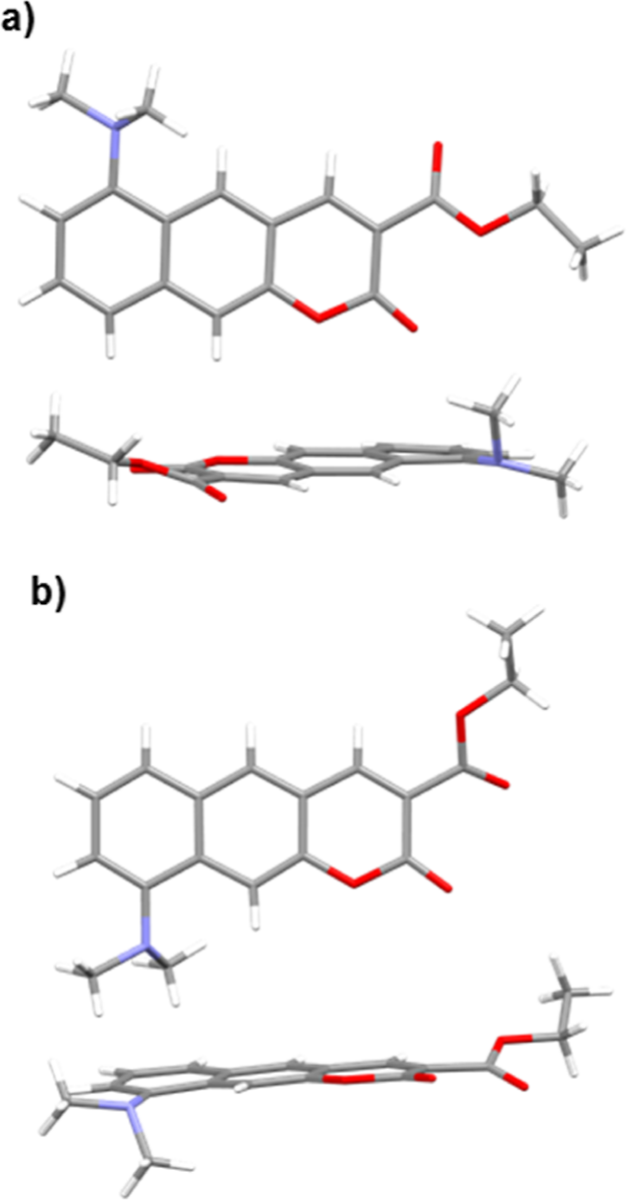
Structures of (a) **6-BgCoum** and (b) **9-BgCoum** from single crystal X-ray diffraction measurement, representing conformation of CO_2_Et group and non-planarity of NMe_2_ group (CCDC 2253992–2253995).

It is important to keep in mind that the crystal packing does not necessarily represent the conformational-equilibrium preference in solution phase. While the crystals of **9-BgCoum** contain its *syn* conformer reflecting the liquid-phase thermodynamics, the crystals of **6-BgCoum** comprise its *anti* conformer (Figure 8). The ethyl substituent of the ***anti*-6-BgCoum** in the crystals, however, points away from the lactone carbonyl oxygen avoiding steric hindrance, which is different from what the computational results show for the gas phase and the DCM medium. Overall, the crystals of each of the compounds contain a single conformer, precluding the possibility for dual fluorescence from these solid-state samples, which concurs with the observed emission spectra. This finding further confirms that the observed dual fluorescence for solvents with intermediate polarity originates from populations of conformers with different S_1_^(CT)^ → S_0_^(FC)^ transition energies.

In addition to the increased rigidity and the decreased polarity of the solvating media in solid state, along with the “conformational purity,” the crystalline packing provides conditions for intermolecular excitonic coupling that affects the observed spectral features. As previously reported for the parent compound **7-BgCoum**, stacking these molecules in the head-to-tail arrangements produces aggregates with overall *C*_*i*_ symmetry.^65^ The excitonic-coupling (Davydov) splitting for dimer models of such aggregates is estimated from the S_0_ → S_1_ and S_0_ → S_2_ vibrationless bands. For **7-BgCoum** and **9-BgCoum**, the excitonic coupling is 0.5 and 0.3 eV, respectively, suggesting a larger stabilization of the ^1^A_g_ state for these compounds. This state has a vanishing transition dipole moment to the ground state due to symmetry rules. Thus, for these dimers the lowest absorbing/emitting state is the ^1^A_u_ one, with low oscillator strength (less than 0.03). For **6-BgCoum**, however, the excitonic coupling is estimated to be small, and the emission from S_2_ (^1^A_u_) is expected at about 2.0 eV (*f* ≈ 0.2). This finding may correlate to the intense emission band around 700 nm observed experimentally for this benzo[*g*]coumarin. In contrast to the *type i* dyes in crystalline state, therefore, the *type ii***BgCoum** derivatives appear to show strong excitonic coupling.

## Conclusions

With the recent increase in chromophores showing “anti-Kasha” behavior, we show that conformers involving auxiliary substituents, such as ester groups on the pyrone rings of π-expanded aminocoumarins, can play a defining role in producing long-lived populations of excited states with different energy levels. For the observed dual fluorescence, it is, indeed, crucially important for the rates of the transitions between the two emissive excited states to be comparable to the rates of radiative deactivation of the upper of these two states. Varying solvent polarity and viscosity allows achieving such conditions that set paradigms for the pursuit of photosensitizers that effectively drive reactions from high-energy electronically excited states.

## Methods

### Synthesis

All reported NMR spectra (^1^H NMR and ^13^C NMR) were recorded on Varian 500 or 600 MHz and Bruker 500 MHz spectrometer. Chemical shifts (δ; ppm) were determined with TMS as the internal reference, *J* values are presented in Hz. Mass analyses in high resolution (HRMS) were obtained via electron ionization (EI) or electrospray ionization (ESI) source and a EBE double focusing geometry mass analyzer. Chromatography was performed on silica gel 60 (230–400 mesh) and thin layer chromatography was performed on TLC plates (Merck, silica gel 60 F_254_).

### General Procedure for the Preparation of BgCoums from Aldehydes

Diethyl malonate (245 μL, 1.2 equiv, 1.56 mmol) was added dropwise via a syringe to a vigorously stirred solution of **4** (280 mg, 1.3 mmol) in EtOH (5 mL). To the resulting mixture was added a catalytic amount of piperidine (20 μL) and the reaction was stirred at reflux for 4 h. After cooling to an ambient temperature, the solid was filtrated and washed with cold EtOH to afford the corresponding product as a powder.

### Steady State Optical Spectroscopy

Spectroscopic grade solvents were purchased from Sigma-Aldrich and used as obtained. For optical studies, solutions of molecules at low concentrations, about few micromoles per liter, were used to avoid dimerization or reabsorption effects. All absorption and fluorescence spectra were taken at room temperature. Steady-state absorption spectra are recorded in a transmission mode using Shimadzu UV-3600i Plus (Japan) and JASCO V-670 (Tokyo, Japan) spectrophotometers. Fluorescence spectra were recorded with the FS5 (Edinburgh Instruments, Edinburgh, UK), the FluoroLog-3 (Horiba-Jobin-Yvon, Edison, NJ, USA) spectrofluorometers, and the FLS 1000 Edinburgh Instruments (Edinburgh, UK) with integrating sphere and corrected for the spectral response sensitivity of the photodetector. The FluoroLog-3, which is equipped with a pulsed diode laser (λ = 406 nm, 200 ps pulse full width at half maximum, FWHM) and a TBX detector, was also employed for time-correlated single-photon counting (TCSPC) measurements.

### Computational Studies

Transition energy (Δ*E*), oscillator strength (*f*), dipole moment (μ), leading electronic configurations, relevant molecular orbitals of *syn*, and *anti*-conformations of **6-BgCoum** to **9-BgCoum** computed with ADC(2)/cc-pVDZ method at the ground state MP2 equilibrium. Solvent effects were considered within the COSMO approximation using dichloromethane (DCM) as solvent.

Remaining detailed methods are found in the Supporting Information.

## Supporting Information Available

Synthetic procedures, ^1^H and ^13^C NMR spectra, photophysical, electrochemical, theoretical, and crystallographic data (PDF)

## Abbreviations

CT: charge transfer
ISC: intersystem crossing
FC: Franck–Condon
SOA: sucrose octaacetate.
