# Supplementary material for: Conformation of the Ester Group Governs the Photophysics of Highly Polarized Benzo[g]coumarins
Source: JACS Au. 2023 Jun 26;3(7):1918–30. doi: 10.1021/jacsau.3c00169 (PMC10369411; doi:10.1021/jacsau.3c00169)
Supplement: Supplementary file 1 — au3c00169_si_001.pdf [file au3c00169_si_001.pdf]

Supporting Information for

# Conformation of Ester Group Governs the Photophysics of Highly Polarized Benzo[*g*]coumarins

Kamil Szychta,[†] Beata Koszarna,[†] Marzena Banasiewicz,[∆] Andrzej Sobolewski,[∆] Omar O'Mari,[θ] John A. Clark,[θ] Valentine I. Vullev, [θ£]* Cristina A. Barboza[∆§]* and Daniel T. Gryko[†]*

[†]Institute of Organic Chemistry of Polish Academy of Sciences, Kasprzaka 44/52, 01-224 Warsaw, Poland

[∆]Institute of Physics of Polish Academy of Sciences, Al. Lotników 32/46, 02-668 Warsaw, Poland

[§]Department of Physical and Quantum Chemistry, Faculty of Chemistry, Wroclaw University of Science and Technology, Wroclaw, Poland

[θ]Department of Bioengineering Department of Bioengineering, University of California, Riverside, California 92521, United States

[£]Department of Chemistry, Department of Biochemistry, and Materials Science and Engineering Program, University of California, Riverside, California 92521, United States

## Table of Contents

1. Materials and general procedures 2 – 5
2. Optical spectroscopy 6 – 15
3. Electrochemistry methods and data 16 – 21
4. Crystallographic data 22 – 23
5. Computational results 24 – 32
6. Cartesian coordinates 33 – 37
7. $^{1}H$ and $^{13}C$ NMR spectra 42 – 49
8. References 54

## 1. Materials and general procedures

All reported NMR spectra ($^1$H NMR and $^{13}$C NMR) were recorded on Varian 500 or 600 MHz and Bruker 500 MHz spectrometer. Chemical shifts (δ; ppm) were determined with TMS as the internal reference, *J* values are presented in Hz. Mass analyzes in high resolution (HRMS) were obtained via electron ionization (EI) or electrospray ionization (ESI) source and a EBE double focusing geometry mass analyzer. Chromatography was performed on silica gel 60 (230-400 mesh) and thin layer chromatography was performed on TLC plates (Merck, silica gel 60 $F_{254}$).

**Synthesis**

**6-(Dimethylamino)naphthalen-2-ol** (**2a**). To a 250 ml round-bottom pressure flask equipped with magnetic stirring bar sodium metabisulfate (14.26 g, 75 mmol), 2,6-dihydroxynaphthalene (2.4 g, 15 mmol), dimethylamine (8.7 ml, 30 mmol, 40 % solution in water) and $H_2O$ (6.4 ml) were added. The reaction was heated at 150 °C for 1.5 h. After cooling to room temperature, the mixture was extracted with $CH_2Cl_2$. The organic layer was separated, dried over anhydrous $Na_2SO_4$ and purified by column chromatography (silica gel, hexane/AcOEt, 4:1) to afford product as white precipitate. Yield 1.54 g (55 %). $R_f$ = 0.31 (hexane/AcOEt, 4:1). M. p. 116 °C. $^1$H NMR (500 MHz, $CDCl_3$) δ 7.57 (d, *J* = 8.7 Hz, 1H), 7.55 (d, *J* = 9.1 Hz, 1H), 7.16 (dd, *J* = 9.0, 2.4 Hz, 1H), 7.05 – 6.98 (m, 2H), 6.93 (d, *J* = 2.0 Hz, 1H), 4.87 (br s, 1H), 2.99 (s, 6H). $^{13}$C{$^1$H} NMR (126 MHz, $CDCl_3$) δ 150.9, 147.4, 130.2, 128.1, 127.9, 127.1, 118.0, 117.8, 109.4, 107.6, 41.3. HRMS (EI): *m/z* calculated for $C_{12}H_{13}NO$: 187.0997 [$M^{\bullet+}$]; found: 187.1001.

**General procedure for protecting of hydroxy group.** Sodium hydride (1 g, 25 mmol, 60 % dispersion in mineral oil) was added in one portion to a stirred solution of aminonaphthalen-2-ol (15 mmol) in dry DMF (15 ml) under argon at 0 °C. After the gas evolution ceased, chloromethyl methyl ether MOM-Cl (1.4 ml, 18 mmol) was added. The resulting mixture was stirred at room temperature for 2 h and then quenched by the addition of water. DMF was removed under reduced pressure, and the residue was extracted with $CH_2Cl_2$. The combined organic layers were dried over anhydrous $Na_2SO_4$, concentrated under reduced pressure and purified by column chromatography (silica gel, hexane/AcOEt, 2:1).

**5-amino-2-(methoxymethoxy)naphthalene** (**2b**). According general procedure compound **2b** was *syn*thesized starting from 5-aminonaphthalen-2-ol (**1b**, 159 mg, 1 mmol), sodium hydride (67 mg, 1.67 mmol, 60 % dispersion in mineral oil), MOM-Cl (90 µl, 1.2 mmol) and DMF (1 ml). The final product was obtained as a colorless oil. Yield: 102.9 mg (50 %). $R_f$ = 0.61 (hexane/AcOEt, 2:1). $^1$H NMR (500 MHz, DMSO-$d_6$) δ 8.00 (d, *J* = 9.2 Hz, 1H), 7.24 (d, *J* = 2.5 Hz, 1H), 7.14 (t, *J* = 7.8 Hz, 1H), 7.07 (dd, *J* = 9.2, 2.5 Hz, 1H), 6.95 (d, *J* = 8.1 Hz, 1H), 6.53 (dd, *J* = 7.5, 0.9 Hz, 1H), 5.64 (s, 2H), 5.28 (s, 2H), 3.41 (s, 3H). $^{13}$C{$^1$H} NMR (126 MHz, DMSO-$d_6$) δ 155.5, 146.0, 136.6,

128.7, 125.4, 119.8, 117.3, 115.8, 111.1, 107.2, 95.1, 56.9. HRMS (EI): *m/z* calculated for $C_{12}H_{13}NO_2$: 203.0946 [$M^{•+}$], found: 203.0947.

**8-amino-2-(methoxymethoxy)naphtalene** (**2c**). The reaction of 8-aminonaphthalen-2-ol (**1c**, 2.39 g, 15 mmol) according to general procedure gave a colorless oil. Yield: 2.22 g (73 %). $R_f$ = 0.45 (hexane/AcOEt, 2:1). $^1$H NMR (500 MHz, $CDCl_3$) δ 7.73 (d, *J* = 8.9 Hz, 1H), 7.37 (d, *J* = 2.1 Hz, 1H), 7.27 (d, *J* = 8.1 Hz, 1H), 7.22 (dd, *J* = 8.9, 2.3 Hz, 1H), 7.20-7.16 (m, 1H), 6.77 (dd, *J* = 7.3, 0.7 Hz, 1H), 5.31 (s, 2H), 4.02 (br s, 2H), 3.53 (s, 3H). $^{13}$C{$^1$H} NMR (126 MHz, $CDCl_3$) δ 154.7, 141.2, 130.4, 130.1, 124.5, 124.4, 118.9, 118.6, 110.3, 104.1, 94.8, 56.0. HRMS (ESI): *m/z* calculated for $C_{12}H_{14}NO_2$: 204.1025 [$M+H^+$], found 204.1020.

**6-(dimethylamino)-2-(methoxymethoxy)naphthalene** (**3a**). According general procedure compound **3a** was *syn*thesized starting from 6-(dimethylamino)naphthalen-2-ol (**2a**, 187 mg, 1 mmol), sodium hydride (67 mg, 1.67 mmol, 60 % dispersion in mineral oil), MOM-Cl (90 µl, 1.2 mmol) and DMF (1 ml). The final product was obtained as a white precipitate. Yield: 189 mg (82 %). M. p. 66 °C. $R_f$ = 0.59 (hexane/AcOEt, 6:1). $^1$H NMR (500 MHz, $CDCl_3$) δ 7.61 (d, *J* = 9.1 Hz, 1H), 7.59 (d, *J* = 8.9 Hz, 1H), 7.29 (d, *J* = 2.3 Hz, 1H), 7.18-7.11 (m, 2H), 6.91 (d, *J* = 2.2 Hz, 1H), 5.24 (s, 2H), 3.52 (s, 3H), 3.00 (s, 6H). $^{13}$C{$^1$H} NMR (126 MHz, $CDCl_3$) δ 152.6, 147.7, 130.9, 127.8, 127.7, 127.6, 119.2, 117.4, 110.4, 107.1, 94.9, 55.9, 41.2. HRMS (EI): *m/z* calculated for $C_{14}H_{17}NO_2$: 231,1259 [$M^{•+}$]; found: 231,1267.

**General procedure for methylation of amine group.** A 100 ml round-bottom pressure flask equipped with a magnetic stirring bar was charged with $K_2CO_3$ (2.76 g, 20 mmol) and methyl *p*-toluenesulfonate (1.64 g, 8.8 mmol). The flask was filled with argon and the solution of amino-2-(methoxymethoxy)naphthalen **2** (825 mg, 4 mmol) in dry acetonitrile (20 ml) was added by syringe. The resulting mixture was refluxed for 30 h. After cooling to room temperature the excess of $K_2CO_3$ was filtrated off and acetonitrile was removed under reduced pressure. The crude material was dissolved in $CH_2Cl_2$ and all inorganic compounds were filtrated off. The organic solution was concentrated under reduced pressure and purified by column chromatography (silica gel, hexane/AcOEt, 4:1).

**5-(dimethylamino)-2-(methoxymethoxy)naphthalene** (**3b**). The reaction of 5-amino-2-(methoxymethoxy)naphthalene (**2b**, 825 mg, 4 mmol) according to general procedure gave an oil. Yield: 591 mg (60 %). $R_f$ = 0.67 (hexane/AcOEt, 4:1). $^1$H NMR (500 MHz, $CDCl_3$) δ 8.16 (d, *J* = 9.2 Hz, 1H), 7.42 (d, *J* = 8.2, 1H), 7.39 – 7.32 (m, 2H), 7.22 (dd, *J* = 9.2, 2.5 Hz, 1H), 6.96 (dd, *J* = 7.4, 0.8 Hz, 1H), 5.30 (s, 2H), 3.53 (s, 3H), 2.88 (s, 6H). $^{13}$C{$^1$H} NMR (126 MHz, $CDCl_3$) δ 154.9, 151.0, 136.0, 126.5, 125.9, 124.7, 122.1, 117.8, 112.3, 110.5, 94.5, 56.0, 45.2. HRMS (ESI): *m/z* calculated for $C_{14}H_{18}NO_2$: 232.1338 [$M+H^+$]; found: 232.1346.

**8-(dimethylamino)-2-(methoxymethoxy)naphthalene** (**3c**). The reaction of 8-amino-2-(methoxymethoxy)naphthalene (**2c**, 825 mg, 4 mmol) according to general procedure gave an oil. Yield: 712 mg (77 %). $R_f$ = 0.67 (hexane/AcOEt, 4:1). $^1$H NMR (500 MHz, $CDCl_3$) δ 7.77 (d, *J* = 2.3 Hz, 1H), 7.74 (d, *J* = 8.9 Hz, 1H), 7.45 (d, *J* = 8.1 Hz, 1H), 7.29 – 7.24 (m, 1H), 7.21 (dd, *J* = 8.9, 2.5 Hz, 1H), 7.05 (d, *J* = 7.6 Hz, 1H), 5.32 (s, 2H),

3.53 (s, 3H), 2.87 (s, 6H). $^{13}C\{^1H\}$ NMR (126 MHz, $CDCl_3$) δ 154.7, 150.1, 130.7, 129.9, 129.9, 123.9, 122.7, 118.5, 114.5, 107.1, 94.6, 56.1, 45.0. HRMS (EI): *m/z* calculated for $C_{14}H_{17}NO_2$: 231.1259 [$M^{\bullet +}$]; found: 231.1254.

**General procedure for direct formylation of dimethylamino-2-(methoxymethoxy)naphthalene.** The procedure was developed by modification of the previously know formylation method.[32] The solution of dimethylamino-2-(methoxymethoxy)naphthalene **3** (578 mg, 2.5 mmol) in $Et_2O$ (13 ml) was cooled to -20 °C and *t*-BuLi (2.2 ml, 3.75 mmol, 1.7 M in pentane) was added dropwise within 30 minutes via syringe. The mixture was stirred at -20°C for 2 h and dry DMF (6.4 ml, 82.5 mmol) was subsequently added dropwise. After stirring at -20°C for 1 h, the mixture was slowly treated with 5 M aqueous solution of HCl (10 ml) and stirring was continued at room temperature overnight. The reaction mixture was neutralized with saturated $NaHCO_3$ solution until the gas has evolved (to pH 7) and extracted twice with $CH_2Cl_2$. The organic fractions were combined, dried over anhydrous sodium sulfate and concentrated under reduced pressure. The residue was purified by column chromatography (silica gel, hexane/AcOEt 4:1) to afford compound **4**.

**7-(dimethylamino)-3-(methoxymethoxy)-2-naphthaldehyde** (**4a**). The reaction of 6-(dimethylamino)-2-(methoxymethoxy)naphthalene (**3a**, 693 mg, 3 mmol) according to general procedure gave compound **4a** as an orange solid. Yield: 460 mg (59 %). M. p. 150 °C. $R_f$ = 0.40 (hexane/AcOEt, 4:1). $^1H$ NMR (500 MHz, $CDCl_3$) δ 10.08 (s, 1H), 10.03 (s, 1H), 7.95 (s, 1H), 7.60 (d, *J* = 9.1 Hz, 1H), 7.31 (dd, *J* = 9.1, 2.6 Hz, 1H), 7.17 (s, 1H), 6.93 (d, *J* = 2.3 Hz, 1H), 3.03 (s, 6H). $^{13}C\{^1H\}$ NMR (126 MHz, $CDCl_3$) δ 196.9, 153.3, 147.5, 135.7, 131.7, 128.9, 127.3, 122.5, 121.6, 111.6, 107.2, 40.9. HRMS (EI): *m/z* calculated for $C_{13}H_{13}NO_2$: 215.0946 [$M^{\bullet +}$]; found: 215.0941.

**8-(dimethylamino)-3-(methoxymethoxy)-2-naphthaldehyde** (**4b**). The reaction of 5-(dimethylamino)-2-(methoxymethoxy)naphthalene (**3b**, 578 mg, 2.5 mmol) according to general procedure gave product **4b** an orange oil. Yield: 286 mg (53 %). $R_f$ = 0.51 (hexane/AcOEt, 6:1). $^1H$ NMR (500 MHz, DMSO-*d*$_6$) δ 10.60 (s, 1H), 10.44 (s, 1H), 8.60 (s, 1H), 7.49 – 7.44 (m, 1H), 7.41 (d, *J* = 8.2 Hz, 1H), 7.29 (s, 1H), 6.95 (d, *J* = 7.3 Hz, 1H), 2.87 (s, 6H). $^{13}C\{^1H\}$ NMR (126 MHz, DMSO-*d*$_6$) δ 193.6, 156.6, 153.3, 140.2, 130.7, 129.7, 123.8, 122.8, 121.6, 112.9, 112.1, 45.9. HRMS (EI): *m/z* calculated for $C_{13}H_{13}NO_2$: 215.0946 [$M^{\bullet +}$]; found: 215.0950.

**5-(dimethylamino)-3-(methoxymethoxy)-2-naphthaldehyde** (**4c**). The reaction of 8-(dimethylamino)-2-(methoxymethoxy)naphthalene (**3c**, 1.78 g, 7.7 mmol) according to general procedure gave an orange solid. Yield 1.41 g (85 %). M. p. 83 °C. $R_f$ = 0.60 (hexane/AcOEt, 4:1). $^1H$ NMR (500 MHz, $CDCl_3$) δ 10.29 (s, 1H), 10.08 (s, 1H), 8.12 (s, 1H), 7.68 (s, 1H), 7.53 (d, *J* = 8.3 Hz, 1H), 7.32 – 7.26 (m, 1H), 7.15 (dd, *J* = 7.4, 0.7 Hz, 1H), 2.86 (s, 6H). $^{13}C\{^1H\}$ NMR (126 MHz, $CDCl_3$) δ 196.6, 155.5, 149.8, 138.1, 134.1, 128.7, 124.3, 123.9, 122.0, 118.0, 109.1, 44.8. HRMS (ESI): *m/z* calculated for $C_{13}H_{14}NO_2$: 216.1025 [$M+H^+$]; found: 216.1028.

**General procedure for the preparation of BgCoum.** Diethyl malonate (245 µl, 1.2 eq, 1.56 mmol) was added dropwise via a syringe to a vigorously stirred solution of **4** (280 mg, 1.3 mmol) in EtOH (5 ml). To the resulting mixture was added a catalytic amount of piperidine (20 µl) and the reaction was stirred at reflux for 4 h. After cooling to

ambient temperature the solid was filtrated and washed with cold EtOH to afford the corresponding product as a powder.

**7-BgCoum**. The reaction of 7-(dimethylamino)-3-hydroxy-2-naphthaldehyde (**4a**) according to general procedure gave an orange powder. Yield 123 mg (31 %). $R_f$ = 0.40 (hexane/AcOEt, 2:1). M. p. 145 °C. $^1$H NMR (500 MHz, $CDCl_3$) δ 8.57 (s, 1H), 7.88 (s, 1H), 7.73 (d, *J* = 9.2 Hz, 1H), 7.55 (s, 1H), 7.33 (dd, *J* = 9.2, 2.5 Hz, 1H), 6.91 (d, *J* = 2.2 Hz, 1H), 4.43 (q, *J* = 7.1 Hz, 2H), 3.08 (s, 6H), 1.43 (t, *J* = 7.1 Hz, 3H). $^{13}$C{$^1$H} NMR (126 MHz, $CDCl_3$) δ 163.4, 157.2, 149.0, 148.4, 148.2, 132.0, 129.3, 128.3, 128.1, 120.5, 118.3, 118.1, 112.3, 105.7, 61.9, 40.6, 14.3. HRMS (EI): *m*/*z* calculated for $C_{18}H_{17}NO_4$: 311.1158 [$M^{\bullet+}$]; found: 311.1151.

**6-BgCoum.** The reaction of 8-(dimethylamino)-3-hydroxy-2-naphthaldehyde (**4b**) according to general procedure gave a red solid. Yield: 240 mg (59 %). $R_f$ = 0.39 (hexane/AcOEt, 2:1). M. p. 130 °C. $^1$H NMR (500 MHz, $CDCl_3$) δ 8.68 (s, 1H), 8.54 (s, 1H), 7.66 (s, 1H), 7.55 – 7.50 (m, 2H), 7.06 (dd, *J* = 6.0, 2.5 Hz, 1H), 4.44 (q, *J* = 7.1 Hz, 2H), 2.94 (s, 6H), 1.44 (t, *J* = 7.1 Hz, 3H). $^{13}$C{$^1$H} NMR (126 MHz, $CDCl_3$) δ 163.2, 157.0, 152.3, 150.7, 149.2, 137.7, 129.9, 128.1, 125.9, 122.1, 118.2, 116.9, 114.0, 112.8, 61.9, 45.3, 14.3. HRMS (EI): *m*/*z* calculated for $C_{18}H_{17}NO_4$: 311.1158 [$M^{\bullet+}$]; found: 311.1152.

**9-BgCoum.** The reaction of 5-(dimethylamino)-3-hydroxy-2-naphthaldehyde (**4c**) according to general procedure gave an orange precipitate. Yield 165 mg (41 %). $R_f$ = 0.50 (hexane/AcOEt, 2:1). M. p. 135 °C. $^1$H NMR (500 MHz, $CDCl_3$) δ 8.63 (s, 1H), 8.12 (s, 1H), 8.11 (s, 1H), 7.59 (d, *J* = 8.3 Hz, 1H), 7.43 (dd, *J* = 8.2, 7.5 Hz, 1H), 7.19 (d, *J* = 7.4 Hz, 1H), 4.44 (q, *J* = 7.1 Hz, 2H), 2.89 (s, 6H), 1.43 (t, *J* = 7.1 Hz, 3H). $^{13}$C{$^1$H} NMR (126 MHz, $CDCl_3$) δ 163.1, 157.0, 150.8, 150.3, 148.4, 131.9, 131.5, 131.0, 126.1, 123.3, 118.9, 117.6, 117.2, 109.9, 62.0, 44.9, 14.2. HRMS (ESI): *m*/*z* calculated for $C_{18}H_{17}NO_4Na$: 334.1055 [$M+Na^+$]; found: 334.1064.

## 2. Optical spectroscopy

**Steady state optical spectroscopy.**

Spectroscopic grade solvents were purchased from Sigma-Aldrich and used as obtained. For optical studies solutions of molecules at low concentrations, about few micromoles per liter, were used to avoid dimerization or reabsorption effects. All absorption and fluorescence spectra were taken at room temperature. Steady-state absorption spectra are recorded in a transmission mode using Shimadzu UV-3600i Plus (Japan) and JASCO V-670 (Tokyo, Japan) spectrophotometers. Fluorescence spectra were recorded with the FS5 (Edinburgh Instruments, Edinburgh, UK), the FluoroLog-3 (Horiba-Jobin-Yvon, Edison, NJ, USA) spectrofluorometers, and the FLS 1000 Edinburgh Instruments (Edinburgh, UK) with integrating sphere, and corrected for the spectral response sensitivity of the photodetector. The FluoroLog-3, which is equipped with a pulsed diode laser ($\lambda$ = 406 nm, 200-ps pulse full width at half maximum, FWHM) and a TBX detector, was also employed for time-correlated single-photon counting (TCSPC) measurements. Molar extinction coefficients, $\varepsilon$, were estimated from absorbance, $A$, of solution with known sample concentrations, $C$, in cuvettes with optical path length, $l$, using the Bouguer-Lambert-Beer law, $A = C{\cdot}\varepsilon{\cdot}l$. For estimating the fluorescence quantum yields, $\phi_f$, The absorbance at the excitation wavelength of all samples was kept in the range between 0.1 and 0.2. The values of $\phi_f$ were calculated from the steady-state absorption and emission spectra using references with know $\phi_f^{ref}$:

$$\phi_f = \phi_f^{ref} \times \frac{\int F(\lambda)d\lambda}{\int F^{ref}(\lambda)d\lambda} \times \frac{(1-10^{-A^{ref}(\lambda_{ex})})}{(1-10^{-A(\lambda_{ex})})} \times \left(\frac{n}{n^{ref}}\right)^2 \quad \text{(S1)}$$

where $F(\lambda)$ is the fluorescence intensity at wavelength $\lambda$; $A(\lambda_{ex})$ is the absorbance at the excitation wavelength; $n$ is the refractive index of the solvent; and the superscript "*ref*" indicated the values for the reference sample.

For calculating the total fluorescence quantum yields, $\phi_f$, of the four compounds, we excite at the second absorption band, i.e., at around 300 – 350 nm, integrate over the whole wavelength range of the fluorescence spectra, and for reference use quinine sulfate in 0.5 M sulfuric acid ($\phi_f^{QS}$ = 0.544). For the samples with two detectable emission bands, we estimate the fluorescence quantum yields of short-wavelength band, $\phi_f^{SW}$, from the same spectra by introducing in eq. S1 only the integrated emission of the short-wavelength band. For determining the fluorescence quantum yields of the long-wavelength bands, $\phi_f^{LW}$, we record emission spectra exciting at the first absorption band, i.e., at 400 – 450 nm, and use coumarin 153 in ethanol ($\phi_f^{C\text{-}153}$ = 0.546) as a reference.

These three fluorescence quantum yields extracted from the steady-state optical spectra, $\phi_f$, $\phi_f^{SW}$, and $\phi_f^{LW}$, relate to the quantum yield of the conformation change, $\phi_t$, responsible for the transition between *syn* and *anti* conformers:

$$\phi_f = \phi_f^{SQ} + \phi_f^{LW}\phi_t \quad \text{(S2a)}$$

where $\phi_f^{SW}$, $\phi_f^{LW}$ and $\phi_t$ can be expressed in terms of the rate constants of the excited state processes - $k_f^{SW}$, $k_f^{LW}$, $k_{nr}^{SW}$, $k_{nr}^{LW}$ and $k_t$:

$$\phi_f^{\mathrm{SW}} = \frac{k_f^{\mathrm{SW}}}{k_f^{\mathrm{SW}} + k_{nr}^{\mathrm{SW}} + k_{\mathrm{t}}}$$

(S2b)

$$\phi_{\mathrm{t}} = \frac{k_{\mathrm{t}}}{k_f^{\mathrm{SW}} + k_{nr}^{\mathrm{SW}} + k_{\mathrm{t}}}$$

(S2c)

$$\phi_f^{\mathrm{LW}} = \frac{k_f^{\mathrm{LW}}}{k_f^{\mathrm{LW}} + k_{nr}^{\mathrm{LW}}}$$

(S2d)

Because the SW states are too short-lived for measuring their lifetimes with TCSPC, we estimate the values of $k_f^{SW}$ using Einstein coefficient for spontaneous emission in terms of computationally obtained oscillator strengths, $f_{em}$, of the radiative transitions for the SW to ground states:

$$k_f^{LE} \approx \frac{2\pi v^2 e^2}{\varepsilon_0 m_e c^3} f_{em} \tag{3}$$

where $v$ – frequency of transition from SW to ground state, $e$ – electron charge, $\varepsilon_0$ – dielectric permittivity of vacuum, $m_e$ – mass of electron, and $c$ – speed of light in vacuum.

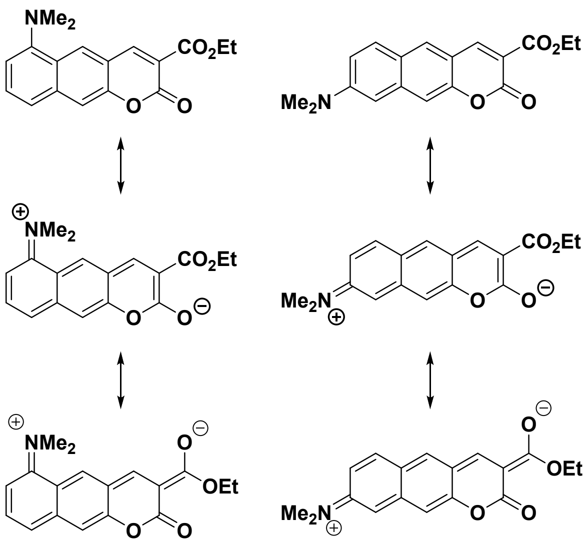

*weak mesomeric effect*

**6-BgCoum** **8-BgCoum**

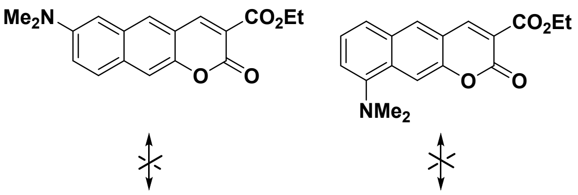

*none resonanse structures*

**7-BgCoum** **9-BgCoum**

**Fig. SF1.** Resonanse sturctures for all regioisomers of examined benzo[*g*]coumarins.

**Table ST1.** Results of photophysical studies in solvents and solid state for **6**, **7**, **8**, and **9BgCoum**.

| solvent | $\lambda_{abs}^{max}$ [nm] ($\varepsilon \cdot 10^3$ [$M^{-1}cm^{-1}$]) | $\lambda_{em}^{max}$ [nm] | $\Phi_f$ [%] | τ [ns] | SS [$cm^{-1}$] | $k_r \cdot 10^7$ [$s^{-1}$] | $k_{nr} \cdot 10^7$ [$s^{-1}$] |
|---|---|---|---|---|---|---|---|
| **6BgCoum** | | | | | | | |
| *n*-hexane | 355 (12.7), 406 (3.7) | 567 | 50.5 | 13.4 | 7000 | 3.76 | 3.69 |
| toluene | 360 (11.3), 416 (3.7) | 633 | 26.1 | 6.0 | 12000 | 4.33 | 12.3 |
| DCM | 360 (12.4), 427 (4.7) | 703 | 3.5 | 0.9 | 9200 | 12.2 | 96.7 |
| THF | 357 (11.4), 417 (4.0) | 409, 686 | 5.9 | 1.0 | 3560, 10700 | 3.35 | 91.9 |
| ACN | 355 (11.3), 415 (4.2) | 755 | 0.6 | 0.2 | 10850 | 2.94 | 465.0 |
| MeOH | 357 (11.2), 425 (3.7) | - | - | - | - | - | - |
| DMSO | 360 (10.1), 427 (4.1) | 766 | 0.7 | 0.24 | 10350 | 3.67 | 519.0 |
| DMF | 358 (10.2), 425 (4.0) | 753 | 0.7 | 0.19 | 10300 | 2.92 | 415.0 |
| propyl butyrate | 414 | 632 | 0.1 | 2.4 | 8300 | 0.38 | 41.3 |
| SOA | 422 | 592 | 5.6 | 7.16 | 6800 | 0.81 | 13.2 |
| solid state | 320 (ex.) | 625 | 26.0 | - | - | - | - |
| **7BgCoum** | | | | | | | |
| *n*-hexane | 334 (21.0) | 574 | 14.4 | 13.1 | 12500 | 1.10 | 6.54 |
| toluene | 341 (22.0), 464 (1.7) | 630 | 8.9 | 9.9 | 5700 | 0.82 | 9.27 |

| | | | | | | | |
|---|---|---|---|---|---|---|---|
| DCM | 340 (24.1), 475 (1.6) | 407, 703 | 0.62 | 1.5 | 4800, 6800 | 1.38 | 63.3 |
| THF | 336 (22.0), 463 (0.85) | 396, 700 | 0.95 | 3.9 | 4500, 7300 | 0.99 | 24.7 |
| ACN | 333 (25.0), 470 (1.6) | - | - | - | - | - | - |
| MeOH | 340 (22.0), 475 (1.5) | - | - | - | - | - | - |
| DMSO | 340 (21.3), 478 (1.4) | - | - | - | - | - | - |
| DMF | 338 (21.4), 470 (1.4) | - | - | - | - | - | - |
| propyl butyrate | 463 | 395, 636 | 0.54 | 5.27 | 5900 | 0.02 | 19.0 |
| SOA | 454 | 410, 593 | 2.1 | 12. 8 | 5200 | 0.05 | 7.8 |
| solid state | 320 (ex.) | 668 | 4.0 | - | - | - | - |
| **8BgCoum** | | | | | | | |
| *n*-hexane | 417 (25.2) | 480 | 11.0 | 0.76 | 2500 | 14.0 | 118.0 |
| toluene | 437 (33.4) | 510 | 98.0 | 4.2 | 3800 | 23.0 | 0.3 |
| DCM | 453 (27.4) | 565 | 99.9 | 4.65 | 4400 | 21.5 | 0.02 |
| THF | 438 (25.2) | 560 | 95.0 | 4.76 | 4900 | 20.0 | 1.1 |
| ACN | 445 (55.2) | 590 | 86.0 | 4.8 | 5500 | 17.0 | 2.9 |
| MeOH | 455 (23.3) | 600 | 53.0 | 3.31 | 5600 | 16.0 | 14.1 |
| DMSO | 457 (23.0) | 611 | 73.2 | - | 5500 | 17.5 | 6.4 |
| DMF | 450 (25.0) | 599 | 72.9 | - | 5500 | 17.0 | 6.3 |
| propyl butyrate | 434 | 542 | 34.0 | 4.38 | 4600 | 7.8 | 15.1 |
| SOA | 436 | 526 | 49.2 | 4.24 | 4600 | 11.6 | 12.0 |
| solid state | 320 (ex.) | 676 | 16.0 | - | - | - | - |
| **9BgCoum** | | | | | | | |
| *n*-hexane | 310 (22.0) | 586 | 8.3 | 6.3 | 15200 | 1.31 | 14.5 |
| toluene | 310 (21.5), 412 (1.7) | 660 | 0.90 | 1.6 | 9100 | 0.55 | 62.6 |
| DCM | 310 (21.5), 411 (1.8) | 390, 753 | 0.22 | 2.8 | 6600, 11000 | 0.27 | 35.0 |
| THF | 308 (23.9), 407 (1.4) | 410, 756 | 0.08 | 2.6 | 8100, 11300 | 0.031 | 38.22 |
| ACN | 307 (25.2), 413 (1.6) | - | - | - | - | - | - |
| MeOH | 306 (24.3) | - | - | - | - | - | - |
| DMSO | 294 (26.0), 410 (1.6) | - | - | - | - | - | - |
| DMF | 311 (23.0), 410 (1.6) | - | - | - | - | - | - |
| propyl butyrate | 402 | 412, 676 | 0.03 | 0.70 | 10100 | 0.02 | 142.8 |
| SOA | 407 | 409, 588 | 0.80 | 5.85 | 7600 | 0.21 | 16.9 |
| solid state | 400 (ex.) | 668 | 0.83 | - | - | - | - |

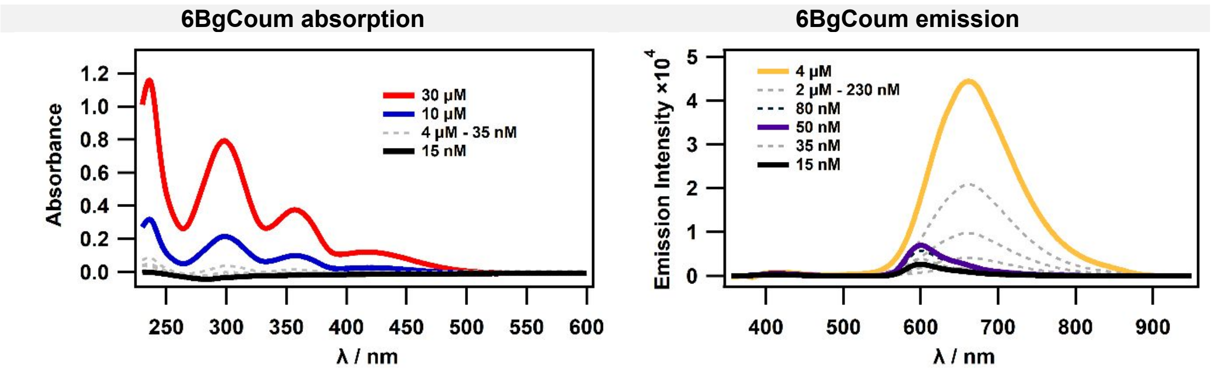

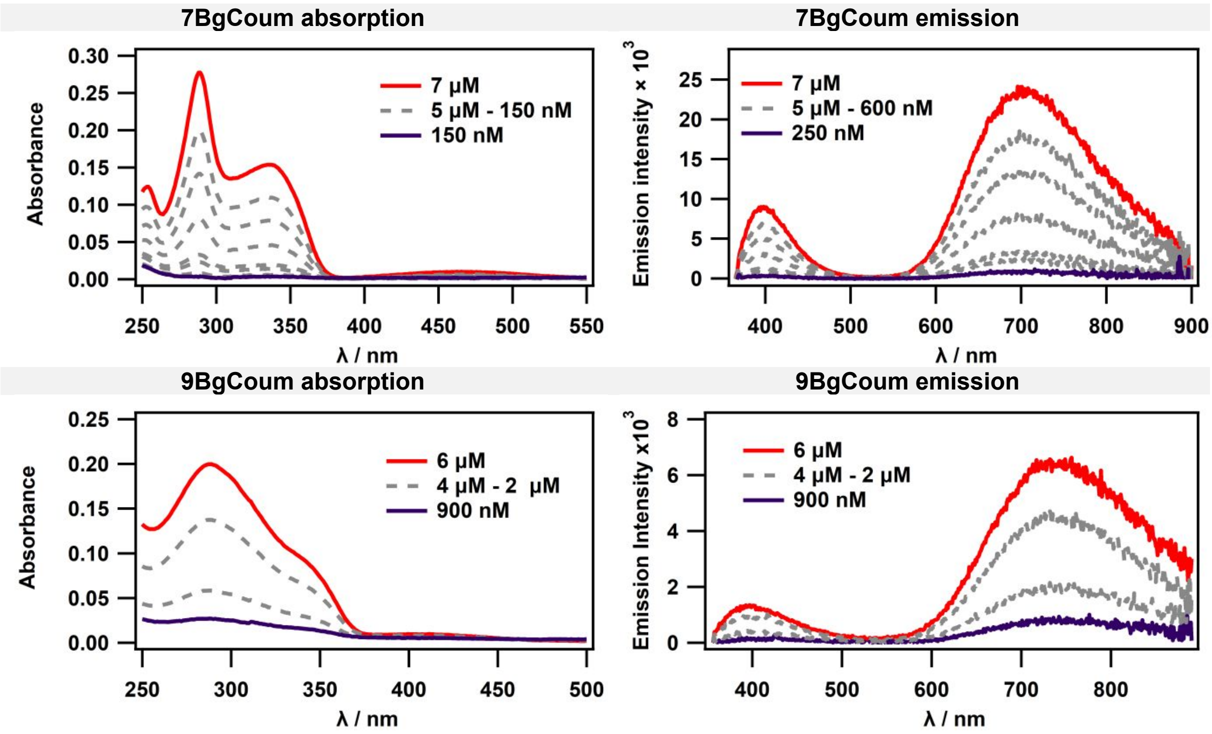


**Fig. SF2.** Concentration UV-Vis and emission studies on **6, 7** and **9BgCoum** in THF.

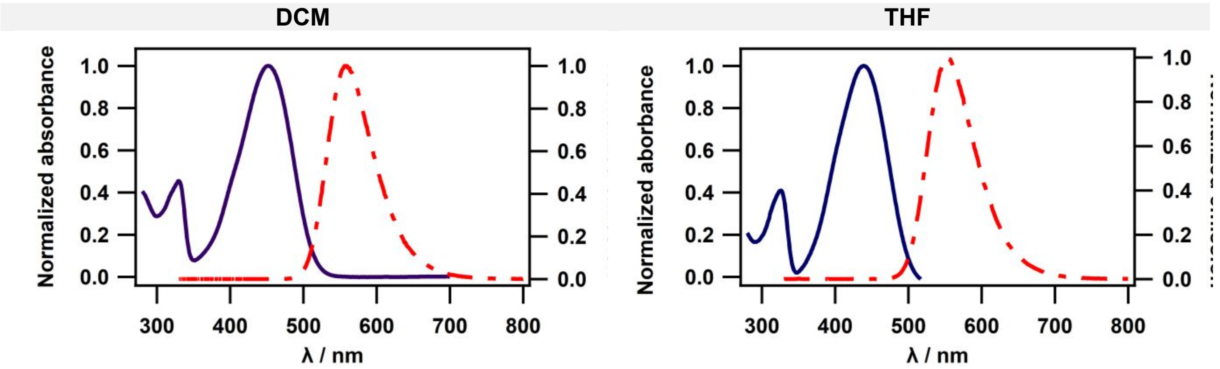


**Fig. SF3**. Steady-state optical studies on **8BgCoum** in DCM and THF ($\lambda_{ex}$ = 330 nm). Solid line represents absorption and dashed line – emission.

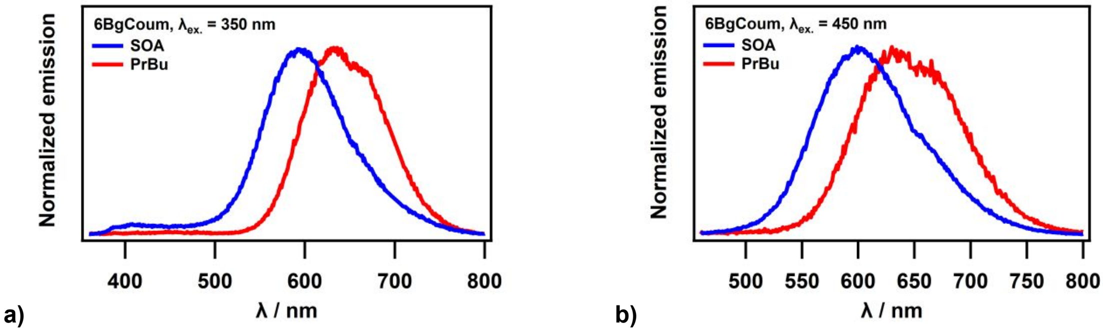

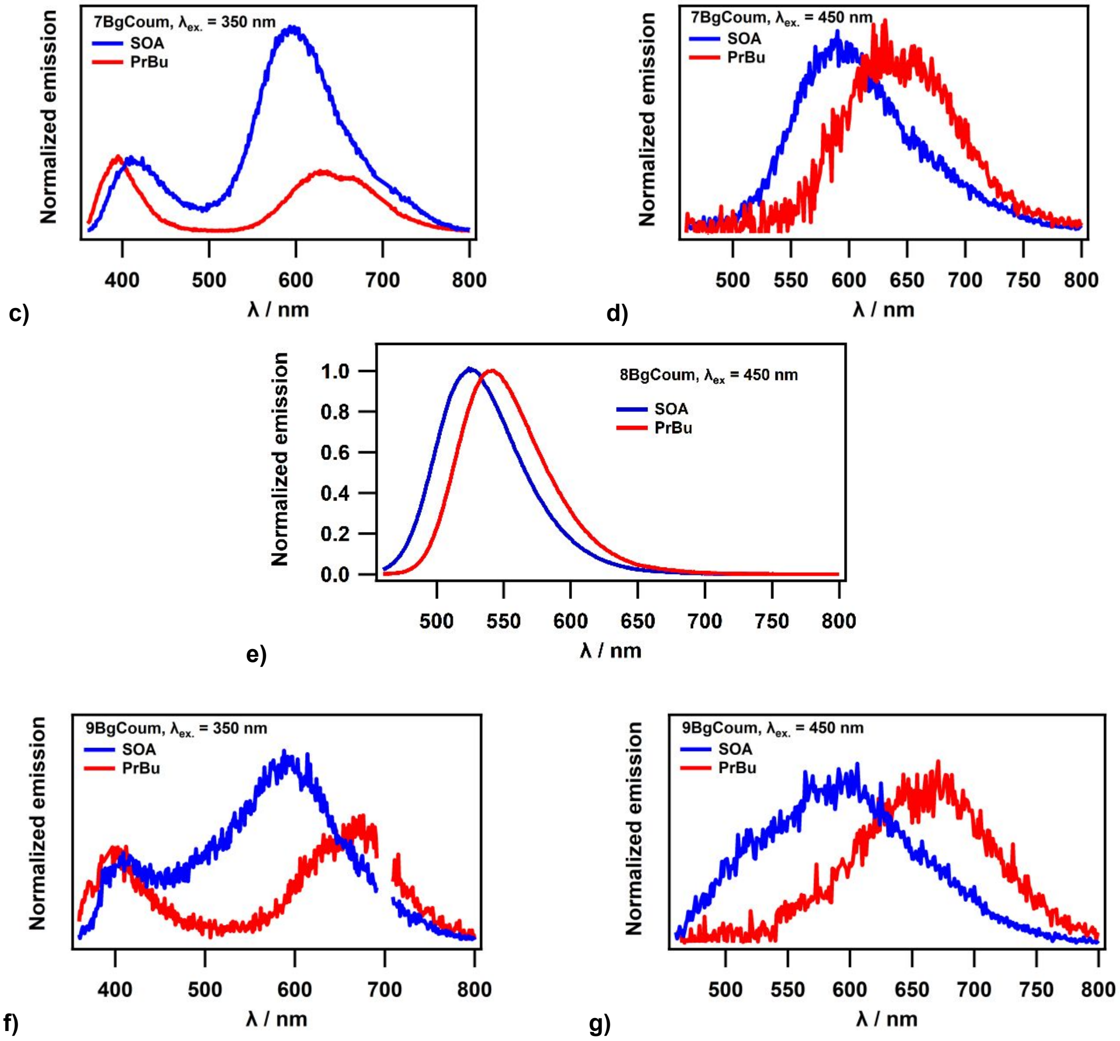


**Fig. SF4.** Emission spectra of benzo[g]coumarin isomers in propyl butyrate (PrBu) and sucrose octaacetate (SOA) scaled to the same intensity at short-wavelength band.

## Photostability

Photostability was examined using an Asahi Spectra Max-350 as a light source and a Perkin Elmer Lambda 25 UV/Vis spectrometer. It was determined through the variation in absorption of each sample at the appropriate absorption maximum wavelength ($\lambda_{abs}$) with respect to irradiation time. Methanol was selected as the solvent in all cases as this is closest solvent to aqueous media (important for biological imaging) that these compounds are soluble in. Concentrations giving similar optical densities (A ≈ 1) were used. Quartz cuvettes of samples were irradiated with a 300 W Xe lamp (Asahi spectra, MAX-350) equipped with a UV/Vis mirror module through a glass fiber. The absorption spectra were measured at appropriate times during the irradiation. Coumarin 153 (2,3,6,7-Tetrahydro-9-(trifluoromethyl)-1H,5H,11H-[1]benzopyrano(6,7,8-ij)quinolizin-11-one) was used as a reference.

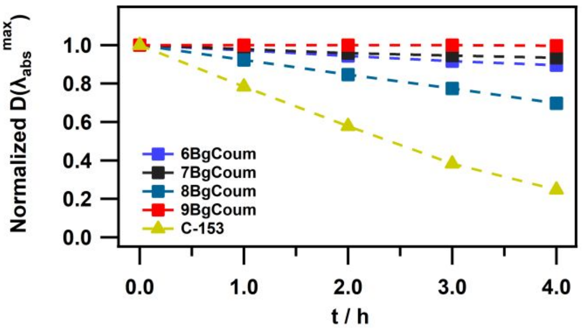


**Fig. SF5.** Photostability of each regioisomer of **BgCoum** in contrast to Coumarin 153 (**C-153**). The Y-axis represents the normalized value of absorbance at maximum of first intensive band ($D(\lambda_{abs}^{max})$) to the initial absorbance of sample before irradiation.

## Solubility in water

Firstly, the compound was dissolved in DMSO to prepare the stock solution ($10^{-2}$ M). Then the solution was diluted to $5*10^{-3}$ M and $5*10^{-4}$ M, and added to a quartz cuvette containing 3 ml of ultrapure water (Millipore Simplicity UV 18.2 MΩcm) by micropipette. In all cases, the concentration of DMSO in the sample was below 0.5%. The plot of fluorescence intensity and/or absorption against the compound concentration was linear at low concentration and showed saturation at higher concentration. The maximum concentration in the linear region was taken as the solubility.[1] The solubility of compound **6**, **7**, **8** and **9-BgCoum** in $H_2O$ is 3.5 μM, 0.5 μM, 0.2 μM and 2.5 μM, respectively.

**6BgCoum**

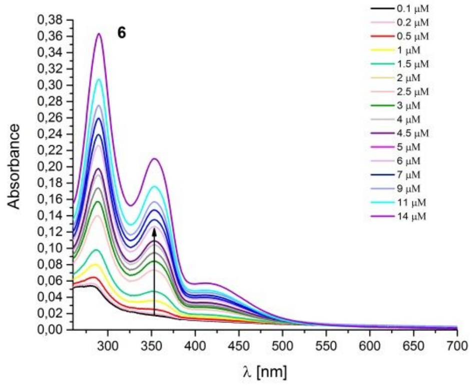


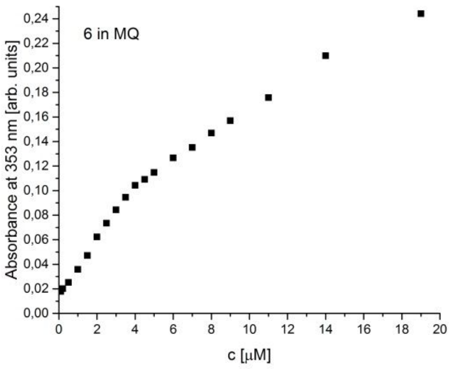

**7BgCoum**

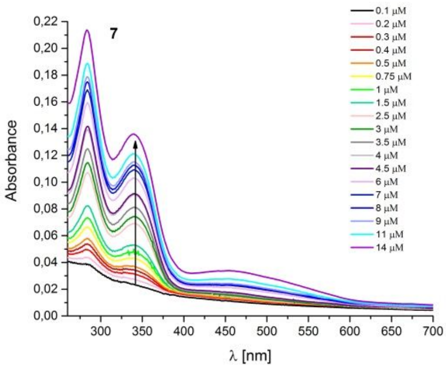


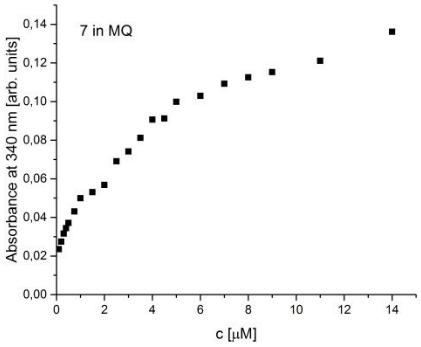


**8BgCoum**

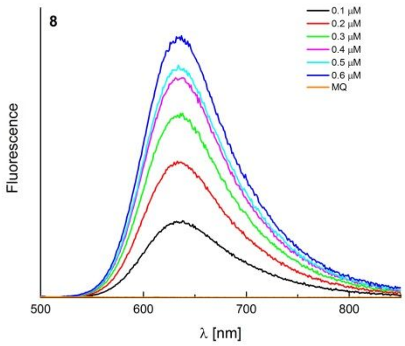


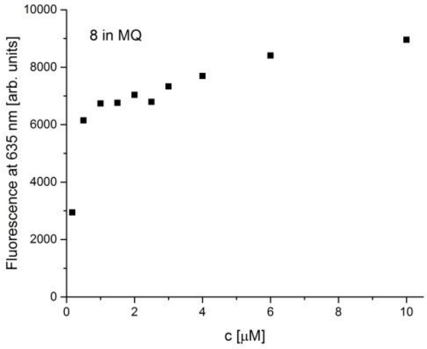


**9BgCoum**

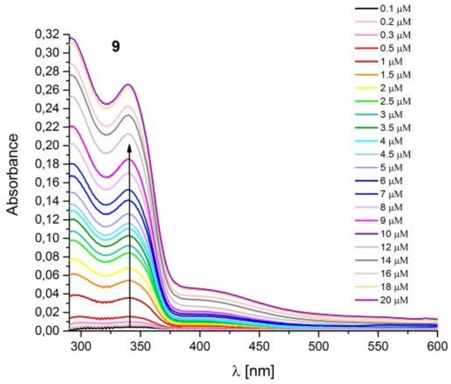


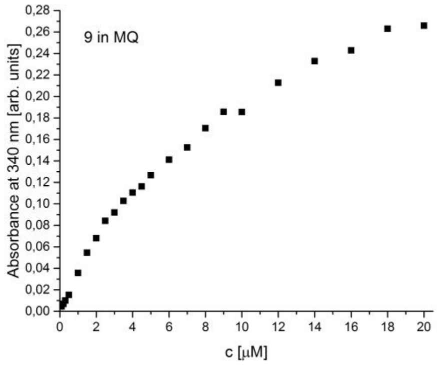


**Fig. SF6.** The solubility in water of examined benzo[g]coumarins. In cases of **6-**, **7-**, and **9-BgCoum**, there was measured absorbance, and in case of **8BgCoum** – emission intensity ($\lambda_{ex}$ = 450 nm).

### Time-resolved optical emission

Fluorescence decays were recorded using the FluoroLog-3 spectrofluorometer with its TBX detector running in a single-photon-counting mode. Selected neutral-density filters placed in front of laser excitation source ($\lambda_{ex}$ = 406 nm, pulse FWHM = 200 ps) allow controlling the intensity of the excitation light. All samples were purged with argon for 5-10 minutes prior to each measurement.

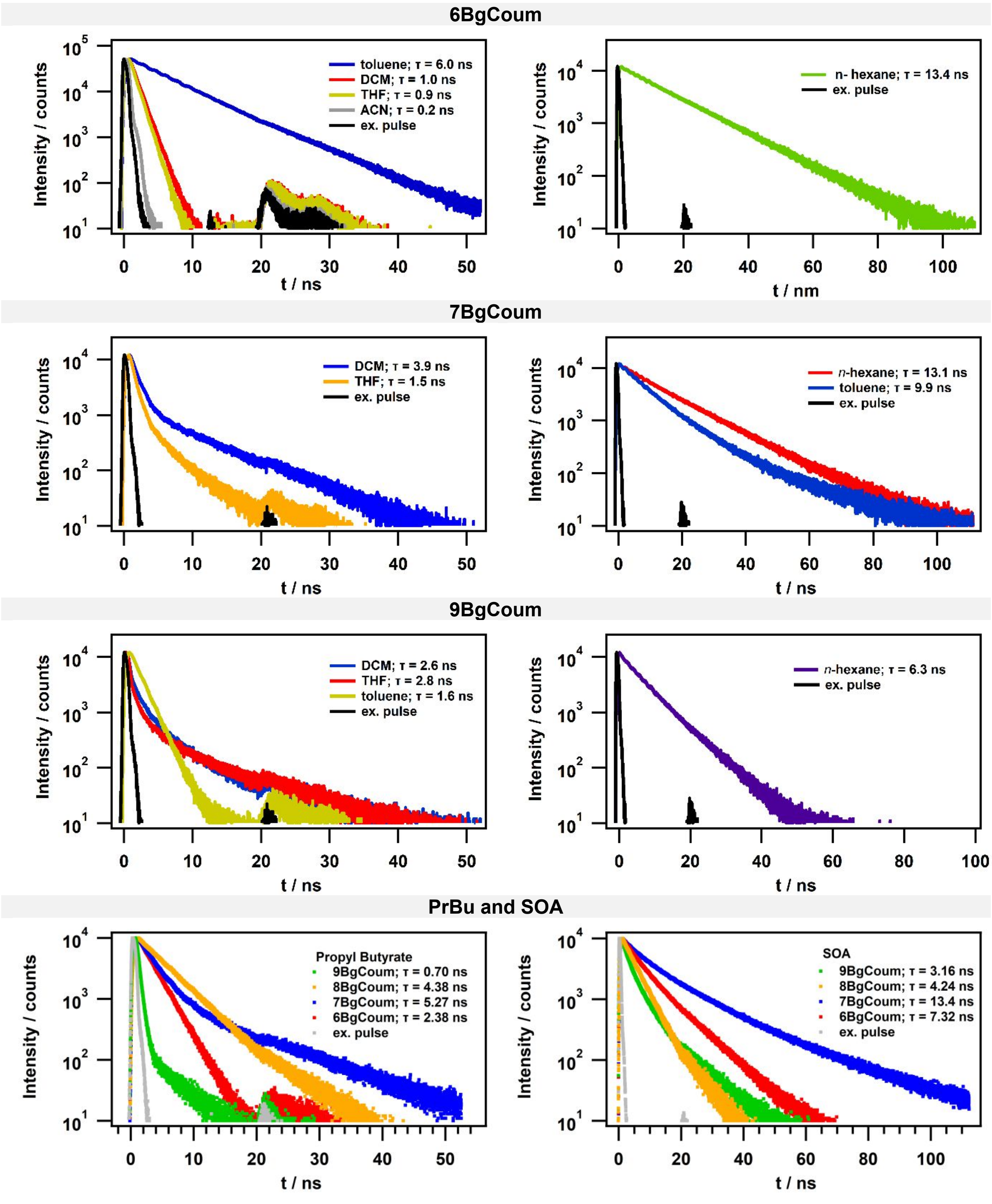


**Fig. SF7.** Lifetime measurements for **6**, **7**, **8** and **9BgCoum** recorded using Time-Correlated Single-Photon Counting (TCSPC). Photons were counted at emission maxima of all compounds (for samples which exhibits dual emission, there were being recorded only long-wavelength band) based on steady-state optical measurements.

Using a deconvolution algorithm, data were fitted with exponential-decay functions. For characterizing the multiexponential fits, we use intensity-averaged lifetimes, $\langle\tau\rangle = \left(\sum_i \alpha_i \tau_i^2\right)\left(\sum_i \alpha_i \tau_i\right)^{-1}$.

**Table ST2.** Results for multiexponential function fitting of decays in various solvents. There are not presented values for monoexponential fit in various solvents.

| Compound | Solvent | t [ns] | Contribution (normalized to 1) | Averaged decay time τ [ns] |
|---|---|---|---|---|
| **6BgCoum** | SOA | 6.520 | 0,85 | 7.160 |
| | | 9.610 | 0,15 | |
| **7BgCoum** | toluene | 7.612 | 0.92 | 9.912 |
| | | 20.631 | 0.08 | |
| | THF | 0.738 | 0.95 | 1.546 |
| | | 4.318 | 0.05 | |
| | DCM | 1.072 | 0.92 | 3.894 |
| | | 8.354 | 0.08 | |
| | PrBu | 2.360 | 0.89 | 5.270 |
| | | 10.400 | 0.11 | |
| | SOA | 4.670 | 0.37 | 12.800 |
| | | 14.400 | 0.63 | |
| **9BgCoum** | THF | 1.568 | 0.06 | 2.831 |
| | | 9.427 | 0.009 | |
| | | 0.174 | 0.94 | |
| | DCM | 1.922 | 0.10 | 2.614 |
| | | 9.317 | 0.01 | |
| | | 0.201 | 0.89 | |
| | PrBu | 0.387 | 0.99 | 0.700 |
| | | 4.510 | 0.01 | |
| | SOA | 2.970 | 0.92 | 5.850 |
| | | 13.400 | 0.08 | |

## 3. Electrochemistry methods and data

### Cyclic Voltammetry

Cyclic voltammetry is conducted using Reference $600^{\mathrm{TM}}$ Potentiostat/Galvanostat/ZRA (Gamry Instruments, PA, U.S.A.), connected to a three-electrode cell, as previously described.[2–4] Anhydrous aprotic solvents with different polarity, dichloromethane (DCM), tetrahydrofuran (THF), and benzonitrille (PhCN), are employed with different concentrations of $(n\text{-}C_4H_9)_4NPF_6$ as a supporting electrolyte. Prior to recording each voltammogram, the sample is extensively purged with argon while maintaining its volume constant by adding more of the anhydrous solvent. For each solvent, a set of voltammograms is recorded where the electrolyte concentration is increased from 25 mM to 200 mM in increments of 25 mM.

Voltammograms of the dyes show irreversibility and, in some cases, partial reversibility, suggesting for lifetimes of the formed radical ions that are comparable with or shorter than the timescales of the electrochemical measurements.[5] Therefore, the half-wave potentials, $E^{(1/2)}$, are determined from the zero point of the second derivatives of the voltammograms, $\partial^2 I/\partial E^2 = 0$ at $\partial E/\partial t$ = constant.[3] The voltammograms are recorded at a scan rate of [?] mV $s^{-1}$. To correct for potential drifts in the reference electrode (which is SCE, connected with the cell via a salt bridge), ferrocene is used as a standard ($E^{(1/2)} = 0.45 \pm 0.01$ V vs. SCE for $CH_3CN$ with 100 mM $(n\text{-}C_4H_9)_4NBF_4$).[5] Voltammograms of the ferrocene standard are recorded before and after each set of measurements.[6,7]

In some cases, the separation between the cathodic and anodic peak potentials is unusually large, which could originate from asymmetry in the reduction and oxidation processes at the electrode surface.[8] Nevertheless, re-oxidation of products of degradation of the radical anions can account for the positive shifts of the cathodic waves (in the negative scans), and re-reduction of degradation product of the radical cations – for the negative shifts of the anodic waves (in the positive scans). Furthermore, these coumarins often show two anodic peaks in the negative (reduction) scans or two cathodic peaks in the positive (oxidation) scans, which is consistent with the formation of new species at the surface of the working electrode. These features are similar, for example, to those observed for quinoline and anthracene derivatives, which form dimers during electrochemical reduction, where the second anodic peaks originate from the oxidation of dimeric anions.

| REDUCTION | OXIDATION |
| --- | --- |

**6BgCoum**

**DCM**

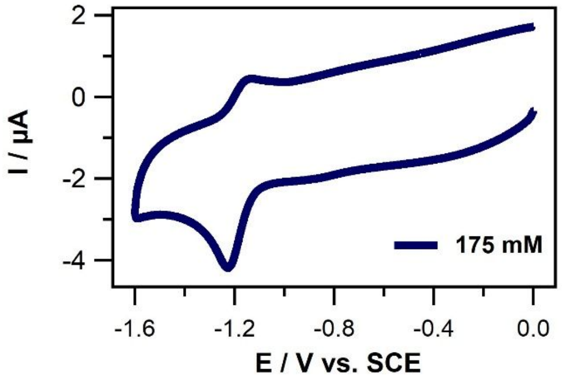


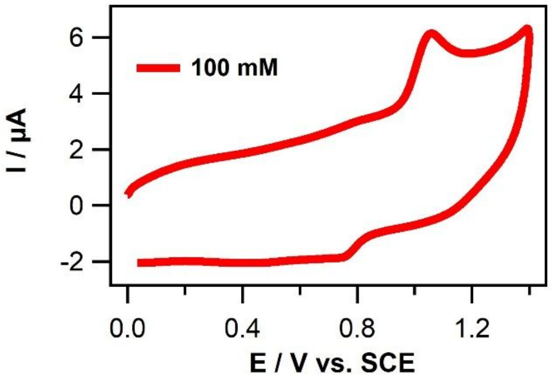


**THF**

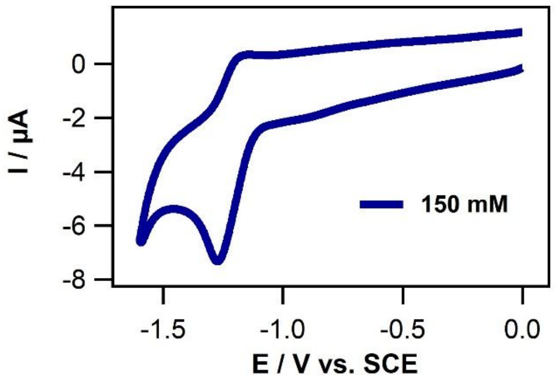


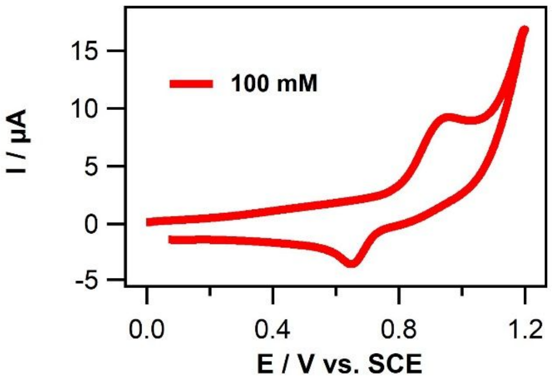


**PhCN**

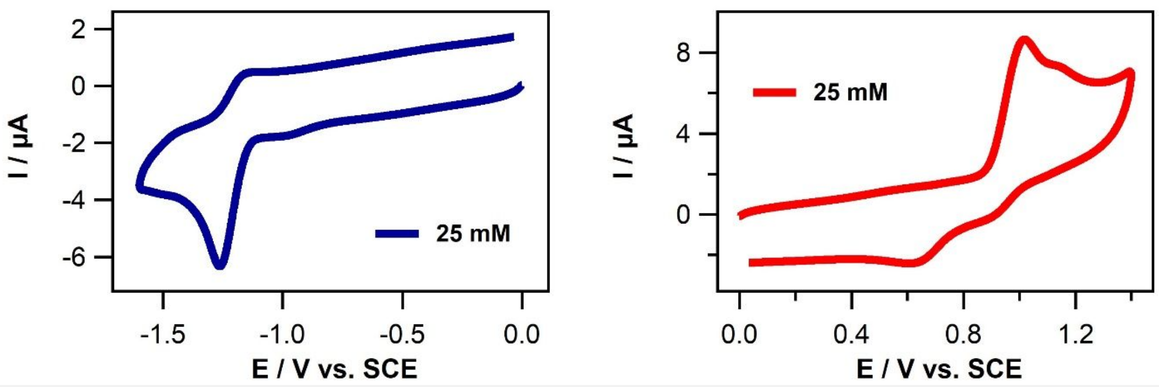


**7BgCoum**

**DCM**

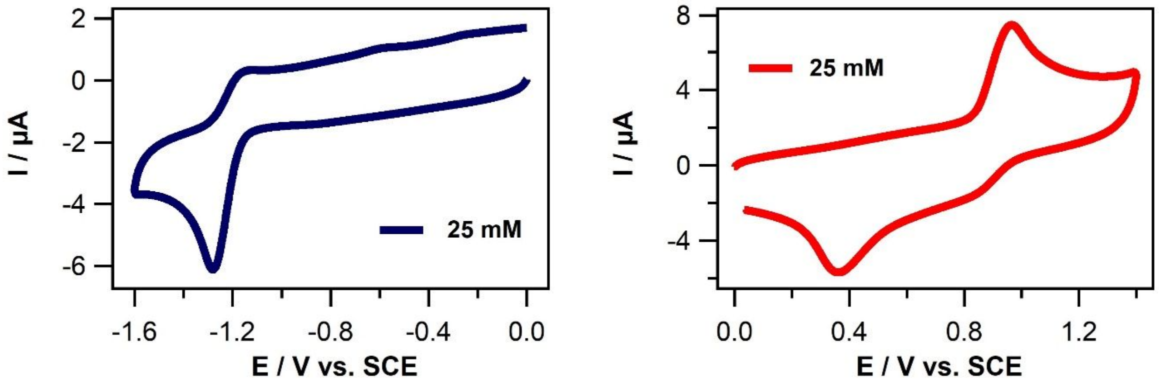


**THF**

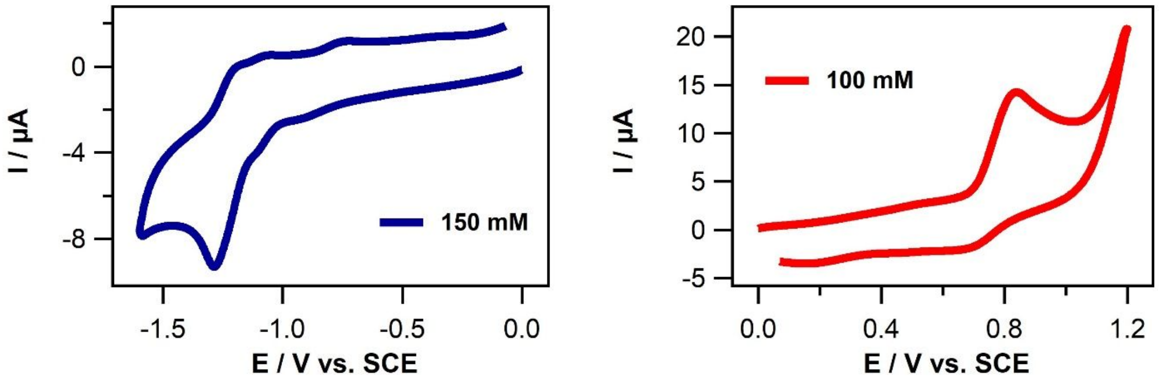


**PhCN**

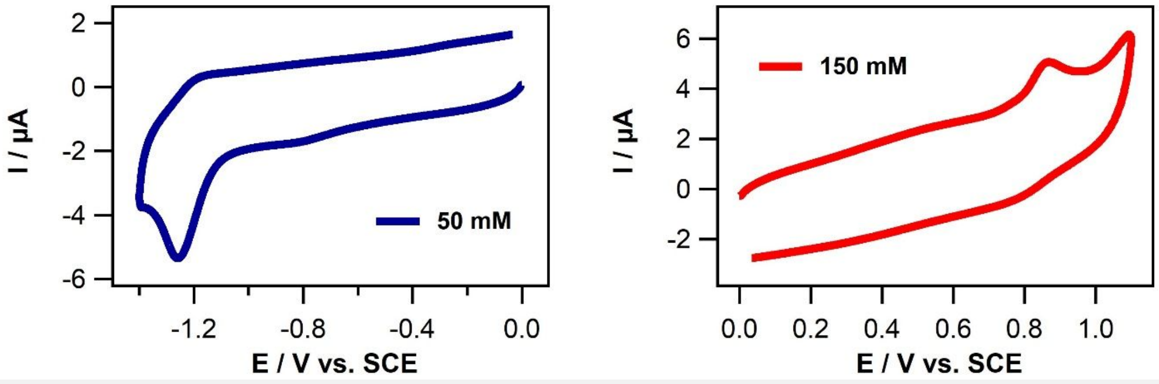


**8BgCoum**

**DCM**

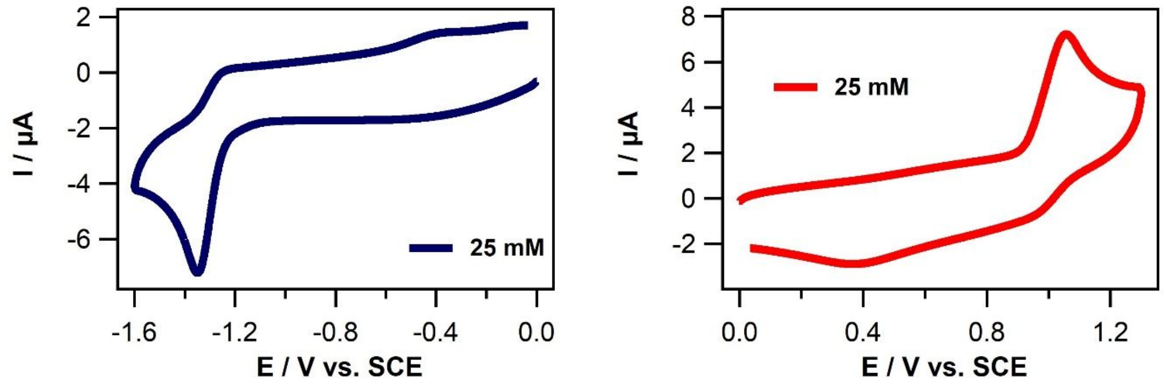


THF

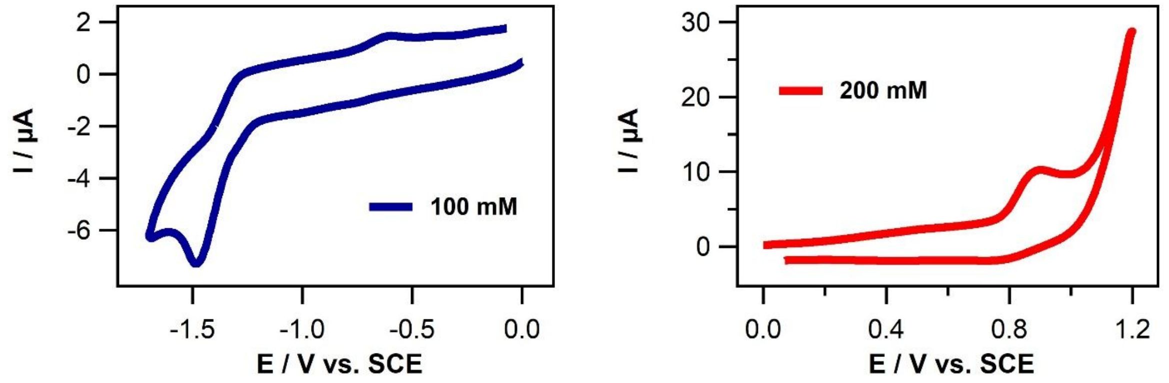


PhCN

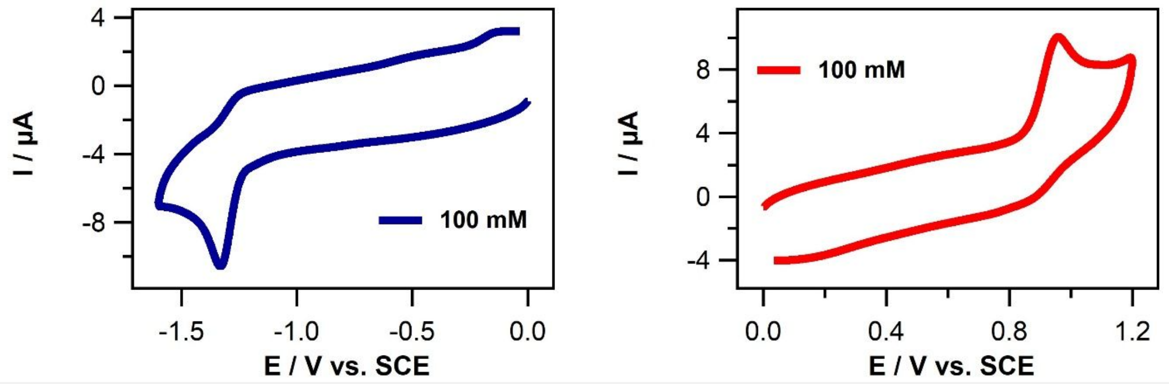


**9BgCoum**

DCM

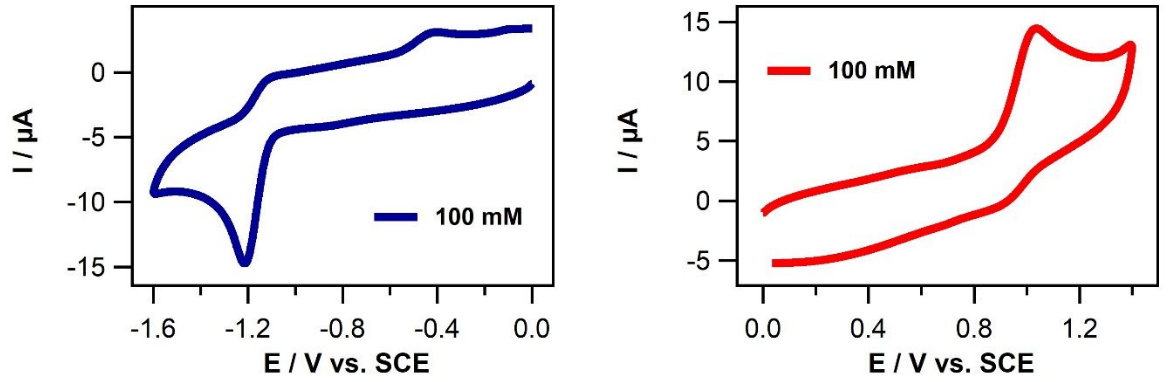

**THF**

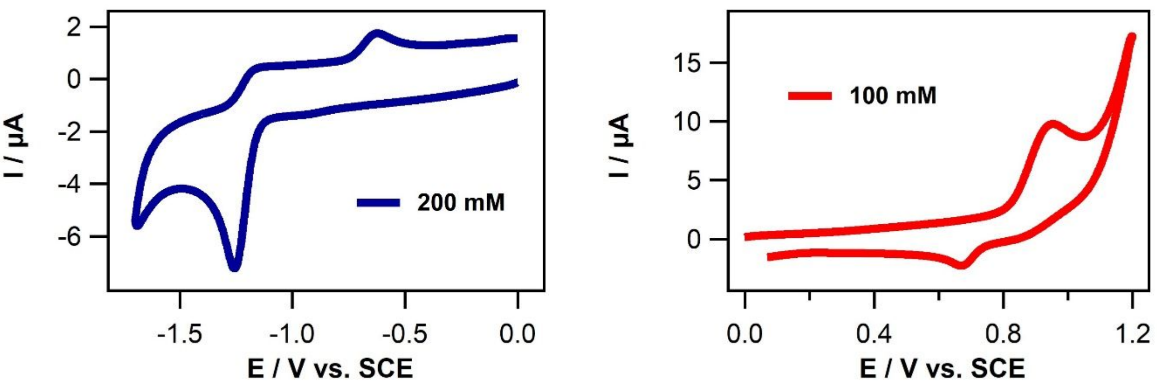


**PhCN**

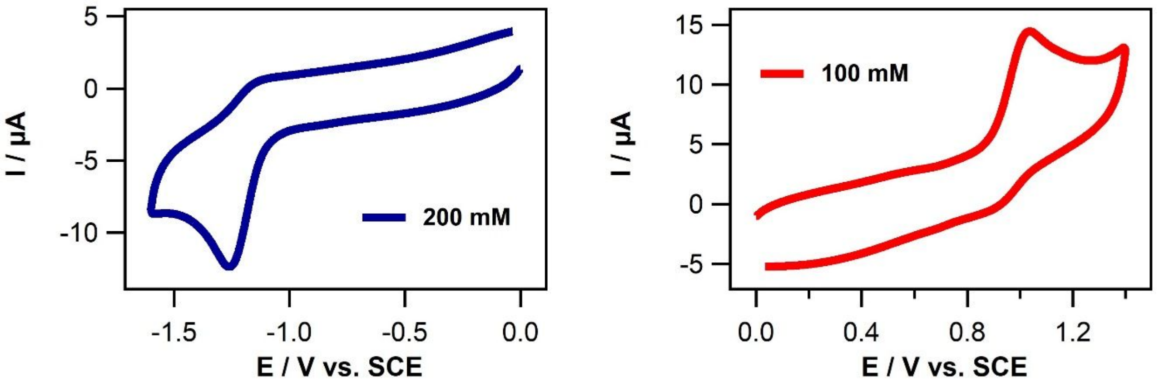


**Fig. SF8.** Cyclic voltammograms (CVs) for isomers **6**, **7**, **8** and **9-BgCoum** in DCM, THF and PhCN. Samples are dissolved in solvents in the presence of $N(n\text{-}C_4H_9)_4$ ($PF_6$) in various concentrations and purged with argon.

**Table ST3.** The half-wave reduction potentials of **6**, **7**, **8** and **9-BgCoum** in DCM, THF and PhCN corrected with ferrocene potential vs SCE.

| | DCM | | THF | | PhCN | |
|---|---|---|---|---|---|---|
| **E [V]** | **$E_I$** | **$E_{II}$** | **$E_I$** | **$E_{II}$** | **$E_I$** | **$E_{II}$** |
| **6BgCoum** | -1.18 | 1.15 | -1.52 | 0.91 | -1.27 | 0.90 |
| **7BgCoum** | -1.18 | 0.96 | -1.54 | 0.87 | -1.33 | 0.80 |
| **8BgCoum** | -1.26 | 1.06 | -1.62 | 0.96 | -1.42 | 0.96 |
| **9BgCoum** | -1.19 | 1.16 | -1.44 | 0.97 | -1.32 | 1.02 |

**Table ST4.** Determination of HOMO and LUMO levels and energy gap between them based on ferrocene parameters.

| | DCM | | | THF | | | PhCN | | |
|---|---|---|---|---|---|---|---|---|---|
| **E [eV]** | **HOMO** | **LUMO** | **Gap** | **HOMO** | **LUMO** | **Gap** | **HOMO** | **LUMO** | **Gap** |
| **6BgCoum** | - 5.95 | - 3.62 | 2.33 | - 5.71 | - 3.28 | 2.43 | - 5.70 | - 3.53 | 2.17 |
| **7BgCoum** | - 5.76 | - 3.62 | 2.14 | - 5.67 | - 3.26 | 2.41 | - 5.60 | - 3.47 | 2.13 |
| **8BgCoum** | - 5.86 | - 3.54 | 2.32 | - 5.76 | - 3.18 | 2.58 | - 5.76 | - 3.38 | 2.38 |
| **9BgCoum** | - 5.96 | - 3.61 | 2.34 | - 5.77 | - 3.36 | 2.41 | - 5.82 | - 3.48 | 2.35 |

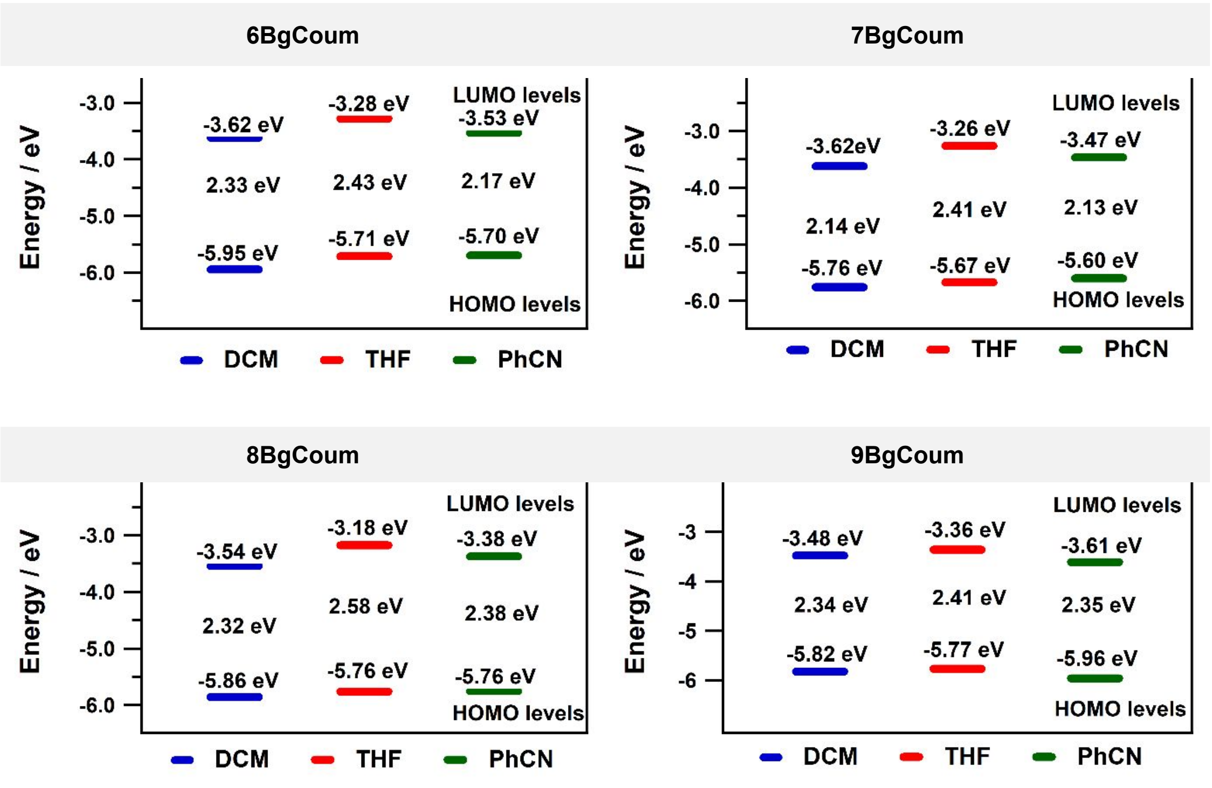


**Figure SF9**. HOMO and LUMO energy levels presented for isomers **6**, **7**, **8**, and **9BgCoum** in dichloromethane (DCM), tetrahydrofuran (THF), and benzonitrille (PhCN).

### Spectroelectrochemistry

Spectroelectrochemical analysis reveals distinct absorption peaks in the near-infrared and visible spectral regions for the reduction scans, and the visible and the ultraviolet regions for the oxidation peaks. Assigning the absorption of the radical ions to these spectral features warrants a great deal of caution, considering that the voltammograms do not reveal complete chemical reversibility. The new absorption bands that accompany the ground-state bleach during the oxidation and reduction of the coumarins can be readily associated with accumulation of degradation or dimerization products in the vicinity of the working electrode. *ΔA(t) = log(I(0)/I(t))*

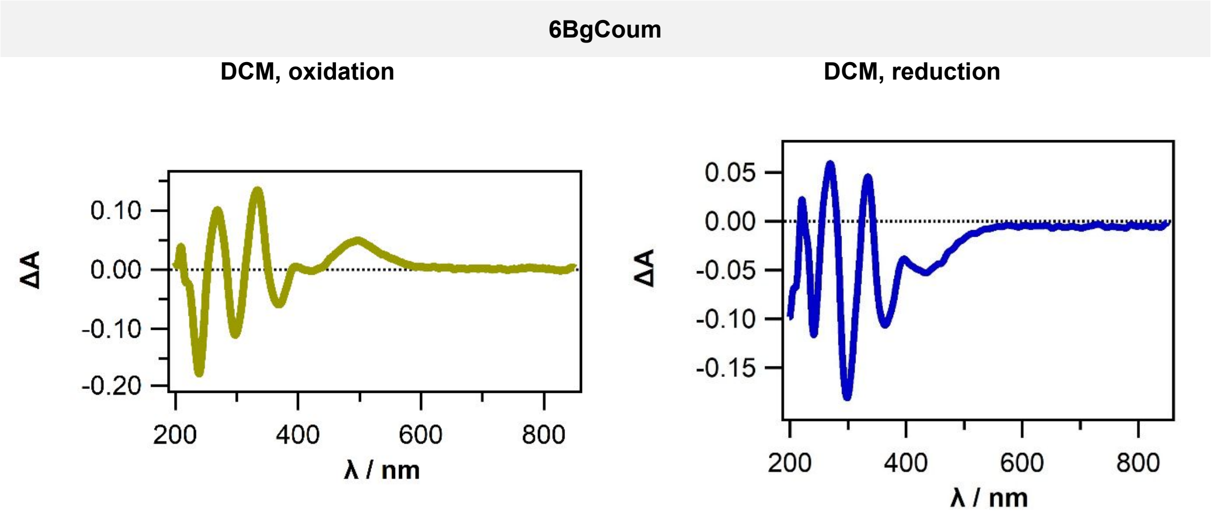


**7BgCoum**

**DCM, oxidation**

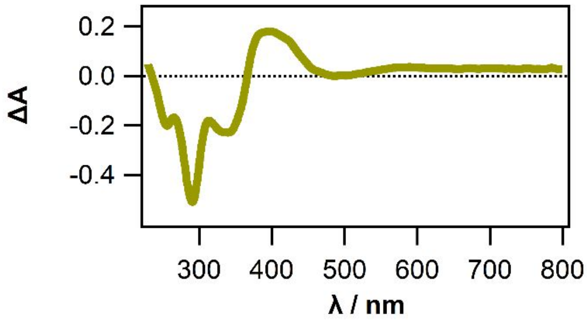


**MeCN, reduction**

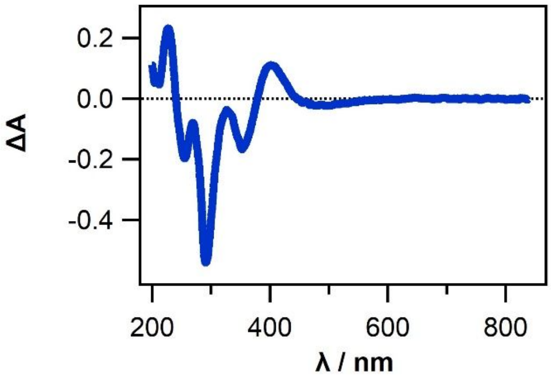


**8BgCoum**

**DCM, oxidation**

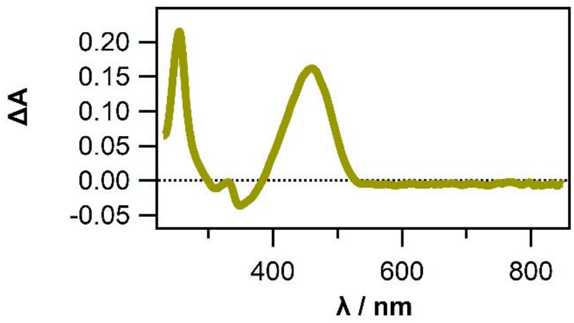


**DCM, reduction**

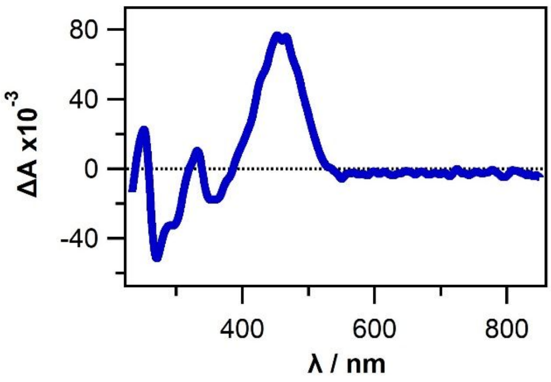


**9BgCoum**

**DCM, oxidation**

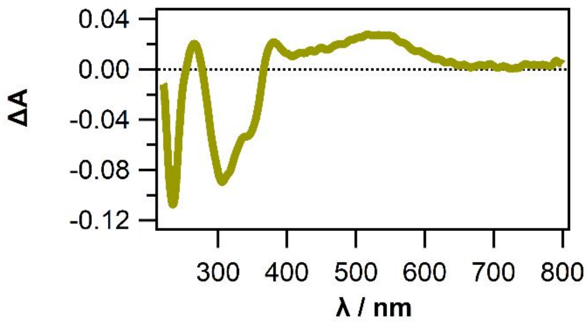


**DCM, reduction**

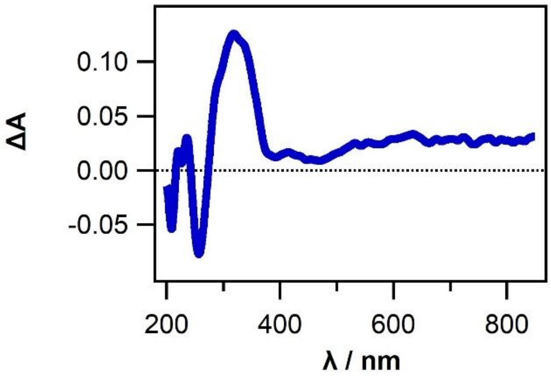


**Fig. SF10**. Absorption spectra for radicals of benzo[*g*]coumarin isomers.

## 4. Crystallography data

Single crystal X-Ray diffraction was performed on all benzo[g]coumarins isomers (**6**, **7**, **8**, **9-BgCoum**). Crystals suitable for the measurement were obtained by dissolving in ethanol and heating, then slowly cooling down (**7-BgCoum** was dissolved in methanol). There was used Bruker APEX-II CCD diffractometer with fine-focused sealed tube as radiation source.

**6-BgCoum**

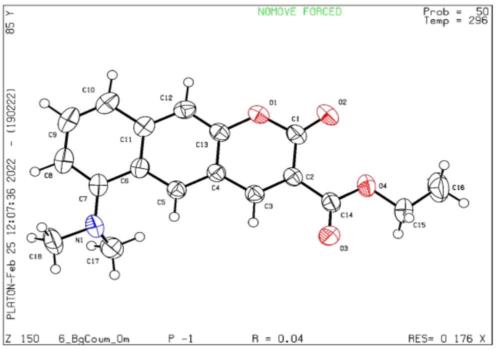


**7-BgCoum**

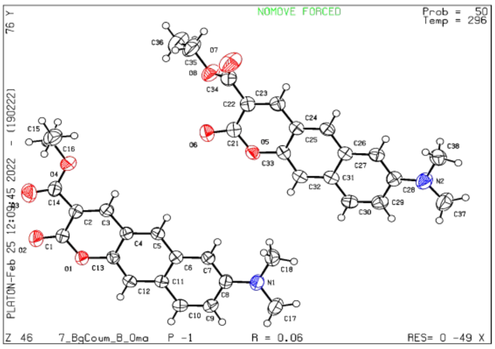


**8-BgCoum**

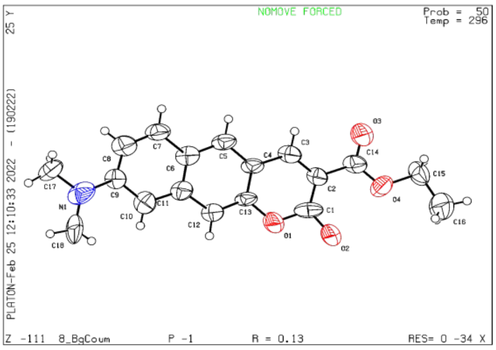


**9-BgCoum**

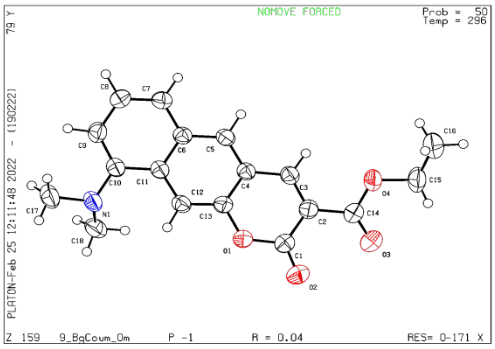


**Fig. SF11.** Anisotropic models of benzo[g]coumarins isomers (**6**, **7**, **8**, and **9BgCoum**).

**Table ST5.** Single crystal X-Ray diffraction and refinement parameters for all regioisomers of benzo[g]coumarins (empirical formula $C_{18}H_{17}NO_4$; formula weight 311.32; temperature 296(2) K).

| | | 6-BgCoum | 7-BgCoum | 8-BgCoum | 9-BgCoum |
|---|---|---|---|---|---|
| **Crystal size (mm)** | | 0.164 x 0.275 x 0.360 | 0.200 x 0.322 x 0.574 | 0.102 x 0.152 x 0.156 | 0.182 x 0.246 x 0.384 |
| **Crystal color, habit** | | orange-yellow plate | red plate | orange needle | orange cube |
| **Crystal system** | | triclinic | triclinic | triclinic | triclinic |
| **Space group** | | P -1 | P -1 | P -1 | P -1 |
| **Unit cell dimensions** | | a = 7.26920(10) Å | a = 7.5459(2) Å | a = 6.472(6) Å | a = 7.4845(2) Å |
| | | b = 8.33970(10) Å | b = 15.1113(4) Å | b = 10.543(10) Å | b = 8.2881(2) Å |
| | | c = 12.8722(2) Å | c = 15.2730(4) Å | c =11.733(11) Å | c = 12.9671(3) Å |
| | | α = 84.8540(10)° | α = 119.2274(13)° | α = 102.21(6)° | α = 96.1090(10)° |
| | | β = 84.2490(10)° | β = 90.2764(15)° | β =91.72(6)° | β = 99.8820(10)° |
| | | γ = 81.2580(10)° | γ = 92.3721(15)° | γ = 104.21(6)° | γ = 105.2100(10)° |
| **Volume** | | 764.913(19) $Å^3$ | 1517.91(7)) $Å^3$ | 755.7(12) $Å^3$ | 754.92(3) $Å^3$ |
| **Z** | | 2 | 4 | 2 | 2 |
| **Density calculated** | | 1.352 $g*cm^{-3}$ | 1.362 $g*cm^{-3}$ | 1.368 $g*cm^{-3}$ | 1.370 $g*cm^{-3}$ |
| **Reflection collected** | | 25165 | 38990 | 6894 | 26234 |
| **Independent reflections** | | 2764; $R_{int}$ = 0.0502 | 5308; $R_{int}$ = 0.0864 | 1305, $R_{int}$ = 0.1571 | 2722, $R_{int}$ = 0.053 |
| **Max. and min. transmission** | | 0.7640 | 0.8570 | 0.8860 | 0.7490 |
| | | 0.8810 | 0.6580 | 0.9230 | 0.8680 |
| **Absorption coefficient** | | 0.789 $mm^{-1}$ | 0.795 $mm^{-1}$ | 0.799 $mm^{-1}$ | 0.800 $mm^{-1}$ |
| **Extinction coefficient** | | - | 0.0011(3) | 0.0060(30) | 0.0072(10) |
| **Goodness-of-fit on $F^2$** | | 1.039 | 0.967 | 0.946 | 0.982 |
| **Final R indices** | $F^2>2\sigma(F^2)$ | $R_1$ = 0.0408, $wR_2$ = 0.1068 | $R_1$ = 0.0585, $wR_2$ = 0.1538 | $R_1$ = 0.1262, $wR_2$ = 0.2501 | $R_1$ = 0.0440, $wR_2$ = 0.1187 |
| | all data | $R_1$ = 0.0594, $wR_2$ = 0.1200 | $R_1$ = 0.0824, $wR_2$ = 0.1794 | $R_1$ = 0.3564, $wR_2$ = 0.3833 | $R_1$ = 0.0528, $wR_2$ = 0.1290 |

5. **Computational results.**

**Table ST6**. Transition energy (ΔE), oscillator strength (*f*), dipole moment (μ), leading electronic configurations, and relevant molecular orbitals of *syn,* and *anti*-conformations of **6** to **9BgCoum** computed with ADC(2)/cc-pVDZ method at the ground state MP2 equilibrium. Solvent effects were considered within the COSMO approximation using dichloromethane (DCM) as solvent.

| Compound | State | ΔE / eV | *f* | μ / Debye | Electronic Configuration |
|---|---|---|---|---|---|
| *syn*-**6BgCoum** | $S_0$ | 0 | - | 8,79 | $(78a)^2$ |
| | $^1\pi\pi^*$ | 3,3 | 0,07 | 16,44 | 0.88(78a-79a) |
| | $^1\pi\pi^*$ | 3,71 | 0,26 | 13,13 | 0.74(77a-79a) |
| | $^1n\pi^*$ | 3,91 | 0,01 | 3,37 | 0.74(73a-79a) |
| *syn*-**6BgCoum DCM** | $S_0$ | 0 | - | 10,82 | $(78a)^2$ |
| | $^1\pi\pi^*$ | 3,09 | 0,13 | 20,46 | 0.92(78a-79a) |
| | $^1\pi\pi^*$ | 3,62 | 0,25 | 14,21 | 0.79(73a-79a) |
| | $^1\pi\pi^*$ | 4,14 | 0,11 | 5,18 | 0.74(73a-79a) |
| *anti*-**6BgCoum** | $S_0$ | 0,28 | - | 8,56 | $(78a)^2$ |
| | $^1\pi\pi^*$ | 3,33 | 0,09 | 18,18 | 0.89(78a-79a) |
| | $^1\pi\pi^*$ | 3,73 | 0,23 | 10,94 | 0.77(77a-79a) |
| | $^1n\pi^*$ | 4,18 | 0,11 | 5,19 | 0.52(73a-79a) |
| *anti*-**6BgCoum DCM** | $S_0$ | 0,26 | - | 10,22 | $(78a)^2$ |
| | $^1\pi\pi^*$ | 3,11 | 0,14 | 20,9 | 0.92(78a-79a) |
| | $^1\pi\pi^*$ | 3,67 | 0,21 | 13,34 | 0.79(77a-79a) |
| | $^1\pi\pi^*$ | 4,18 | 0,45 | 15,07 | 0.68(76a-79a) |
| *syn*-**7BgCoum** | $S_0$ | - | - | 11,03 | $(78a)^2$ |
| | $^1\pi\pi^*$ | 3 | 0,05 | 21,59 | 0.94(78a-79a) |
| | $^1n\pi^*$ | 3,87 | 0,01 | 17,35 | 0.94(77a-79a) |
| | $^1\pi\pi^*$ | 4,09 | 0,56 | 14,33 | 0.92(77a-79a) |
| *syn*-**7BgCoum DCM** | $S_0$ | - | - | 8,92 | $(78a)^2$ |
| | $^1\pi\pi^*$ | 2,83 | 0,05 | 21,59 | 0.94(78a-79a) |
| | $^1n\pi^*$ | 3,87 | 0,77 | 2,69 | 0.77(74a-79a) |
| *anti*-**7BgCoum** | $S_0$ | 1,78 | - | 9,32 | $(78a)^2$ |
| | $^1\pi\pi^*$ | 3,05 | 0,05 | 19,54 | 0.94(78a-79a) |

| | | | | | |
|---|---|---|---|---|---|
| | $^1\pi\pi^*$ | 4 | 0,44 | 9,35 | 0.69(77a-79a) |
| | $^1n\pi^*$ | 4,29 | 0,04 | 6,2 | 0.44(71a-79a) |
| *anti*-**7BgCoum DCM** | $S_0$ | 0,26 | - | 11,13 | $(78a)^2$ |
| | $^1\pi\pi^*$ | 2,87 | 0,05 | 22,59 | 0.94(78a-79a) |
| | $^1\pi\pi^*$ | 3,89 | 0,69 | 17,07 | 0.92(77a-79a) |
| | $^1\pi\pi^*$ | 4,27 | 0,05 | 20,06 | 0.75(76a-79a) |
| *syn*-**8BgCoum** | $S_0$ | - | - | 9,39 | $(78a)^2$ |
| | $^1\pi\pi^*$ | 3,25 | 0,54 | 21,76 | 0.95(78a-79a) |
| | $^1\pi\pi^*$ | 3,48 | 0,08 | 12,28 | 0.88(77a-79a) |
| | $^1\pi\pi^*$ | 4,22 | 0,32 | 14,74 | 0.81(78a-80a) |
| *syn*-**8BgCoum** (ethyl) | $S_0$ | - | - | 9.37 | $(82a)^2$ |
| | $^1\pi\pi^*$ | 3.34 | 0.59 | 22.17 | 0.95(82a-83a) |
| | $^1\pi\pi^*$ | 3.68 | 0.03 | 12.44 | 0.86(81a-83a) |
| | $^1n\pi^*$ | 4.05 | 0.00 | 1.94 | 0.90(78a-83a) |
| *syn*-**8BgCoum** (benzyl) | $S_0$ | - | - | 10.23 | $(82a)^2$ |
| | $^1\pi\pi^*$ | 3.09 | 0.56 | 22.90 | 0.96(98a-99a) |
| | $^1\pi\pi^*$ | 3.47 | 0.05 | 13.35 | 0.85(97a-99a) |
| | $^1n\pi^*$ | 3.91 | 0.00 | 0.92 | 0.87(92a-99a) |
| *syn*-**8BgCoum DCM** | $S_0$ | - | - | 11,7 | $(78a)^2$ |
| | $^1\pi\pi^*$ | 2,9 | 0,63 | 24,52 | 0.96(78a-79a) |
| | $^1\pi\pi^*$ | 3,44 | 0,06 | 15,58 | 0.87(77a-79a) |
| *anti*-**8BgCoum** | $S_0$ | 0,27 | - | 10 | $(78a)^2$ |
| | $^1\pi\pi^*$ | 3,26 | 0,54 | 23,11 | 0.94(78a-79a) |
| | $^1\pi\pi^*$ | 3,56 | 0,03 | 14,18 | 0.84(77a-79a) |
| | $^1\pi\pi^*$ | 4,17 | 0,2 | 9,48 | 0.62(78a-80a) |
| *anti*-**8BgCoum** (ethyl) | $S_0$ | -0.05 | - | 6.66 | $(82a)^2$ |
| | $^1\pi\pi^*$ | 3.33 | 0.61 | 20.62 | 0.95(82a-83a) |
| | $^1\pi\pi^*$ | 3.68 | 0.03 | 10.80 | 0.85(81a-83a) |
| | $^1\pi\pi^*$ | 4.32 | 0.31 | 13.58 | 0.81(82a-84a) |
| *anti*-**8BgCoum** (benzyl) | $S_0$ | 0.27 | - | - | $(82a)^2$ |
| | $^1\pi\pi^*$ | 3.09 | 0.56 | 24.08 | 0.95(98a-99a) |
| | $^1\pi\pi^*$ | 3.54 | 0.02 | 15.01 | 0.85(97a-99a) |

| | | | | | |
|---|---|---|---|---|---|
| | $^1\pi\pi^*$ | 4.08 | 0.27 | 12.53 | 0.81(98a-100a) |
| *anti*-**8BgCoum DCM** | $S_0$ | 0,26 | - | 12,01 | $(78a)^2$ |
| | $^1\pi\pi^*$ | 2,93 | 0,6 | 26,12 | 0.95(78a-79a) |
| | $^1\pi\pi^*$ | 3,5 | 0,04 | 17,07 | 0.84(77a-79a) |
| | $^1\pi\pi^*$ | 4,.07 | 0,33 | 19 | 0.79(78a-80a) |
| *syn*-**9BgCoum** | $S_0$ | - | - | 6,94 | $(78a)^2$ |
| | $^1\pi\pi^*$ | 3,37 | 0,03 | 10,9 | 0.81(78a-79a) |
| | $^1n\pi^*$ | 3,57 | 0 | 18,14 | 0.95(76-79a) |
| | $^1n\pi^*$ | 3,88 | 0,01 | 3,98 | 0.89(73a-79a) |
| | $^1\pi\pi^*$ | 4,05 | 0,54 | 10,7 | 0.81(78a-79a) |
| *syn*-**9BgCoum DCM** | $S_0$ | - | - | 8,68 | $(78a)^2$ |
| | $^1\pi\pi^*$ | 3,3 | 0,03 | 13,41 | 0.86(78a-79a) |
| | $^1\pi\pi^*$ | 3,26 | 0 | 19,39 | 0.96(76a-79a) |
| | $^1\pi\pi^*$ | 3,91 | 0,72 | 11,93 | 0.84(77a-79a) |
| | $^1\pi\pi^*$ | 4,11 | 0 | 4,21 | 0.88(73a-79a) |
| *anti*-**9BgCoum** | $S_0$ | 0,08 | - | 8,56 | $(78a)^2$ |
| | $^1\pi\pi^*$ | 3,25 | 0,03 | 18,08 | 0.81(78a-79a) |
| | $^1\pi\pi^*$ | 3,89 | 0,22 | 12,22 | 0.95(76-79a) |
| | $^1\pi\pi^*$ | 4,12 | 0,17 | 7,61 | 0.89(73a-79a) |
| | $^1\pi\pi^*$ | 4,34 | 0,11 | 7,99 | 0.81(78a-79a) |
| *anti*-**9BgCoum DCM** | $S_0$ | 0,05 | - | 10,28 | $(78a)^2$ |
| | $^1\pi\pi^*$ | 3,07 | 0,05 | 20,82 | 0.90(78a-79a) |
| | $^1\pi\pi^*$ | 3,81 | 0,23 | 15,43 | 0.74(77-79a) |
| | $^1\pi\pi^*$ | 4,05 | 0,5 | 15,7 | 0.65(76a-79a) |
| | $^1\pi\pi^*$ | 4,34 | 0,32 | 17,53 | 0.73(78a-79a) |

**Table ST7.** Relevant molecular $\pi$ orbitals involved into the lowest excitations.

| **HOMO** | | **LUMO** | |
|---|---|---|---|
| **77a** | **78a** | **79a** | **80a** |
| *syn*-**6BgCoum** | | | |

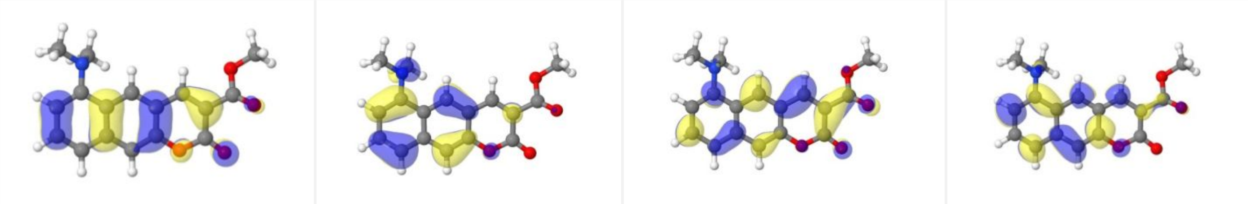

*anti*-**6BgCoum**

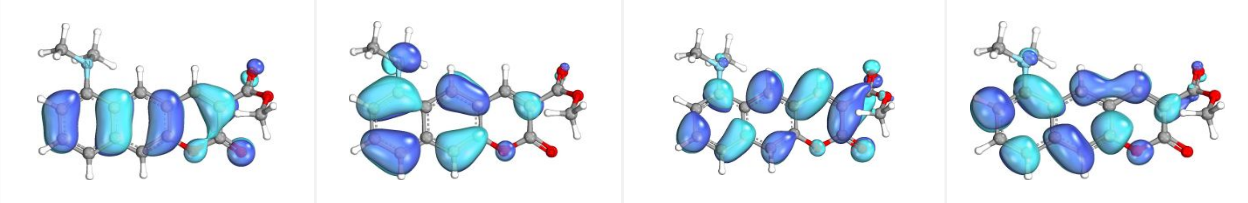

*syn*-**7BgCoum**

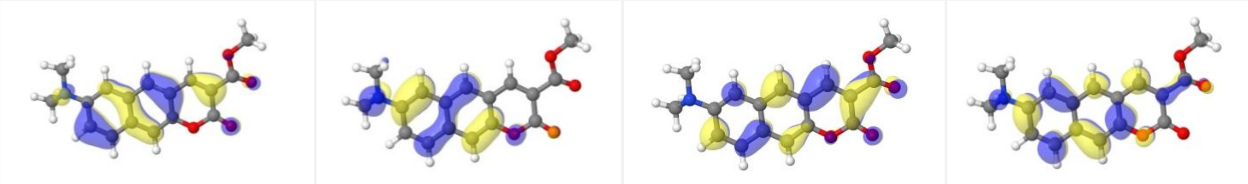

*anti*-**7BgCoum**

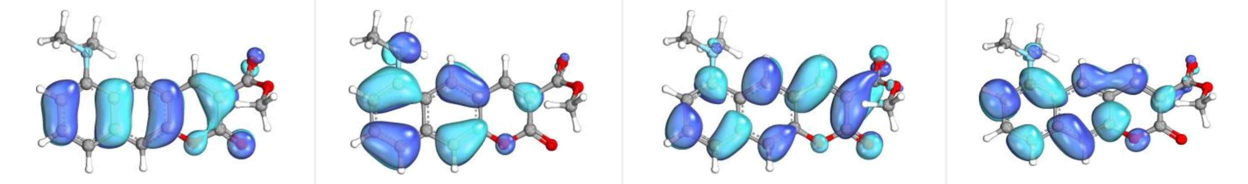

*syn*-**8BgCoum**

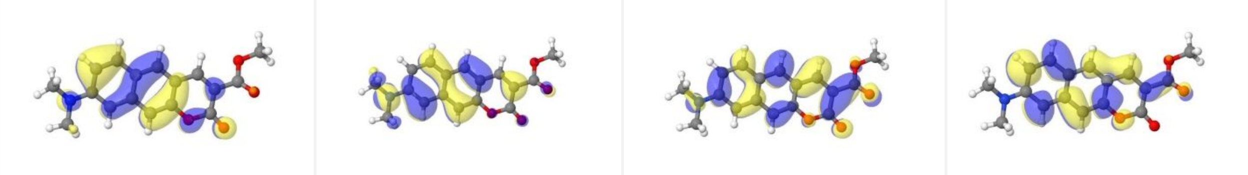

*anti*-**8BgCoum**

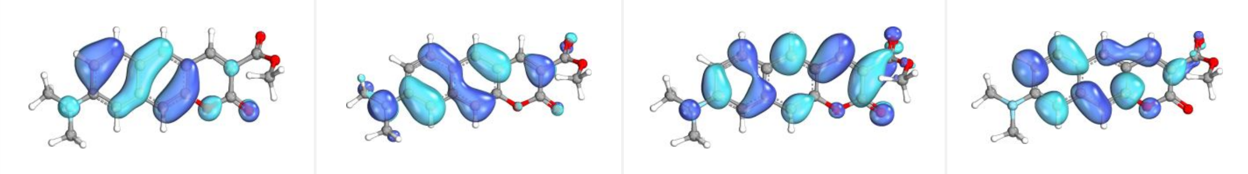

*syn*-**9BgCoum**

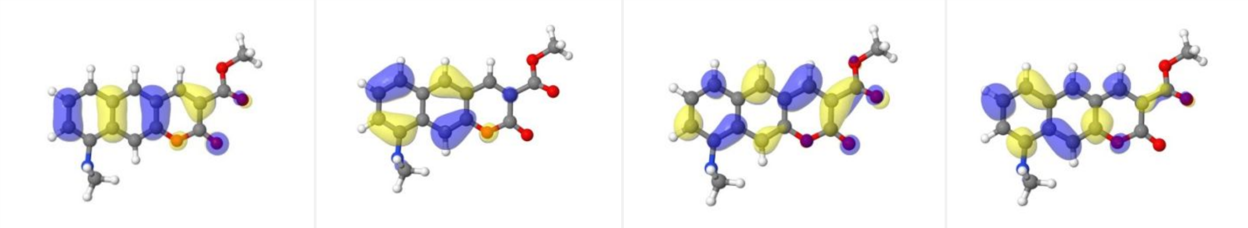

| | | | |
|---|---|---|---|
| *anti*-**9BgCoum** | | | |
| 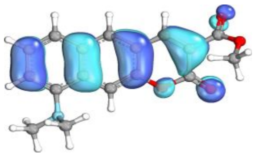 | 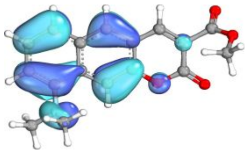 | 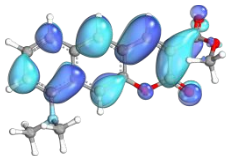 | 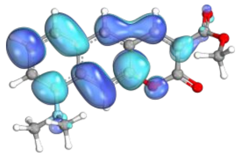 |

| State | Occupied | Virtual |
|---|---|---|
| *syn*-**6BgCoum** | | |
| **1** | 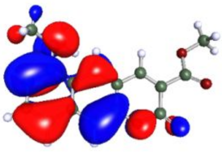 | 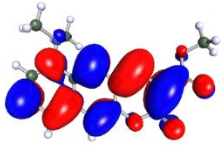 |
| **2** | 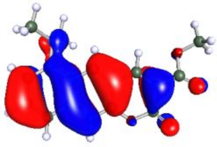 | 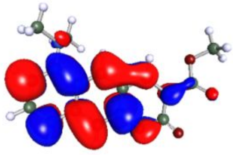 |
| *syn*-**7BgCoum** | | |
| **1** | 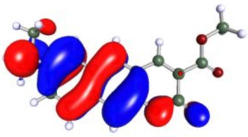 | 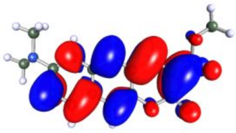 |
| **2** | 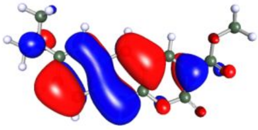 | 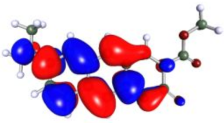 |
| **3** | 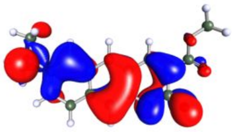 | 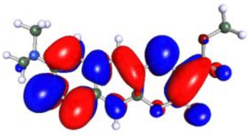 |
| *syn*-**8BgCoum** | | |
| **1** | 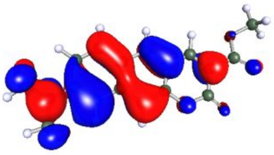 | 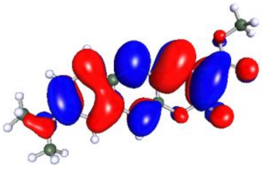 |

| | | |
|---|---|---|
| **2** | 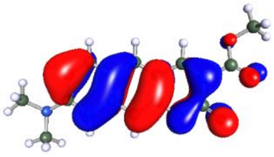 | 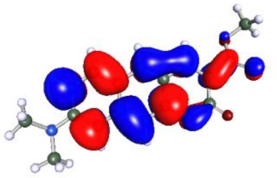 |
| *syn*-**9BgCoum** | | |
| **1** | 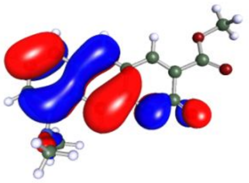 | 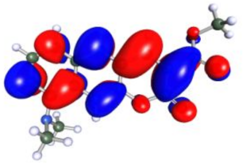 |
| **2** | 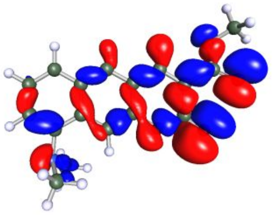 | 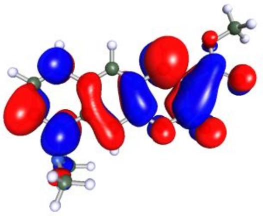 |
| **3** | 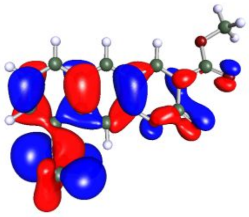 | 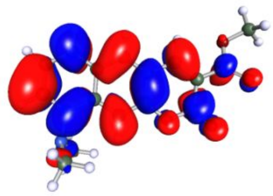 |
| **4** | 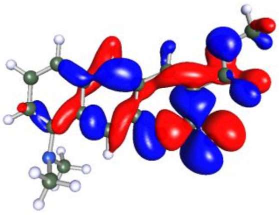 | 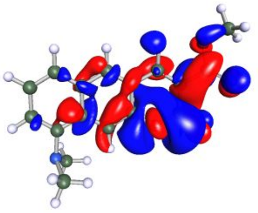 |

**Fig. SF12.** Natural transition orbitals computed for *syn* isomers of **6** to **9BgCoum** obtained at ADC(2)/cc-pVDZ level of theory using the MP2/cc-pVDZ equilibrium geometry of the ground state.

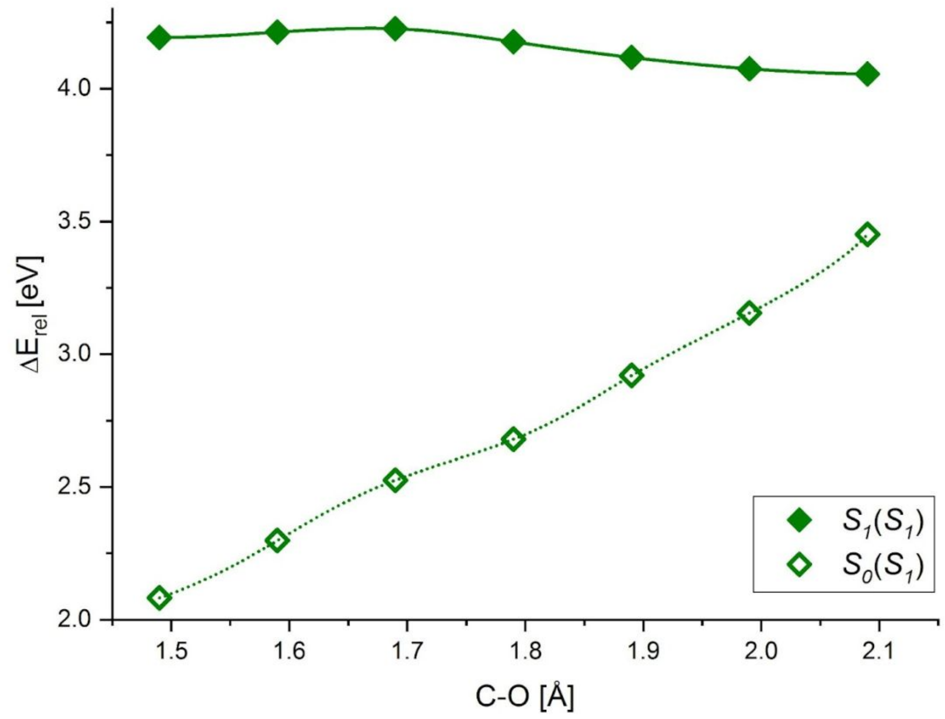


**Fig. SF13.** Molecular energy profile computed for the open ring reaction of *syn*-**6BgCoum** at MP2/ADC(2) level of theory.

**Table ST8.** Fluorescence energy ($E_f$), oscillator strength (f), dipole moment (μ) and the distance between carbon and oxygen atoms from the lacton ring (C-O) of **6** to **9BgCoum** computed with ADC(2)/cc-pVDZ method at the ground-state MP2 equilibrium. Values computed at SOS-ADC(2)/cc-pVDZ level of theory are given in parenthesis.

| | $E_f$ / eV | f | μ / Debye | C-O / Å |
|---|---|---|---|---|
| ***syn*-6-BgCoum** | 2.11 (2.46) | 0.11 (0.14) | 20.92 (18.79) | 1.49 |
| ***anti*-6-BgCoum** | 2.13 (2.48) | 0.12 (0.16) | 21.13 (19.12) | 1.46 |
| ***syn*-7-BgCoum** | 1.82 (2.13) | 0.05 (0.07) | 20.45 (18.16) | 1.58 |
| ***anti*-7-BgCoum** | 1.98 (2.29) | 0.04 (0.06) | 22.20 (19.55) | 1.50 |

| | | | | |
|---|---|---|---|---|
| ***syn*-8-BgCoum** | 0.60 (1.00) | - (0.01) | 11.18 (10.76) | 2.14 (open ring) |
| ***syn*-8-BgCoum** (ethyl) | 0.60 | - | 11.12 | 2.14 (open ring) |
| ***syn*-8-BgCoum** (benzyl) | 0.60 | - | 11.43 | 2.13 (open ring) |
| ***anti*-8-BgCoum** | 2.43 (2.75) | 0.33 (0.48) | 23.45 (21.59) | 1.48 |
| ***anti*-8-BgCoum** (ethyl) | 2.44 | 0.33 | 23.26 | 1.48 |
| ***anti*-8-BgCoum** (benzyl) | 2.37 | 0.34 | 24.14 | 1.48 |
| ***syn*-9-BgCoum** | 0.54 (2.83) | (0.04) | 7.62 (10.94) | 2.13 (open ring) |
| ***anti*-9-BgCoum** | 1.86 (2.26) | 0.03 (0.04) | 22.04 (19.45) | 1.50 |

**Table ST9.** Vertical absorption and fluorescence energy (ΔE), oscillator energy (f), and dipole moment (μ) of *syn* conformers of **6**, **7**, **8,** and **9BgCoum** surrounded by two explicit solvent molecules computed with ADC(2)/cc-pVDZ method. The conductor-like screening model (COSMO) with dielectric constant of a given solvent was used in these calculations

| | **Absorption** | | | **Emission** | | |
|---|---|---|---|---|---|---|
| | **ΔE / eV** | ***f*** | **μ / Debye** | **ΔE / eV** | ***f*** | **μ / Debye** |
| *syn*-**6BgCoum**-hex | 2.96 | 0.11 | 19.61 | 1.70 | 0.10 | 19.61 |
| *syn*-**6BgCoum**-ACN | 3.12 | 0.14 | 10.36 | 1.74 | 0.14 | 20.03 |
| *syn*-**7BgCoum**-hex | 3.11 | 0.27 | 9.19 | 0.68 | - | 15.56 |
| *syn*-**7BgCoum**-ACN | 3.64 | 0.70 | 11.33 | 0.81 | 0.01 | 27.15 |
| *syn*-**8BgCoum**-hex | 2.66 | 0.59 | 25.12 | 0.60 | - | 12.91 |

| | | | | | | |
|---|---|---|---|---|---|---|
| *syn*-**8BgCoum**-ACN | 2.68 | 0.63 | 20.87 | 1.57 | 0.38 | 34.99 |
| *syn*-**9BgCoum**-hex | 3.88 | 0.62 | 10.54 | 0.52 | - | 8.24 |
| *syn*-**9BgCoum**-ACN | 3.89 | 0.70 | 4.48 | 0.75 | 0.01 | 28.78 |

**Table ST10.** Vertical absorption and fluorescence energy (ΔE) and oscillator strength (*f*) of *syn*-**6BgCoum**, *syn*-**7BgCoum**, and *syn*-**9BgCoum** dimers computed with ADC(2)/cc-pVDZ level of theory at the ground state MP2 equilibrium.

| | | **Absorption** | | **Emission** | |
|---|---|---|---|---|---|
| | | **ΔE / eV** | ***f*** | **ΔE / eV** | ***f*** |
| *syn*-**6BgCoum** | | 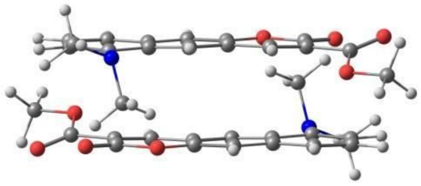 | | | |
| | $^1A_g$ | 2.84 | 0.00 | 1.78 | 0.00 |
| | $^1A_u$ | 2.87 | 0.18 | 2.02 | 0.19 |
| *syn*-**7BgCoum** | | 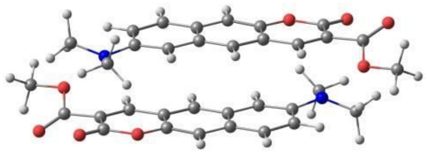 | | | |
| | $^1A_g$ | 2.13 | 0.00 | 0.99 | 0.00 |
| | $^1A_u$ | 2.64 | 0.00 | 1.82 | 0.02 |
| *syn*-**9BgCoum** | | 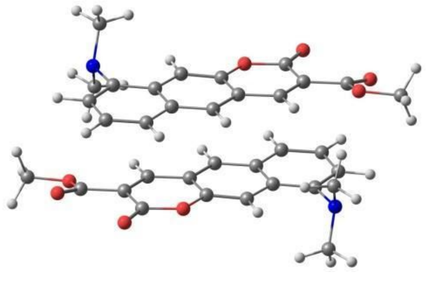 | | | |
| | $^1A_g$ | 2.99 | 0.00 | 1.69 | 0.00 |
| | $^1A_u$ | 3.25 | 0.03 | 1.93 | 0.03 |

**Table ST11.** Total (E) and zero-point vibrational energies (ZPE) given in Hartree and dipole moments (μ) computed for the respective MP2/cc-pVDZ equilibrium geometry of the ground state for the most stable conformers of each **BgCoum**.

| | **E / Hartree** | **ZPE / Hartree** | **μ / Debye** |
|---|---|---|---|

| | | | |
|---|---|---|---|
| *syn*-**6BgCoum** | -1009.64 | 0.29 | 8.79 |
| *syn*-**7BgCoum** | -1009.65 | 0.29 | 8.62 |
| *syn*-***8BgCoum*** | -1009.60 | 0.29 | 9.32 |
| *syn*-**9BgCoum** | -1009.64 | 0.29 | 6.94 |

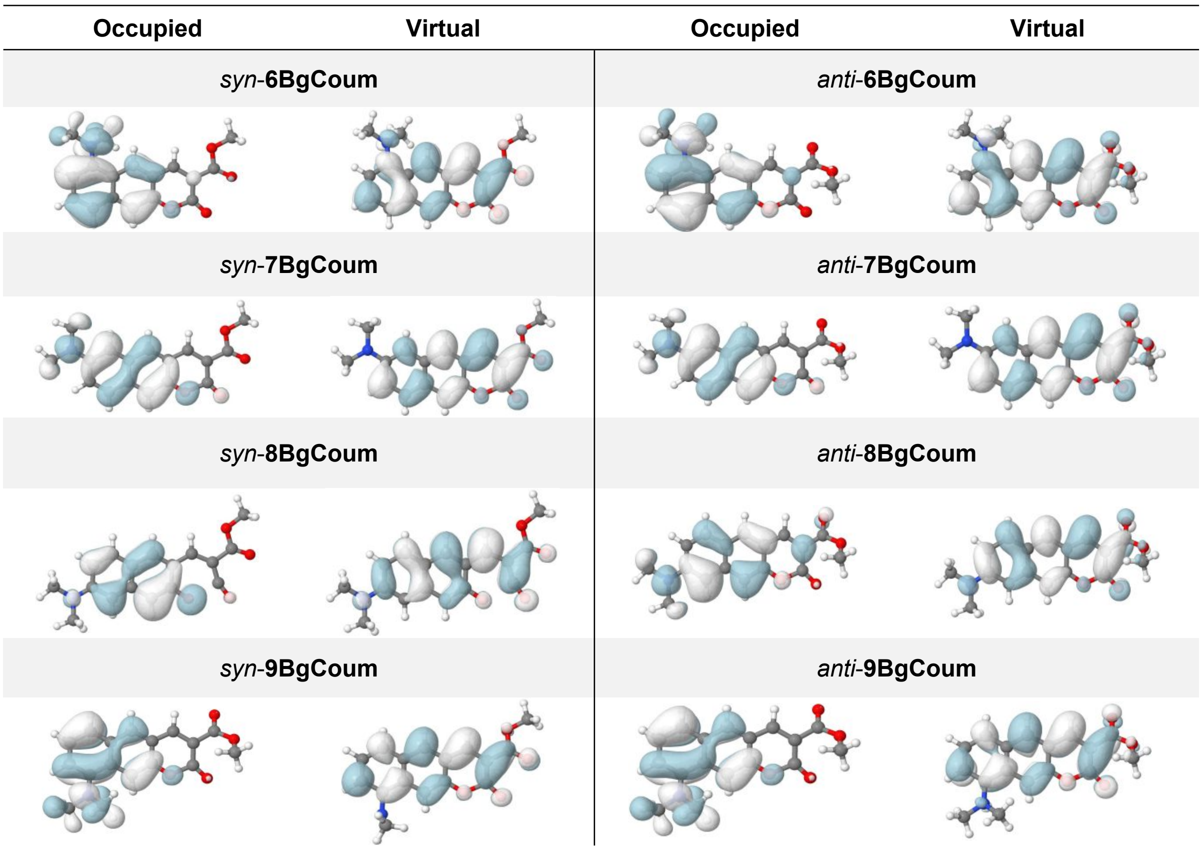


**Figure SF14.** Natural transition orbitals obtained from the S1 excited state molecular structures for *syn* and *anti* isomers of **6** to **9BgCoum** computed at ADC(2)/cc-pVDZ level of theory.

## 6. Cartesian coordinates

### Ground state – MP2/cc-pVDZ

*syn*-**6BgCoum**

| | | | |
|---|---|---|---|
| C | 0.13976299 | 1.29523305 | -2.45401014 |
| C | 0.08982834 | 1.26520992 | -1.01333669 |
| C | 0.12328545 | 0.04440417 | -0.29479196 |
| C | 0.05971152 | 2.50614934 | -0.27447059 |
| C | -0.01912962 | 2.47323606 | 1.14419234 |
| C | -0.03495121 | 1.25917917 | 1.81721270 |
| C | 0.05740488 | 0.02933822 | 1.10359731 |
| C | 0.24455530 | 2.52575583 | -3.10111324 |
| C | 0.13898536 | 3.74169482 | -0.98455861 |
| H | -0.05673565 | 3.40221971 | 1.72263598 |
| O | -0.12503735 | 1.29168440 | 3.18639147 |
| C | 0.11571320 | -1.18621610 | 1.88006043 |
| C | 0.05329157 | -1.14906765 | 3.24437182 |
| C | -0.12655918 | 0.13382406 | 3.97072162 |
| H | 0.22230831 | -2.14750787 | 1.36735679 |
| C | 0.25405768 | 3.74462405 | -2.36624545 |
| H | 0.23847006 | -0.89529882 | -0.84439477 |
| H | 0.29943438 | 2.56645030 | -4.19259308 |
| H | 0.12456121 | 4.68181050 | -0.42210258 |
| O | -0.28701852 | 0.26234724 | 5.16033842 |
| C | 0.19511926 | -2.40147217 | 4.05741205 |
| O | 0.58268032 | -2.46190459 | 5.20515002 |
| O | -0.14319373 | -3.48706844 | 3.31048594 |
| C | 0.04924934 | -4.73351276 | 4.00133440 |
| H | 1.10336677 | -4.86028778 | 4.29200960 |
| H | -0.57447317 | -4.77458424 | 4.90695180 |
| H | -0.25210704 | -5.51191080 | 3.28932239 |
| H | 0.33098119 | 4.69414550 | -2.90679432 |
| N | 0.09616558 | 0.04230380 | -3.13397086 |
| C | 0.68334279 | 0.04639016 | -4.46895712 |
| H | 1.69523752 | 0.47769765 | -4.42727311 |
| H | 0.75678266 | -0.99560017 | -4.82097965 |
| H | 0.08291001 | 0.61159544 | -5.21503131 |
| C | -1.26409742 | -0.50961451 | -3.17682215 |
| H | -1.92580036 | 0.07469612 | -3.85280637 |
| H | -1.22307546 | -1.55056428 | -3.54007430 |
| H | -1.70502702 | -0.50537939 | -2.16921878 |

*syn*-**7BgCoum**

| | | | |
|---|---|---|---|
| C | 0.03140118 | 0.73772307 | -2.12975413 |
| C | 0.11994409 | 0.69108982 | -0.70527991 |
| C | 0.05836863 | -0.53411494 | 0.00441197 |
| C | 0.24225883 | 1.91688921 | 0.04920885 |
| C | 0.31842886 | 1.87463472 | 1.46533330 |
| C | 0.26022548 | 0.65461026 | 2.12847028 |
| C | 0.12918813 | -0.56635798 | 1.40408763 |
| C | 0.09141925 | 1.95150301 | -2.82111471 |
| C | 0.30833995 | 3.14385201 | -0.67820816 |
| H | 0.41925873 | 2.79494334 | 2.05003189 |
| O | 0.34233531 | 0.67187288 | 3.49755473 |
| C | 0.05488184 | -1.78597229 | 2.16962376 |
| C | 0.11770509 | -1.76410631 | 3.53533539 |
| C | 0.31007884 | -0.49124035 | 4.27426973 |
| H | -0.06532991 | -2.74212891 | 1.65022151 |
| C | 0.23919701 | 3.16080291 | -2.05998256 |
| H | -0.04330910 | -1.47830180 | -0.54534425 |
| O | 0.45528987 | -0.37214831 | 5.46710085 |
| C | -0.03902383 | -3.02362404 | 4.33365723 |
| O | -0.41008016 | -3.09346312 | 5.48648775 |
| O | 0.26608047 | -4.10667272 | 3.56761903 |
| C | 0.06144884 | -5.35734503 | 4.24643008 |
| H | 0.69886245 | -5.42149366 | 5.14114137 |
| H | -0.98993876 | -5.46827101 | 4.55316158 |
| H | 0.33643808 | -6.13261701 | 3.52039257 |
| H | 0.34524861 | 4.11301715 | -2.58819900 |
| H | -0.08589482 | -0.20966796 | -2.66264520 |
| H | 0.43054972 | 4.08337637 | -0.12707427 |
| N | 0.08122130 | 2.04189315 | -4.22860517 |
| C | 0.07374949 | 0.77508364 | -4.94368010 |
| H | 0.18509257 | 0.98178567 | -6.01951347 |
| H | 0.92476639 | 0.15673860 | -4.61944094 |
| H | -0.86391367 | 0.19508730 | -4.79745148 |
| C | -0.90842314 | 2.96590449 | -4.78666650 |
| H | -0.68810746 | 3.12439411 | -5.85471657 |
| H | -1.94359209 | 2.57211654 | -4.69481553 |
| H | -0.86416614 | 3.94020716 | -4.28204753 |

*syn*-**8BgCoum**

| | | | |
|---|---|---|---|
| C | -0.17828786 | -0.02242421 | -2.53452960 |
| C | -0.12022128 | -0.07157834 | -1.11044144 |
| C | -0.01620096 | -1.28573989 | -0.39391001 |
| C | -0.16853188 | 1.17548569 | -0.38920948 |
| C | -0.10434525 | 1.15229003 | 1.03050747 |
| C | -0.00447206 | -0.05400549 | 1.70721971 |
| C | 0.04147959 | -1.29244012 | 1.00407094 |
| C | -0.27848017 | 1.18099633 | -3.20669786 |
| C | -0.26322814 | 2.40045867 | -1.10482400 |
| H | -0.13745996 | 2.08077540 | 1.60843391 |
| O | 0.04258508 | -0.00866327 | 3.07819433 |
| C | 0.16486626 | -2.49576656 | 1.78522937 |
| C | 0.22927643 | -2.44552907 | 3.14884324 |
| C | 0.12844480 | -1.15506328 | 3.87257999 |
| H | 0.22018504 | -3.46294203 | 1.27750954 |
| C | -0.33711065 | 2.43369917 | -2.50390645 |
| H | -0.13867571 | -0.95858557 | -3.10159441 |
| H | 0.02218633 | -2.23742103 | -0.93667494 |
| H | -0.32442557 | 1.16353267 | -4.29667314 |
| H | -0.27944064 | 3.32173265 | -0.51972809 |

*syn*-**9BgCoum**

| | | | |
|---|---|---|---|
| C | 0.01811788 | 0.07627029 | -3.38816318 |
| C | 0.01241129 | 0.03601462 | -1.96226036 |
| C | 0.01225339 | -1.19828802 | -1.26688187 |
| C | 0.01074910 | 1.27184248 | -1.20605453 |
| C | 0.00328393 | 1.20376503 | 0.21337074 |
| C | -0.00115281 | -0.02380584 | 0.86047993 |
| C | 0.00420471 | -1.24520287 | 0.12993236 |
| C | 0.02071253 | 1.29303904 | -4.05097701 |
| C | 0.01344483 | 2.52045699 | -1.92113904 |
| H | -0.00087685 | 2.10309741 | 0.82787232 |
| O | -0.01718753 | -0.00972408 | 2.23104992 |
| C | 0.02139303 | -2.47339806 | 0.88441395 |
| C | 0.02470887 | -2.45535212 | 2.24960925 |
| C | -0.03808573 | -1.17715983 | 3.00041458 |
| H | 0.04492353 | -3.43071438 | 0.35602970 |
| C | 0.01864219 | 2.50639434 | -3.31405506 |
| H | 0.01796114 | -2.13697772 | -1.83292099 |
| H | 0.02485818 | 1.32313721 | -5.14466951 |
| O | -0.12084944 | -1.05677288 | 4.19803917 |
| C | 0.12517643 | -3.72709251 | 3.03615282 |

| | | | | | | | |
|---|---|---|---|---|---|---|---|
| O | 0.09841039 | -1.00847932 | 5.07042155 | O | 0.55242346 | -3.83027179 | 4.16601593 |
| C | 0.43568591 | -3.69006745 | 3.95567779 | O | -0.30426073 | -4.77675174 | 2.28689570 |
| O | 0.89822673 | -3.74464112 | 5.07523631 | C | -0.16201968 | -6.04382041 | 2.94945516 |
| O | 0.05599597 | -4.78310531 | 3.23952642 | H | 0.89164447 | -6.23863128 | 3.19712836 |
| C | 0.30024190 | -6.02215417 | 3.92298605 | H | -0.75206052 | -6.06379719 | 3.87686309 |
| H | 1.37070397 | -6.14180633 | 4.14554361 | H | -0.53611258 | -6.78947577 | 2.23848818 |
| H | -0.26206005 | -6.06181479 | 4.86697921 | H | 0.02101328 | 3.47349108 | -3.82459020 |
| H | -0.04158764 | -6.80691977 | 3.23814619 | H | 0.01975784 | -0.86698663 | -3.94414161 |
| N | -0.49253508 | 3.62572345 | -3.21287347 | N | 0.01180458 | 3.80853484 | -1.29113761 |
| C | 0.00457830 | 3.67117636 | -4.58246351 | C | -1.21446234 | 4.15840102 | -0.58333293 |
| H | 1.08709603 | 3.44485262 | -4.65873815 | H | -2.07969753 | 3.90043339 | -1.21278229 |
| H | -0.54523983 | 2.97012609 | -5.22668320 | H | -1.34297159 | 3.66385288 | 0.40187965 |
| H | -0.17081372 | 4.67995995 | -4.98119025 | H | -1.22586132 | 5.24824644 | -0.41412218 |
| C | -0.33063492 | 4.85982384 | -2.46283270 | C | 1.23142687 | 4.15554223 | -0.57054482 |
| H | -0.51854852 | 5.70870889 | -3.13438510 | H | 1.34814914 | 3.66143859 | 0.41633298 |
| H | -1.06899446 | 4.90870471 | -1.64829509 | H | 2.10257221 | 3.89477960 | -1.19061485 |
| H | 0.68133166 | 4.97110058 | -2.02145473 | H | 1.24396576 | 5.24548563 | -0.40203576 |
| | *anti*-**6BgCoum** | | | | *anti*-**7BgCoum** | | |
| C | -0.03855811 | 0.84082275 | -2.61247801 | C | -0.32717301 | 0.34102236 | -2.25722478 |
| C | -0.12375906 | 0.85313767 | -1.17293237 | C | -0.33312568 | 0.34124924 | -0.82946870 |
| C | -0.07895771 | -0.34510316 | -0.41792932 | C | -0.41650724 | -0.86290009 | -0.08648188 |
| C | -0.20277846 | 2.11501959 | -0.47403201 | C | -0.21986465 | 1.58860851 | -0.10957187 |
| C | -0.31815107 | 2.12186363 | 0.94239814 | C | -0.21594355 | 1.59071588 | 1.30907594 |
| C | -0.31911032 | 0.92754278 | 1.64868900 | C | -0.30294831 | 0.39082520 | 2.00354050 |
| C | -0.17829105 | -0.32224779 | 0.97839767 | C | -0.40354699 | -0.85407444 | 1.31521767 |
| C | 0.05451369 | 2.05401626 | -3.29351106 | C | -0.24398626 | 1.53330206 | -2.98316267 |
| C | -0.13507937 | 3.33062555 | -1.21877272 | C | -0.14237407 | 2.79423958 | -0.87072085 |
| H | -0.39651794 | 3.06574771 | 1.49190993 | H | -0.14265657 | 2.52839847 | 1.86976937 |
| O | -0.44909010 | 1.00196287 | 3.01822461 | O | -0.30294677 | 0.45590434 | 3.37878179 |
| C | -0.12316654 | -1.51278958 | 1.79426890 | C | -0.47898816 | -2.05131213 | 2.11676130 |
| C | -0.18424400 | -1.42284641 | 3.15510315 | C | -0.41431349 | -1.98083918 | 3.47959858 |
| C | -0.42232302 | -0.12607524 | 3.82294959 | C | -0.38569659 | -0.67716988 | 4.17304548 |
| H | -0.02436904 | -2.49465576 | 1.31660836 | H | -0.57893915 | -3.02816442 | 1.62888637 |
| C | 0.01598563 | 3.29433716 | -2.59643486 | C | -0.15715765 | 2.76820524 | -2.25382115 |
| H | 0.07045384 | -1.29713538 | -0.93704856 | H | -0.49710496 | -1.82281024 | -0.61172792 |
| H | 0.13636787 | 2.06350655 | -4.38398701 | H | -0.08593064 | 3.75376101 | -0.34399005 |
| H | -0.18758132 | 4.28652421 | -0.68603169 | O | -0.44163187 | -0.53183071 | 5.37703855 |
| O | -0.60483928 | 0.01311400 | 5.01467633 | C | -0.55901329 | -3.25291632 | 4.28057122 |
| C | -0.19134886 | -2.69417110 | 3.97099332 | O | -1.45263681 | -4.03916510 | 4.04313194 |
| O | -0.93473514 | -3.61034202 | 3.68732675 | O | 0.34562648 | -3.52671279 | 5.24508191 |
| O | 0.67136417 | -2.81446324 | 5.00249298 | C | 1.53492554 | -2.73545020 | 5.37459670 |
| C | 1.70525666 | -1.84092654 | 5.20435421 | H | 1.83656752 | -2.29719343 | 4.40912959 |
| H | 2.00808459 | -1.36932461 | 4.25505671 | H | 1.38433695 | -1.93799044 | 6.11454028 |
| H | 1.37164142 | -1.07110086 | 5.91316793 | H | 2.31861764 | -3.42742216 | 5.71322952 |
| H | 2.56026597 | -2.39434079 | 5.61688054 | H | -0.14924222 | 3.71301582 | -2.80527077 |
| H | 0.08375399 | 4.22859112 | -3.16408329 | N | -0.30917164 | 1.58746036 | -4.38994280 |
| N | -0.03519539 | -0.43191241 | -3.25410773 | C | 0.76393191 | 2.34328167 | -5.03910912 |
| C | 0.58161017 | -0.45330259 | -4.57564962 | H | 0.89660891 | 3.32441027 | -4.56418237 |
| H | 1.58126580 | 0.00485019 | -4.52555940 | H | 1.73446365 | 1.80328915 | -5.00155557 |
| H | 0.68891239 | -1.50329115 | -4.89322606 | H | 0.49681523 | 2.50898224 | -6.09524490 |
| H | -0.01615736 | 0.07325022 | -5.35142762 | C | -0.53741283 | 0.31841315 | -5.06357608 |
| C | -1.37935116 | -1.02221506 | -3.30887965 | H | -0.68546014 | 0.51562013 | -6.13668247 |
| H | -2.03992930 | -0.47808346 | -4.01842371 | H | 0.31038513 | -0.39319432 | -4.95565447 |
| H | -1.30190989 | -2.07268424 | -3.63661380 | H | -1.44883632 | -0.15652181 | -4.66944824 |
| H | -1.84403266 | -0.99790084 | -2.31236962 | H | -0.38967004 | -0.62503705 | -2.76516005 |
| | *anti*-**8BgCoum** | | | | *anti*-**9BgCoum** | | |
| C | 1.64765115 | -2.12821548 | 0.08645963 | C | -0.25800275 | -0.53696424 | -3.35975599 |
| C | 0.59272809 | -1.17504789 | -0.04226098 | C | -0.25161321 | -0.51637235 | -1.93147812 |
| C | -0.77046521 | -1.55109816 | -0.09428578 | C | -0.16151541 | -1.70903059 | -1.17112005 |
| C | 0.94935162 | 0.22109785 | -0.11504299 | C | -0.36412650 | 0.74568670 | -1.23619377 |
| C | -0.08433717 | 1.18824295 | -0.25531175 | C | -0.45071436 | 0.75907205 | 0.18115325 |
| C | -1.41183663 | 0.78848321 | -0.30723034 | C | -0.36935115 | -0.43153971 | 0.89106155 |

| | | | | | | | |
|---|---|---|---|---|---|---|---|
| C | -1.77825668 | -0.58772946 | -0.22676443 | C | -0.21381766 | -1.68448848 | 0.22807223 |
| C | 2.97120943 | -1.73010467 | 0.13718960 | C | -0.37694324 | 0.64806494 | -4.06846604 |
| C | 2.31940163 | 0.60399622 | -0.07056903 | C | -0.42106921 | 1.96651659 | -1.99874706 |
| H | 0.15038972 | 2.25580899 | -0.32263867 | H | -0.60506389 | 1.70243243 | 0.71068231 |
| O | -2.36158691 | 1.77754022 | -0.45323929 | O | -0.47009374 | -0.35308560 | 2.26201238 |
| C | -3.18401564 | -0.90706280 | -0.27779646 | C | -0.13577908 | -2.86944638 | 1.04843051 |
| C | -4.11948708 | 0.08493851 | -0.35847825 | C | -0.17328174 | -2.77332470 | 2.41030084 |
| C | -3.71731079 | 1.49748174 | -0.51496963 | C | -0.40672760 | -1.47438406 | 3.07453391 |
| H | -3.50563702 | -1.95466442 | -0.24118048 | H | -0.03230643 | -3.85358604 | 0.57626547 |
| C | 3.34502230 | -0.34205108 | 0.07192548 | C | -0.43883371 | 1.89925525 | -3.39278769 |
| H | 1.39893349 | -3.19437862 | 0.14069582 | H | -0.06181265 | -2.67346247 | -1.68445314 |
| H | -1.04624374 | -2.61132504 | -0.03525030 | H | -0.39311430 | 0.63021314 | -5.16348051 |
| H | 3.74018797 | -2.49848964 | 0.24135929 | O | -0.55769640 | -1.32695252 | 4.26978815 |
| H | 2.54486972 | 1.67005131 | -0.14893679 | C | -0.17214332 | -4.04326762 | 3.22807756 |
| O | -4.48541985 | 2.41843136 | -0.70481375 | O | -0.91572572 | -4.96147077 | 2.95100351 |
| C | -5.57597920 | -0.29647248 | -0.47102820 | O | 0.69927965 | -4.16212023 | 4.25257768 |
| O | -5.93941705 | -1.14255276 | -1.26164264 | C | 1.74004416 | -3.19354577 | 4.44167863 |
| O | -6.47273415 | 0.29132883 | 0.35071835 | H | 2.03639008 | -2.72623922 | 3.48822160 |
| C | -6.03225964 | 1.10316203 | 1.44782045 | H | 1.41744107 | -2.41982914 | 5.15121768 |
| H | -6.76455282 | 0.94282675 | 2.25146188 | H | 2.59602866 | -3.75052029 | 4.84745050 |
| H | -5.03563178 | 0.79532537 | 1.80446499 | H | -0.50785523 | 2.81833492 | -3.98133537 |
| H | -6.01209454 | 2.16170095 | 1.15575895 | N | -0.45821953 | 3.18602842 | -1.26278559 |
| N | 4.68839666 | 0.02542742 | 0.18173810 | C | -1.02702765 | 4.32249410 | -1.97883277 |
| C | 5.01086439 | 1.41569842 | -0.10079899 | H | -1.99740484 | 4.03680140 | -2.41267626 |
| H | 4.76092637 | 1.71800985 | -1.13986725 | H | -1.18705404 | 5.14170632 | -1.25884854 |
| H | 4.46933861 | 2.07901640 | 0.59195997 | H | -0.37359937 | 4.71527681 | -2.78847827 |
| H | 6.08736153 | 1.56862694 | 0.06317922 | C | 0.86295294 | 3.52846568 | -0.72198727 |
| C | 5.69241749 | -0.93504665 | -0.26588145 | H | 1.58726783 | 3.77616507 | -1.52798293 |
| H | 6.68624660 | -0.48217451 | -0.13606768 | H | 0.76640141 | 4.39925351 | -0.05225193 |
| H | 5.67041034 | -1.84680864 | 0.34959184 | H | 1.26184162 | 2.68286488 | -0.14100133 |
| H | 5.57155881 | -1.22397302 | -1.33026837 | H | -0.18675463 | -1.49900199 | -3.87986512 |
| *syn*-**8BgCoum** (ethyl) | | | | *anti*-**8BgCoum** (ethyl) | | | |
| C | -0.23315233 | 0.53698187 | -2.66755602 | C | -0.46709549 | -0.11065939 | -3.27091118 |
| C | -0.13746132 | 0.49881716 | -1.24789251 | C | -0.28802387 | -0.01987226 | -1.86160125 |
| C | -0.08172023 | -0.70322178 | -0.52464404 | C | -0.25868700 | -1.14917218 | -1.02847416 |
| C | -0.09428259 | 1.73232894 | -0.55295113 | C | -0.13130652 | 1.26897343 | -1.29473274 |
| C | 0.00716484 | 1.72217230 | 0.86170450 | C | 0.05496970 | 1.38713880 | 0.10644271 |
| C | 0.05877307 | 0.53765630 | 1.52931624 | C | 0.07810381 | 0.27007924 | 0.88288903 |
| C | 0.01476430 | -0.70190892 | 0.84692649 | C | -0.07920281 | -1.02332022 | 0.32922823 |
| C | -0.28534716 | 1.70741846 | -3.34542468 | C | -0.49222253 | 0.99099296 | -4.05679540 |
| C | -0.14543201 | 2.94834963 | -1.27922090 | C | -0.15573940 | 2.41123407 | -2.13337742 |
| H | 0.04302463 | 2.64548189 | 1.42044353 | H | 0.17823143 | 2.35530713 | 0.56843287 |
| O | 0.15357361 | 0.56075124 | 2.87570545 | O | 0.25625369 | 0.41445283 | 2.21403553 |
| C | 0.07724808 | -1.89825212 | 1.64847311 | C | -0.03920642 | -2.13532464 | 1.24224334 |
| C | 0.17328819 | -1.85143460 | 2.98754091 | C | 0.13914998 | -1.96760031 | 2.56347931 |
| C | 0.21171236 | -0.53979076 | 3.67830080 | C | 0.30169187 | -0.60410640 | 3.11761771 |
| H | 0.04691459 | -2.85596100 | 1.15209903 | H | -0.15535427 | -3.14037176 | 0.86262477 |
| C | -0.25124472 | 2.96892269 | -2.65370810 | C | -0.34321045 | 2.30773974 | -3.49541755 |
| H | -0.26439011 | -0.39623333 | -3.21517957 | H | -0.58547008 | -1.08786255 | -3.72114041 |
| H | -0.11493058 | -1.64638636 | -1.05513347 | H | -0.37881627 | -2.13466171 | -1.45995524 |
| H | -0.36014531 | 1.68179728 | -4.41954724 | H | -0.63325083 | 0.86838443 | -5.11747399 |
| H | -0.09860795 | 3.86337001 | -0.71307864 | H | -0.02169472 | 3.37111302 | -1.66364341 |
| O | 0.28462078 | -0.35880867 | 4.83708760 | O | 0.46449886 | -0.31216686 | 4.24644002 |
| C | 0.24402285 | -3.10522465 | 3.79527006 | C | 0.16084336 | -3.20660442 | 3.40035306 |
| O | 0.35956583 | -3.16720355 | 4.97022801 | O | 0.02647499 | -4.29009241 | 2.91923973 |
| O | 0.16317879 | -4.18534205 | 3.01881929 | O | 0.33681674 | -2.99786443 | 4.68029745 |
| C | 0.22415004 | -5.45219046 | 3.66903527 | C | 0.36757149 | -4.13673387 | 5.53720565 |
| H | 1.16251482 | -5.52522740 | 4.21659788 | H | -0.57118684 | -4.68004135 | 5.43772109 |
| H | -0.58795029 | -5.51948861 | 4.39120972 | H | 1.17111656 | -4.79881961 | 5.21736650 |
| N | -0.34097999 | 4.14404826 | -3.37238677 | N | -0.40801376 | 3.41249677 | -4.32036056 |
| C | -0.04063137 | 4.14223395 | -4.79143342 | C | -0.19411731 | 3.25691552 | -5.74660350 |
| H | 0.96499125 | 3.76750633 | -5.01301557 | H | 0.77297474 | 2.79979263 | -5.98431934 |
| H | -0.76161850 | 3.54766679 | -5.35236448 | H | -0.98069955 | 2.65874408 | -6.20638009 |
| H | -0.10970942 | 5.16067844 | -5.16415713 | H | -0.22636128 | 4.23772661 | -6.21340603 |

| | | | | | | | |
|---|---|---|---|---|---|---|---|
| C | -0.18585163 | 5.40090490 | -2.67236982 | C | -0.13926912 | 4.71794417 | -3.75715722 |
| H | -0.37115175 | 6.21816454 | -3.36485969 | H | -0.31978406 | 5.47572724 | -4.51543837 |
| H | -0.91551270 | 5.48510105 | -1.86756996 | H | -0.81248325 | 4.92355373 | -2.92547825 |
| H | 0.81561893 | 5.53096687 | -2.24438351 | H | 0.89224460 | 4.82531961 | -3.40004690 |
| C | 0.11428329 | -6.51830795 | 2.60356340 | C | 0.58004784 | -3.63261976 | 6.94553784 |
| H | 0.93349346 | -6.43815809 | 1.88689072 | H | -0.22956930 | -2.96501514 | 7.24238576 |
| H | 0.15664428 | -7.50661296 | 3.06535041 | H | 0.61007457 | -4.47640871 | 7.63801355 |
| H | -0.82942802 | -6.43156565 | 2.06231424 | H | 1.51970094 | -3.08431802 | 7.02115885 |

| *syn*-**8BgCoum** (benzyl) | | | | *anti*-**8BgCoum** (benzyl) | | | |
|---|---|---|---|---|---|---|---|
| C | -0.23315233 | 0.53698187 | -2.66755602 | C | -0.46709549 | -0.11065939 | -3.27091118 |
| C | -0.13746132 | 0.49881716 | -1.24789251 | C | -0.28802387 | -0.01987226 | -1.86160125 |
| C | -0.08172023 | -0.70322178 | -0.52464404 | C | -0.25868700 | -1.14917218 | -1.02847416 |
| C | -0.09428259 | 1.73232894 | -0.55295113 | C | -0.13130652 | 1.26897343 | -1.29473274 |
| C | 0.00716484 | 1.72217230 | 0.86170450 | C | 0.05496970 | 1.38713880 | 0.10644271 |
| C | 0.05877307 | 0.53765630 | 1.52931624 | C | 0.07810381 | 0.27007924 | 0.88288903 |
| C | 0.01476430 | -0.70190892 | 0.84692649 | C | -0.07920281 | -1.02332022 | 0.32922823 |
| C | -0.28534716 | 1.70741846 | -3.34542468 | C | -0.49222253 | 0.99099296 | -4.05679540 |
| C | -0.14543201 | 2.94834963 | -1.27922090 | C | -0.15573940 | 2.41123407 | -2.13337742 |
| H | 0.04302463 | 2.64548189 | 1.42044353 | H | 0.17823143 | 2.35530713 | 0.56843287 |
| O | 0.15357361 | 0.56075124 | 2.87570545 | O | 0.25625369 | 0.41445283 | 2.21403553 |
| C | 0.07724808 | -1.89825212 | 1.64847311 | C | -0.03920642 | -2.13532464 | 1.24224334 |
| C | 0.17328819 | -1.85143460 | 2.98754091 | C | 0.13914998 | -1.96760031 | 2.56347931 |
| C | 0.21171236 | -0.53979076 | 3.67830080 | C | 0.30169187 | -0.60410640 | 3.11761771 |
| H | 0.04691459 | -2.85596100 | 1.15209903 | H | -0.15535427 | -3.14037176 | 0.86262477 |
| C | -0.25124472 | 2.96892269 | -2.65370810 | C | -0.34321045 | 2.30773974 | -3.49541755 |
| H | -0.26439011 | -0.39623333 | -3.21517957 | H | -0.58547008 | -1.08786255 | -3.72114041 |
| H | -0.11493058 | -1.64638636 | -1.05513347 | H | -0.37881627 | -2.13466171 | -1.45995524 |
| H | -0.36014531 | 1.68179728 | -4.41954724 | H | -0.63325083 | 0.86838443 | -5.11747399 |
| H | -0.09860795 | 3.86337001 | -0.71307864 | H | -0.02169472 | 3.37111302 | -1.66364341 |
| O | 0.28462078 | -0.35880867 | 4.83708760 | O | 0.46449886 | -0.31216686 | 4.24644002 |
| C | 0.24402285 | -3.10522465 | 3.79527006 | C | 0.16084336 | -3.20660442 | 3.40035306 |
| O | 0.35956583 | -3.16720355 | 4.97022801 | O | 0.02647499 | -4.29009241 | 2.91923973 |
| O | 0.16317879 | -4.18534205 | 3.01881929 | O | 0.33681674 | -2.99786443 | 4.68029745 |
| C | 0.22415004 | -5.45219046 | 3.66903527 | C | 0.36757149 | -4.13673387 | 5.53720565 |
| H | 1.16251482 | -5.52522740 | 4.21659788 | H | -0.57118684 | -4.68004135 | 5.43772109 |
| H | -0.58795029 | -5.51948861 | 4.39120972 | H | 1.17111656 | -4.79881961 | 5.21736650 |
| N | -0.34097999 | 4.14404826 | -3.37238677 | N | -0.40801376 | 3.41249677 | -4.32036056 |
| C | -0.04063137 | 4.14223395 | -4.79143342 | C | -0.19411731 | 3.25691552 | -5.74660350 |
| H | 0.96499125 | 3.76750633 | -5.01301557 | H | 0.77297474 | 2.79979263 | -5.98431934 |
| H | -0.76161850 | 3.54766679 | -5.35236448 | H | -0.98069955 | 2.65874408 | -6.20638009 |
| H | -0.10970942 | 5.16067844 | -5.16415713 | H | -0.22636128 | 4.23772661 | -6.21340603 |
| C | -0.18585163 | 5.40090490 | -2.67236982 | C | -0.13926912 | 4.71794417 | -3.75715722 |
| H | -0.37115175 | 6.21816454 | -3.36485969 | H | -0.31978406 | 5.47572724 | -4.51543837 |
| H | -0.91551270 | 5.48510105 | -1.86756996 | H | -0.81248325 | 4.92355373 | -2.92547825 |
| H | 0.81561893 | 5.53096687 | -2.24438351 | H | 0.89224460 | 4.82531961 | -3.40004690 |
| C | 0.11428329 | -6.51830795 | 2.60356340 | C | 0.58004784 | -3.63261976 | 6.94553784 |
| H | 0.93349346 | -6.43815809 | 1.88689072 | H | -0.22956930 | -2.96501514 | 7.24238576 |
| H | 0.15664428 | -7.50661296 | 3.06535041 | H | 0.61007457 | -4.47640871 | 7.63801355 |
| H | -0.82942802 | -6.43156565 | 2.06231424 | H | 1.51970094 | -3.08431802 | 7.02115885 |

**Excited state – ADC(2)/cc-pVDZ**

| *syn*-**6BgCoum - $S_1$** | | | | *anti*-**6BgCoum - $S_1$** | | | |
|---|---|---|---|---|---|---|---|
| C | 0.13324702 | 1.36516820 | -2.47339331 | C | -0.07125654 | 0.92878474 | -2.68369865 |
| C | 0.10135054 | 1.27027011 | -1.04393095 | C | -0.09550082 | 0.86788004 | -1.24937029 |
| C | 0.12195341 | 0.03845821 | -0.32508775 | C | -0.02535413 | -0.34245373 | -0.50165327 |
| C | 0.08731743 | 2.50655196 | -0.30560618 | C | -0.14853971 | 2.12153052 | -0.54395582 |
| C | 0.02348773 | 2.46752644 | 1.11195274 | C | -0.21104252 | 2.11889399 | 0.87459779 |
| C | -0.02354456 | 1.24242958 | 1.80040555 | C | -0.21779846 | 0.91232535 | 1.59094955 |
| C | 0.04743060 | -0.00745707 | 1.09544636 | C | -0.09716017 | -0.35064137 | 0.92118942 |

| | | | | | | | |
|---|---|---|---|---|---|---|---|
| C | 0.37379815 | 2.63418559 | -3.11186231 | C | 0.12184141 | 2.18464589 | -3.35655999 |
| C | 0.25557479 | 3.75159717 | -0.98116136 | C | -0.02602067 | 3.35356637 | -1.25587401 |
| H | 0.01711326 | 3.39244365 | 1.69902075 | H | -0.25499445 | 3.05866478 | 1.43576666 |
| O | -0.09935079 | 1.29902666 | 3.13342004 | O | -0.31560899 | 1.00010209 | 2.92850174 |
| C | 0.05330363 | -1.18768578 | 1.87081183 | C | -0.04799742 | -1.50477982 | 1.73247388 |
| C | -0.01252416 | -1.15739091 | 3.28493235 | C | -0.07274385 | -1.42532460 | 3.14416576 |
| C | -0.10523889 | 0.08886865 | 4.00759694 | C | -0.30731595 | -0.17110386 | 3.80696914 |
| H | 0.11994997 | -2.15966252 | 1.37105376 | H | 0.02661268 | -2.49807593 | 1.27364883 |
| C | 0.42411126 | 3.81993109 | -2.37626878 | C | 0.13490785 | 3.39198126 | -2.65114059 |
| H | 0.26875576 | -0.90796926 | -0.85579471 | H | 0.15307441 | -1.29766269 | -1.00567777 |
| H | 0.42305073 | 2.67967755 | -4.20326917 | H | 0.16209168 | 2.20338213 | -4.44919244 |
| H | 0.26490494 | 4.67037222 | -0.38277044 | H | -0.04655151 | 4.28771784 | -0.68200585 |
| O | -0.18410068 | 0.35555009 | 5.18061492 | O | -0.50998853 | 0.09476786 | 4.97567140 |
| C | 0.00137631 | -2.39910943 | 4.08002734 | C | -0.10365370 | -2.72021317 | 3.88053038 |
| O | -0.05204869 | -2.51002510 | 5.29493961 | O | -0.56502823 | -3.73693028 | 3.38354102 |
| O | 0.08746892 | -3.50219288 | 3.25510690 | O | 0.39931137 | -2.79213937 | 5.14704730 |
| C | 0.10858293 | -4.73542751 | 3.97464927 | C | 1.43876319 | -1.90020253 | 5.55904091 |
| H | 0.97292728 | -4.78579969 | 4.65585421 | H | 1.88905120 | -1.38219545 | 4.69699187 |
| H | -0.80760734 | -4.86502555 | 4.57257709 | H | 1.04760906 | -1.15594359 | 6.26517911 |
| H | 0.17941698 | -5.52349993 | 3.21190992 | H | 2.20630181 | -2.52250871 | 6.04651298 |
| H | 0.57257000 | 4.77938182 | -2.88003573 | H | 0.24680440 | 4.34234719 | -3.18073465 |
| N | -0.08038444 | 0.26780430 | -3.27931151 | N | -0.25456113 | -0.19593011 | -3.45930055 |
| C | 0.37729754 | 0.23613115 | -4.66341287 | C | 0.20299915 | -0.25369701 | -4.84329630 |
| H | 1.33732459 | 0.76157733 | -4.75789031 | H | 1.14242309 | 0.30417063 | -4.95538414 |
| H | 0.52448297 | -0.81788820 | -4.94722273 | H | 0.38699528 | -1.31017497 | -5.09418041 |
| H | -0.36401144 | 0.68193093 | -5.35437149 | H | -0.55670503 | 0.14354570 | -5.54326258 |
| C | -1.09128890 | -0.74702273 | -2.96430461 | C | -1.22128687 | -1.24240244 | -3.10734556 |
| H | -1.82724403 | -0.76109932 | -3.78940301 | H | -1.95990540 | -1.30742225 | -3.92721616 |
| H | -0.63605865 | -1.74861058 | -2.88145876 | H | -0.72302954 | -2.22100393 | -3.00269094 |
| H | -1.59339421 | -0.49301624 | -2.02376358 | H | -1.72674292 | -0.98350057 | -2.17023776 |
| | *syn*-**7BgCoum - *S*₁** | | | | *anti*-**7BgCoum- *S*₁** | | |
| C | -0.09080110 | 0.70728478 | -2.08787699 | C | -0.32717301 | 0.34102236 | -2.25722478 |
| C | -0.02235779 | 0.67943215 | -0.69274262 | C | -0.33312568 | 0.34124924 | -0.82946870 |
| C | 0.00807053 | -0.56852778 | 0.02896157 | C | -0.41650724 | -0.86290009 | -0.08648188 |
| C | 0.02840442 | 1.93240680 | 0.03101591 | C | -0.21986465 | 1.58860851 | -0.10957187 |
| C | 0.09622092 | 1.88489577 | 1.44472326 | C | -0.21594355 | 1.59071588 | 1.30907594 |
| C | 0.12154425 | 0.65619250 | 2.13140480 | C | -0.30294831 | 0.39082520 | 2.00354050 |
| C | 0.07312315 | -0.62236609 | 1.42452959 | C | -0.40354699 | -0.85407444 | 1.31521767 |
| C | -0.11896337 | 1.94250632 | -2.81799499 | C | -0.24398626 | 1.53330206 | -2.98316267 |
| C | -0.00241817 | 3.14584453 | -0.70261199 | C | -0.14237407 | 2.79423958 | -0.87072085 |
| H | 0.13579187 | 2.80281668 | 2.04201123 | H | -0.14265657 | 2.52839847 | 1.86976937 |
| O | 0.18894098 | 0.70152842 | 3.45041402 | O | -0.30294677 | 0.45590434 | 3.37878179 |
| C | 0.10089214 | -1.81565791 | 2.19286935 | C | -0.47898816 | -2.05131213 | 2.11676130 |
| C | 0.16957936 | -1.80530522 | 3.61207800 | C | -0.41431349 | -1.98083918 | 3.47959858 |
| C | 0.21771311 | -0.59043789 | 4.36486474 | C | -0.38569659 | -0.67716988 | 4.17304548 |
| H | 0.06622763 | -2.78088008 | 1.67857528 | H | -0.57893915 | -3.02816442 | 1.62888637 |
| C | -0.07031590 | 3.17451110 | -2.09643253 | C | -0.15715765 | 2.76820524 | -2.25382115 |
| H | -0.03084908 | -1.50709323 | -0.54020505 | H | -0.49710496 | -1.82281024 | -0.61172792 |
| O | 0.27540187 | -0.28207744 | 5.51987826 | H | -0.08593064 | 3.75376101 | -0.34399005 |
| C | 0.19496827 | -3.05538637 | 4.39627833 | O | -0.44163187 | -0.53183071 | 5.37703855 |
| O | 0.25066469 | -3.16847173 | 5.61135734 | C | -0.55901329 | -3.25291632 | 4.28057122 |
| O | 0.14648620 | -4.14887426 | 3.56283656 | O | -1.45263681 | -4.03916510 | 4.04313194 |
| C | 0.16809692 | -5.39140521 | 4.26857911 | O | 0.34562648 | -3.52671279 | 5.24508191 |
| H | 1.08835950 | -5.49444406 | 4.86509593 | C | 1.53492554 | -2.73545020 | 5.37459670 |
| H | -0.69469490 | -5.47852191 | 4.94778564 | H | 1.83656752 | -2.29719343 | 4.40912959 |
| H | 0.12533037 | -6.17167945 | 3.49622174 | H | 1.38433695 | -1.93799044 | 6.11454028 |
| H | -0.09744484 | 4.13476136 | -2.61465123 | H | 2.31861764 | -3.42742216 | 5.71322952 |
| H | -0.12314476 | -0.24711898 | -2.62137680 | H | -0.14924222 | 3.71301582 | -2.80527077 |
| H | 0.03173523 | 4.09294451 | -0.15044892 | N | -0.30917164 | 1.58746036 | -4.38994280 |
| N | -0.18282991 | 1.92290712 | -4.18463781 | C | 0.76393191 | 2.34328167 | -5.03910912 |
| C | -0.28800411 | 0.66047038 | -4.91186045 | H | 0.89660891 | 3.32441027 | -4.56418237 |
| H | -0.37302939 | 0.87928810 | -5.98388431 | H | 1.73446365 | 1.80328915 | -5.00155557 |
| H | 0.60512279 | 0.03292196 | -4.74951873 | H | 0.49681523 | 2.50898224 | -6.09524490 |
| H | -1.18051452 | 0.09449842 | -4.59535535 | C | -0.53741283 | 0.31841315 | -5.06357608 |

| | | | | | | | |
|---|---|---|---|---|---|---|---|
| C | -0.20226708 | 3.17827531 | -4.93033387 | H | -0.68546014 | 0.51562013 | -6.13668247 |
| H | -0.19542842 | 2.95215793 | -6.00430450 | H | 0.31038513 | -0.39319432 | -4.95565447 |
| H | -1.10584923 | 3.76690644 | -4.69410526 | H | -1.44883632 | -0.15652181 | -4.66944824 |
| H | 0.68623833 | 3.78569699 | -4.69113924 | H | -0.38967004 | -0.62503705 | -2.76516005 |
| *syn*-**8BgCoum** - ***S*$_1$** | | | | *anti*-**8BgCoum** - ***S*$_1$** | | | |
| C | -0.13317209 | 0.00421672 | -2.40780430 | C | 1.71788645 | -2.15659950 | -0.11218850 |
| C | -0.10517199 | 0.03354181 | -1.01680778 | C | 0.64613558 | -1.22609416 | -0.14940989 |
| C | -0.00035258 | -1.17363674 | -0.19365337 | C | -0.72436932 | -1.60914350 | -0.15782354 |
| C | -0.17574852 | 1.30144178 | -0.37930034 | C | 0.97838563 | 0.17144707 | -0.17679571 |
| C | -0.13690054 | 1.32862423 | 1.07301865 | C | -0.06174108 | 1.14186759 | -0.21726179 |
| C | -0.03667659 | 0.15404293 | 1.84209896 | C | -1.41433255 | 0.73712117 | -0.23255790 |
| C | 0.03367413 | -1.14758004 | 1.18421773 | C | -1.77676041 | -0.64523797 | -0.19840310 |
| C | -0.22868232 | 1.19489594 | -3.17329262 | C | 3.06764709 | -1.76723657 | -0.10357766 |
| C | -0.27389599 | 2.47571105 | -1.12145960 | C | 2.33560011 | 0.57072722 | -0.16875869 |
| H | -0.18632695 | 2.28042512 | 1.61405889 | H | 0.15997773 | 2.21477424 | -0.24516051 |
| O | -0.00884476 | 0.26252537 | 3.13581087 | O | -2.32187467 | 1.71790499 | -0.28793825 |
| C | 0.13674488 | -2.36890746 | 1.95053400 | C | -3.16227774 | -0.93941908 | -0.21105427 |
| C | 0.18415051 | -2.51110393 | 3.36957042 | C | -4.14299051 | 0.08669834 | -0.20379025 |
| C | 0.14292373 | -1.50963748 | 4.31891255 | C | -3.78089479 | 1.46593142 | -0.33030632 |
| H | 0.18411952 | -3.30327975 | 1.38450270 | H | -3.50516701 | -1.98038366 | -0.21757139 |
| C | -0.31654634 | 2.45084878 | -2.55840463 | C | 3.40422162 | -0.39356483 | -0.13204495 |
| H | -0.06943063 | -0.95542144 | -2.93346662 | H | 1.48055642 | -3.22735001 | -0.09084084 |
| H | 0.05194908 | -2.13915336 | -0.71444826 | H | -0.98608988 | -2.67493648 | -0.13600691 |
| H | -0.23273925 | 1.10390037 | -4.26066625 | H | 3.84024821 | -2.53817333 | -0.07557569 |
| H | -0.32152413 | 3.42437083 | -0.58172871 | H | 2.55550437 | 1.64217592 | -0.19192368 |
| O | 0.13992621 | -1.02946374 | 5.39040492 | O | -4.43786304 | 2.47815069 | -0.47568977 |
| C | 0.29056753 | -3.84882155 | 4.01604567 | C | -5.56227417 | -0.35408515 | -0.31264146 |
| O | 0.33678884 | -4.05679668 | 5.21788394 | O | -5.87350108 | -1.39696279 | -0.86795301 |
| O | 0.33132464 | -4.84094918 | 3.08651110 | O | -6.55458739 | 0.41661380 | 0.21920815 |
| C | 0.43379680 | -6.14970965 | 3.65992890 | C | -6.28212718 | 1.26845717 | 1.33546224 |
| H | 1.35164413 | -6.24520765 | 4.25827983 | H | -7.08915486 | 1.09203875 | 2.06446688 |
| H | -0.42984615 | -6.36357219 | 4.30643346 | H | -5.31457592 | 1.02195503 | 1.80178683 |
| H | 0.45677923 | -6.84173714 | 2.80932277 | H | -6.27541198 | 2.32070156 | 1.02186395 |
| N | -0.46101959 | 3.63212422 | -3.27814703 | N | 4.70469546 | 0.04789100 | -0.12744180 |
| C | -0.22687826 | 3.57272581 | -4.71167068 | C | 5.02668927 | 1.47240195 | -0.16122126 |
| H | 0.80378451 | 3.25419215 | -4.96818725 | H | 4.63476936 | 1.94738009 | -1.07695149 |
| H | -0.93266852 | 2.87397539 | -5.18582513 | H | 4.61089583 | 1.99499354 | 0.71720736 |
| H | -0.40675990 | 4.56790818 | -5.14027524 | H | 6.11826301 | 1.58317939 | -0.14946855 |
| C | -0.10922919 | 4.88325316 | -2.62296998 | C | 5.78537440 | -0.93152360 | -0.09396217 |
| H | -0.25271594 | 5.70440115 | -3.33795466 | H | 6.74729927 | -0.40367870 | -0.09440552 |
| H | -0.77267512 | 5.07778276 | -1.76641987 | H | 5.71571851 | -1.55444034 | 0.81478036 |
| H | 0.93963165 | 4.90407019 | -2.26505302 | H | 5.73612522 | -1.59358128 | -0.97605078 |
| *syn*-**9BgCoum** - ***S*$_1$** | | | | *anti*-**9BgCoum**- ***S*$_1$** | | | |
| C | 0.001657 | 0.111937 | -3.369842 | C | -0.37200127 | -0.45174801 | -3.42441581 |
| C | 0.007903 | 0.028131 | -1.963791 | C | -0.25624238 | -0.45819863 | -2.02274500 |
| C | 0.013022 | -1.225665 | -1.275254 | C | -0.24427470 | -1.68828812 | -1.29004846 |
| C | 0.012212 | 1.266525 | -1.205289 | C | -0.23381055 | 0.81057998 | -1.32202625 |
| C | 0.017104 | 1.180373 | 0.215897 | C | -0.34754243 | 0.77570325 | 0.09435388 |
| C | 0.021862 | -0.069558 | 0.860119 | C | -0.35131355 | -0.44287928 | 0.79192408 |
| C | 0.017827 | -1.322193 | 0.135019 | C | -0.26110662 | -1.72531910 | 0.11976254 |
| C | -0.000901 | 1.360594 | -4.037190 | C | -0.44934394 | 0.75528502 | -4.16386736 |
| C | 0.007889 | 2.500560 | -1.899095 | C | -0.20239843 | 2.02267326 | -2.09106460 |
| H | 0.019821 | 2.068182 | 0.852952 | H | -0.49249203 | 1.67786904 | 0.69362968 |
| O | 0.030292 | -0.038472 | 2.192213 | O | -0.47114847 | -0.36028815 | 2.11725199 |
| C | 0.025309 | -2.512302 | 0.887286 | C | -0.24508226 | -2.88228820 | 0.92781502 |
| C | 0.032826 | -2.501373 | 2.302100 | C | -0.27077932 | -2.80766279 | 2.34635791 |
| C | 0.011276 | -1.263930 | 3.038240 | C | -0.48015784 | -1.56322199 | 3.01722600 |
| H | 0.029105 | -3.473237 | 0.365164 | H | -0.21276221 | -3.87545570 | 0.46511010 |
| C | 0.002702 | 2.557404 | -3.317791 | C | -0.38787848 | 1.98051023 | -3.51492800 |
| H | 0.012097 | -2.149728 | -1.866466 | H | -0.25666685 | -2.63253883 | -1.84977056 |
| H | -0.004366 | 1.385248 | -5.131405 | H | -0.53742054 | 0.71843831 | -5.25441887 |
| O | -0.029151 | -1.014419 | 4.217578 | O | -0.67519594 | -1.26265627 | 4.17567183 |

| | | | | | | | |
|---|---|---|---|---|---|---|---|
| C | 0.062763 | -3.752347 | 3.082040 | C | -0.34090002 | -4.10232202 | 3.07901770 |
| O | 0.286062 | -3.878192 | 4.274948 | O | -0.80254468 | -5.11305138 | 2.57241676 |
| O | -0.211305 | -4.827780 | 2.271764 | O | 0.11992223 | -4.17573015 | 4.36433645 |
| C | -0.132884 | -6.080013 | 2.955754 | C | 1.19274437 | -3.33212582 | 4.78870672 |
| H | 0.872373 | -6.242367 | 3.373518 | H | 1.67154359 | -2.82830349 | 3.93363586 |
| H | -0.861105 | -6.126488 | 3.779149 | H | 0.82774054 | -2.57587676 | 5.49674724 |
| H | -0.360649 | -6.844052 | 2.201504 | H | 1.93104766 | -3.98649308 | 5.27984469 |
| H | 0.000693 | 3.532905 | -3.811471 | H | -0.36009338 | 2.90357448 | -4.09938080 |
| H | -0.002002 | -0.815702 | -3.952446 | N | 0.02800077 | 3.24344897 | -1.49518171 |
| N | 0.010509 | 3.727013 | -1.178309 | C | -0.34807392 | 4.49093434 | -2.15521338 |
| C | -1.234445 | 4.204930 | -0.601621 | H | -1.27590252 | 4.34992148 | -2.72479643 |
| H | -2.066644 | 3.914604 | -1.258176 | H | -0.52163879 | 5.24751784 | -1.37411939 |
| H | -1.416348 | 3.780443 | 0.406863 | H | 0.45414866 | 4.85404636 | -2.82458768 |
| H | -1.198804 | 5.303189 | -0.515679 | C | 0.96463564 | 3.41935649 | -0.38051890 |
| C | 1.260363 | 4.207300 | -0.613920 | H | 1.46075326 | 2.46780133 | -0.15608164 |
| H | 1.453859 | 3.783811 | 0.392747 | H | 1.71642133 | 4.16843555 | -0.68725562 |
| H | 2.086333 | 3.919172 | -1.279192 | H | 0.44635099 | 3.78933566 | 0.52046666 |
| H | 1.222746 | 5.305497 | -0.527918 | H | -0.41653786 | -1.41098381 | -3.95385464 |
| *syn*-**8BgCoum** (ethyl) - ***S*$_1$** | | | | *anti*-**8BgCoum** (ethyl - ***S*$_1$** | | | |
| C | -0.03141868 | 0.53975033 | -2.57405597 | C | -0.71326487 | -0.26250091 | -3.12162637 |
| C | 0.03274147 | 0.58543932 | -1.18466537 | C | -0.57583395 | -0.21268040 | -1.71047804 |
| C | 0.04561431 | -0.61492324 | -0.34502025 | C | -0.54486630 | -1.36967758 | -0.88529895 |
| C | 0.08123358 | 1.86337921 | -0.56540084 | C | -0.46013740 | 1.08089352 | -1.09878609 |
| C | 0.13865447 | 1.90722591 | 0.88599707 | C | -0.32300793 | 1.18172475 | 0.31284828 |
| C | 0.15060257 | 0.73916092 | 1.67099757 | C | -0.30172881 | 0.01942845 | 1.11153904 |
| C | 0.10285108 | -0.57292551 | 1.03174471 | C | -0.40778618 | -1.28154779 | 0.53193154 |
| C | -0.04862117 | 1.72380532 | -3.35539840 | C | -0.74191885 | 0.88668992 | -3.92678682 |
| C | 0.06889381 | 3.03143065 | -1.32352337 | C | -0.48867714 | 2.24233028 | -1.90361768 |
| H | 0.17543626 | 2.86708900 | 1.41362295 | H | -0.23860088 | 2.15423537 | 0.80885270 |
| O | 0.20412827 | 0.86223432 | 2.96258567 | O | -0.18549175 | 0.21209835 | 2.42910988 |
| C | 0.11390178 | -1.78793163 | 1.81464533 | C | -0.36915147 | -2.37908518 | 1.42408941 |
| C | 0.16889926 | -1.91553098 | 3.23476099 | C | -0.18352382 | -2.20073291 | 2.81866287 |
| C | 0.22392084 | -0.90258417 | 4.17080790 | C | -0.16439096 | -0.89681788 | 3.40781284 |
| H | 0.07578386 | -2.73055269 | 1.26165875 | H | -0.47630515 | -3.40104352 | 1.04561790 |
| C | 0.01596705 | 2.99037191 | -2.75970089 | C | -0.63020776 | 2.16451712 | -3.33299629 |
| H | -0.08118476 | -0.42848115 | -3.08528514 | H | -0.80253276 | -1.24276794 | -3.60353099 |
| H | 0.00803790 | -1.58830170 | -0.85231411 | H | -0.63176901 | -2.36145354 | -1.34526935 |
| H | -0.11272208 | 1.61918053 | -4.43966446 | H | -0.85138605 | 0.77293646 | -5.00611164 |
| H | 0.10406500 | 3.98814228 | -0.79721203 | H | -0.39991947 | 3.21205530 | -1.40716013 |
| O | 0.27548526 | -0.40813506 | 5.23451260 | O | -0.14226671 | -0.51746977 | 4.56163820 |
| C | 0.17289554 | -3.25005629 | 3.89809462 | C | -0.25250202 | -3.42810998 | 3.65677325 |
| O | 0.21792373 | -3.44395954 | 5.10261009 | O | -0.88746693 | -4.41225169 | 3.30951112 |
| O | 0.11909382 | -4.25343237 | 2.98159663 | O | 0.40261176 | -3.46643029 | 4.85283325 |
| C | 0.12049141 | -5.57161353 | 3.56070193 | C | 1.60963414 | -2.71269019 | 5.04684103 |
| H | 1.03175297 | -5.70232872 | 4.16617892 | H | 1.92900433 | -2.25740306 | 4.09522592 |
| H | -0.74301477 | -5.67283092 | 4.23760607 | H | 1.40725096 | -1.90429225 | 5.76304434 |
| N | 0.05108218 | 4.16817961 | -3.49870509 | N | -0.65370673 | 3.32346286 | -4.06733036 |
| C | -0.27665319 | 4.07862242 | -4.91217879 | C | -0.80074208 | 3.24140448 | -5.51496880 |
| H | 0.43022811 | 3.41054203 | -5.42716382 | H | 0.03108797 | 2.66761938 | -5.95691842 |
| H | -1.30462996 | 3.70497301 | -5.09441819 | H | -1.74812914 | 2.74296610 | -5.78061415 |
| H | -0.17775577 | 5.07606284 | -5.36139793 | H | -0.79913895 | 4.25462357 | -5.93337781 |
| C | -0.31929980 | 5.40817535 | -2.83270275 | C | -0.53898550 | 4.63139220 | -3.43018562 |
| H | -0.26702743 | 6.22631293 | -3.56346562 | H | -0.58120196 | 5.40289334 | -4.20780895 |
| H | -1.34117652 | 5.38048949 | -2.40411623 | H | -1.36567494 | 4.80217488 | -2.72100015 |
| H | 0.38981210 | 5.64691278 | -2.02533040 | H | 0.41779061 | 4.72849826 | -2.89115979 |
| C | 0.05786155 | -6.55489767 | 2.41028912 | C | 2.66583881 | -3.67237480 | 5.56681064 |
| H | 0.92723509 | -6.43119922 | 1.74724037 | H | 2.87647849 | -4.45898441 | 4.82609009 |
| H | 0.05641105 | -7.58607667 | 2.79688264 | H | 3.60043489 | -3.12868066 | 5.78226686 |
| H | -0.85750024 | -6.40171911 | 1.81918573 | H | 2.32018362 | -4.15494980 | 6.49352722 |
| *syn*-**8BgCoum** (benzyl) - ***S*$_1$** | | | | *anti*-**8BgCoum** (benzyl) - ***S*$_1$** | | | |
| C | -0.35998191 | 1.51663517 | -2.77959588 | C | -0.91091004 | 0.59039464 | -4.12036799 |
| C | -0.21326364 | 1.67831626 | -1.40509626 | C | -0.95564259 | 0.57978969 | -2.70293689 |

| | | | | | | | |
|---|---|---|---|---|---|---|---|
| C | -0.03900777 | 0.55439768 | -0.48236703 | C | -0.83179654 | -0.60390310 | -1.92412032 |
| C | -0.22964188 | 3.00189973 | -0.88821946 | C | -1.13342983 | 1.83636371 | -2.03423546 |
| C | -0.06624246 | 3.16765209 | 0.54575938 | C | -1.18617666 | 1.87606326 | -0.61455253 |
| C | 0.09896628 | 2.07067718 | 1.41273125 | C | -1.06462640 | 0.68737373 | 0.13650106 |
| C | 0.11350131 | 0.71198089 | 0.87872333 | C | -0.88251818 | -0.57746074 | -0.50068418 |
| C | -0.52021376 | 2.62946805 | -3.64462141 | C | -1.03364092 | 1.76474347 | -4.87832143 |
| C | -0.39034398 | 4.10084427 | -1.72863836 | C | -1.25828170 | 3.02404239 | -2.79180373 |
| H | -0.06866157 | 4.16716462 | 0.99524060 | H | -1.32480773 | 2.81869294 | -0.07498331 |
| O | 0.23708553 | 2.30215489 | 2.68285485 | O | -1.13572644 | 0.82139406 | 1.46385252 |
| C | 0.27984060 | -0.43124232 | 1.74730633 | C | -0.77141023 | -1.70572278 | 0.34742845 |
| C | 0.45994987 | -0.43370536 | 3.16344632 | C | -0.79534362 | -1.58269129 | 1.76124960 |
| C | 0.51324806 | 0.65586399 | 4.00953156 | C | -1.03666366 | -0.32240128 | 2.40058807 |
| H | 0.28717318 | -1.41595914 | 1.27142315 | H | -0.65999531 | -2.70832245 | -0.07821352 |
| C | -0.55532219 | 3.93925526 | -3.14770044 | C | -1.21024403 | 3.00798880 | -4.22884208 |
| H | -0.34101830 | 0.51071974 | -3.21437696 | H | -0.77539673 | -0.36138886 | -4.64645211 |
| H | -0.03076015 | -0.45693344 | -0.91057215 | H | -0.69584984 | -1.56810615 | -2.42859642 |
| H | -0.61688863 | 2.43516709 | -4.71400577 | H | -0.99046557 | 1.69750294 | -5.96619881 |
| H | -0.39159921 | 5.09705602 | -1.28020515 | H | -1.39383220 | 3.96451172 | -2.25155000 |
| O | 0.61733536 | 1.23565758 | 5.02531411 | O | -1.17803886 | 0.00068355 | 3.56151772 |
| C | 0.62098715 | -1.70395713 | 3.92293653 | C | -0.76430335 | -2.84637186 | 2.53310705 |
| O | 0.78860908 | -1.80257944 | 5.12798661 | O | -1.05073655 | -3.92853212 | 2.04222592 |
| O | 0.55497026 | -2.77872486 | 3.08758999 | O | -0.43070170 | -2.79692578 | 3.86380051 |
| C | 0.67055079 | -4.04634740 | 3.75961082 | C | 0.70035409 | -2.01588349 | 4.24758478 |
| H | 1.67026235 | -4.14141923 | 4.21382756 | H | 1.03474279 | -1.38621509 | 3.40529121 |
| H | -0.07048115 | -4.08815327 | 4.57529054 | H | 0.39684086 | -1.34555988 | 5.06560569 |
| N | -0.76011791 | 5.04732695 | -3.96243680 | N | -1.33683357 | 4.18805747 | -4.91691224 |
| C | -0.64933829 | 4.84960920 | -5.39843211 | C | -1.28957005 | 4.16711272 | -6.37349688 |
| H | 0.35560416 | 4.49951044 | -5.70984146 | H | -0.32594578 | 3.76106759 | -6.72424501 |
| H | -1.39313038 | 4.11471358 | -5.74182789 | H | -2.10101818 | 3.53957869 | -6.77883938 |
| H | -0.86498245 | 5.80086579 | -5.90342138 | H | -1.40552298 | 5.18992018 | -6.75040594 |
| C | -0.35592690 | 6.35288518 | -3.46195749 | C | -1.52030391 | 5.45836163 | -4.22224744 |
| H | -0.55989983 | 7.10286500 | -4.23781659 | H | -1.59565136 | 6.25675130 | -4.96959898 |
| H | -0.94520037 | 6.63293782 | -2.57538173 | H | -2.44405073 | 5.45286487 | -3.62039857 |
| H | 0.71929187 | 6.40042210 | -3.19692771 | H | -0.66584203 | 5.67679239 | -3.56070191 |
| C | 0.42608986 | -5.11768514 | 2.73178620 | C | 1.83252118 | -2.92464336 | 4.67761496 |
| C | -0.70616648 | -5.04656824 | 1.89654712 | C | 2.88612612 | -2.40011663 | 5.44968849 |
| C | 1.30036936 | -6.21116975 | 2.60930172 | C | 1.87013518 | -4.27687933 | 4.29164097 |
| C | -0.95895118 | -6.05657480 | 0.95768928 | C | 3.97061429 | -3.20842397 | 5.82245058 |
| C | 1.04459682 | -7.22826141 | 1.67477156 | C | 2.95448399 | -5.08607974 | 4.66817571 |
| C | -0.08524968 | -7.15215333 | 0.84604528 | C | 4.00794068 | -4.55713328 | 5.43098821 |
| H | -1.38262031 | -4.19097335 | 1.98388149 | H | 2.85551948 | -1.35052467 | 5.76411272 |
| H | 2.18809195 | -6.26654857 | 3.24820151 | H | 1.04145117 | -4.68849318 | 3.70935842 |
| H | -1.84008889 | -5.99134181 | 0.31202871 | H | 4.78185017 | -2.78821440 | 6.42525847 |
| H | 1.73252612 | -8.07475844 | 1.58916229 | H | 2.97317213 | -6.13804529 | 4.36632972 |
| H | -0.28395066 | -7.94099014 | 0.11445389 | H | 4.84952521 | -5.19201299 | 5.72433428 |

**$^{1}H$ and $^{13}C$ NMR spectra**

$^{1}H$ NMR (500 MHz, $CDCl_3$) of **2a**

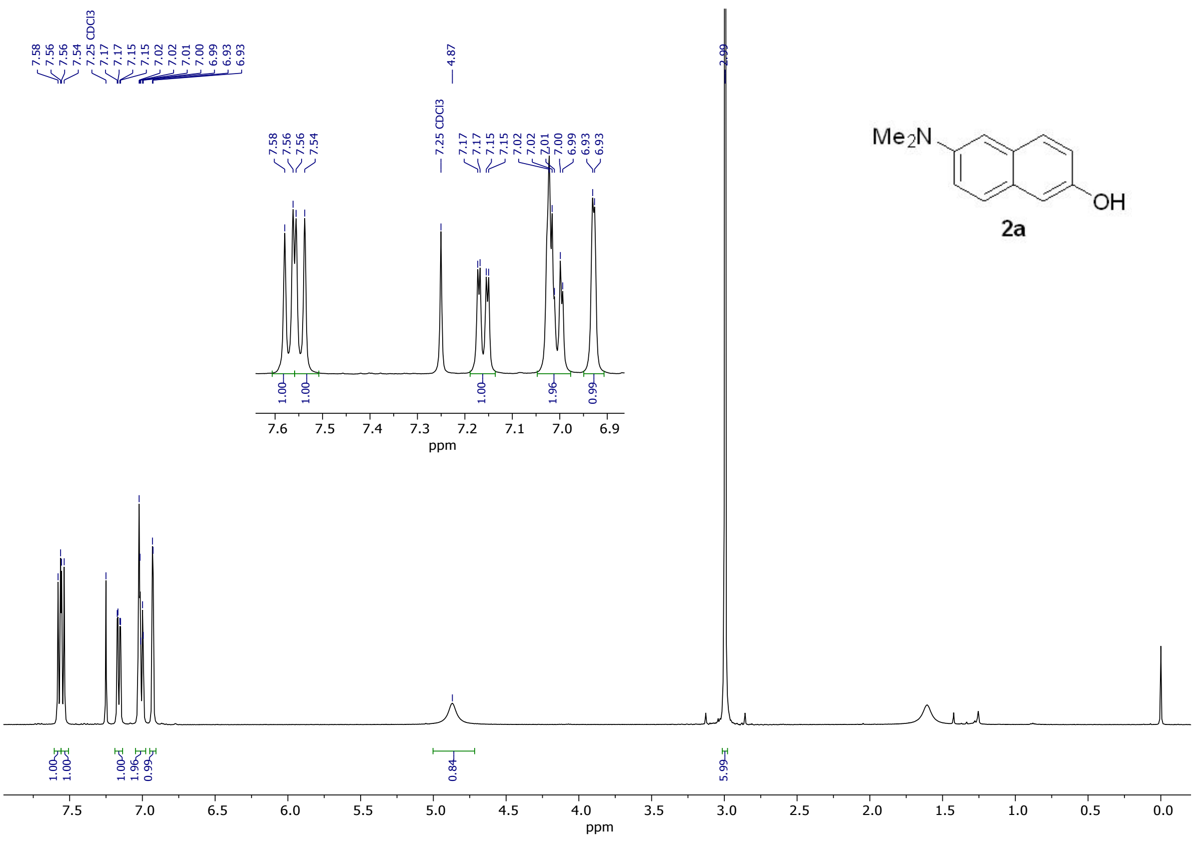

$^{13}C$ NMR (126 MHz, $CDCl_3$) of **2a**

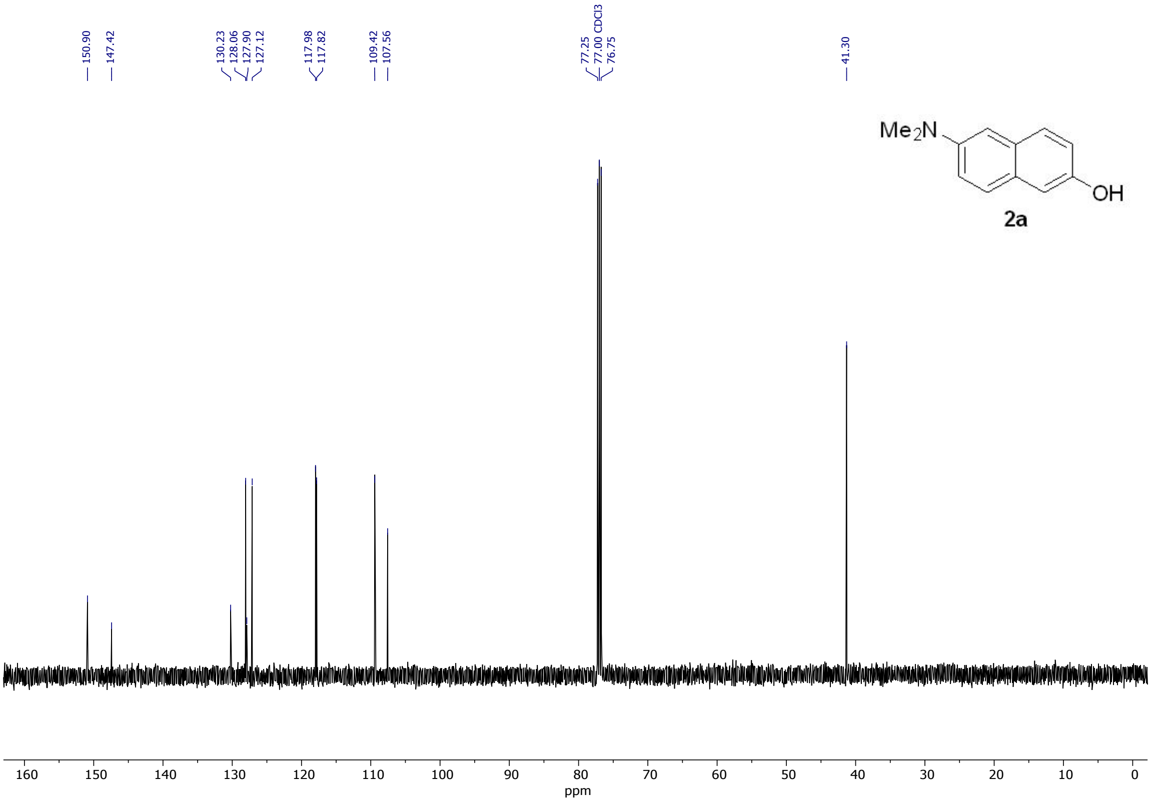

$^{1}H$ NMR (500 MHz, DMSO-$d_6$) of **2b**

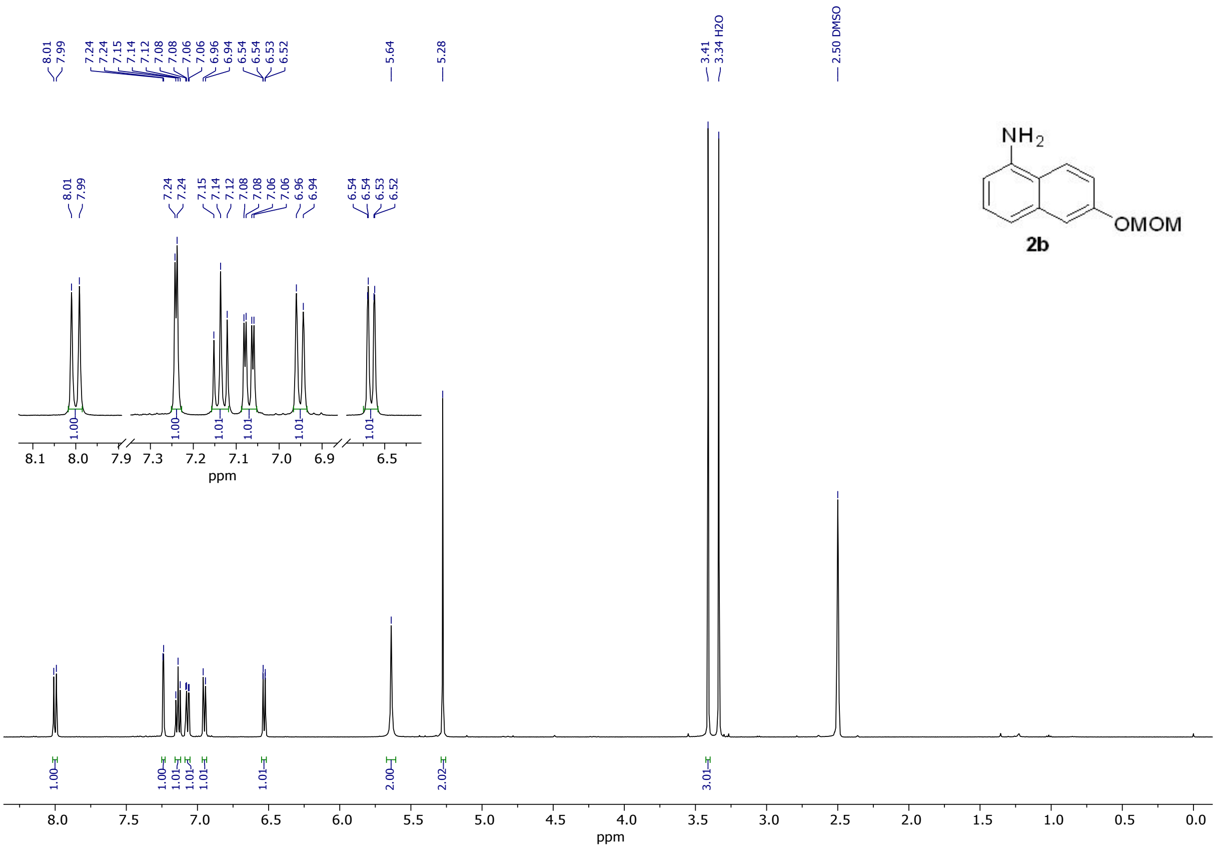


$^{13}C$ NMR (126 MHz, DMSO-$d_6$) of **2b**

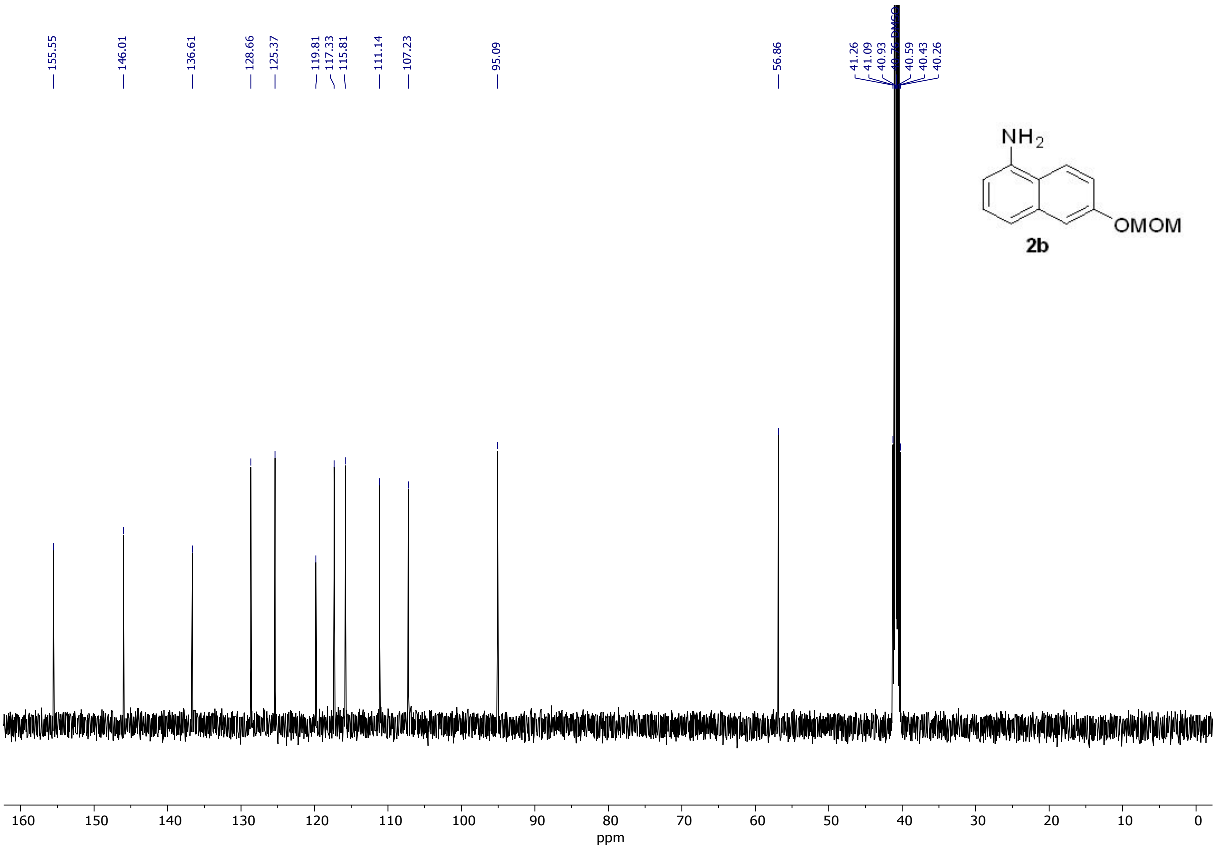

$^{1}H$ NMR (500 MHz, $CDCl_3$) of **2c**

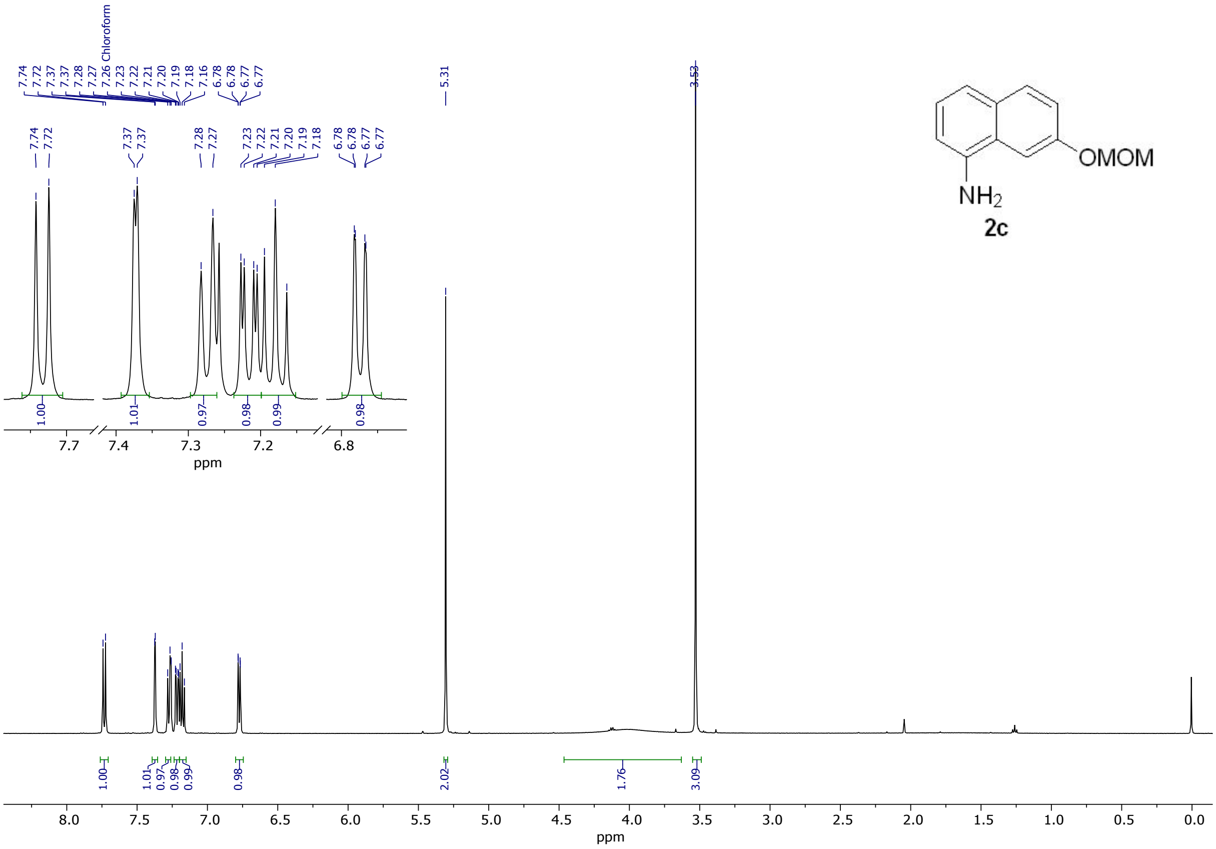


$^{13}C$ NMR (126 MHz, $CDCl_3$) of **2c**

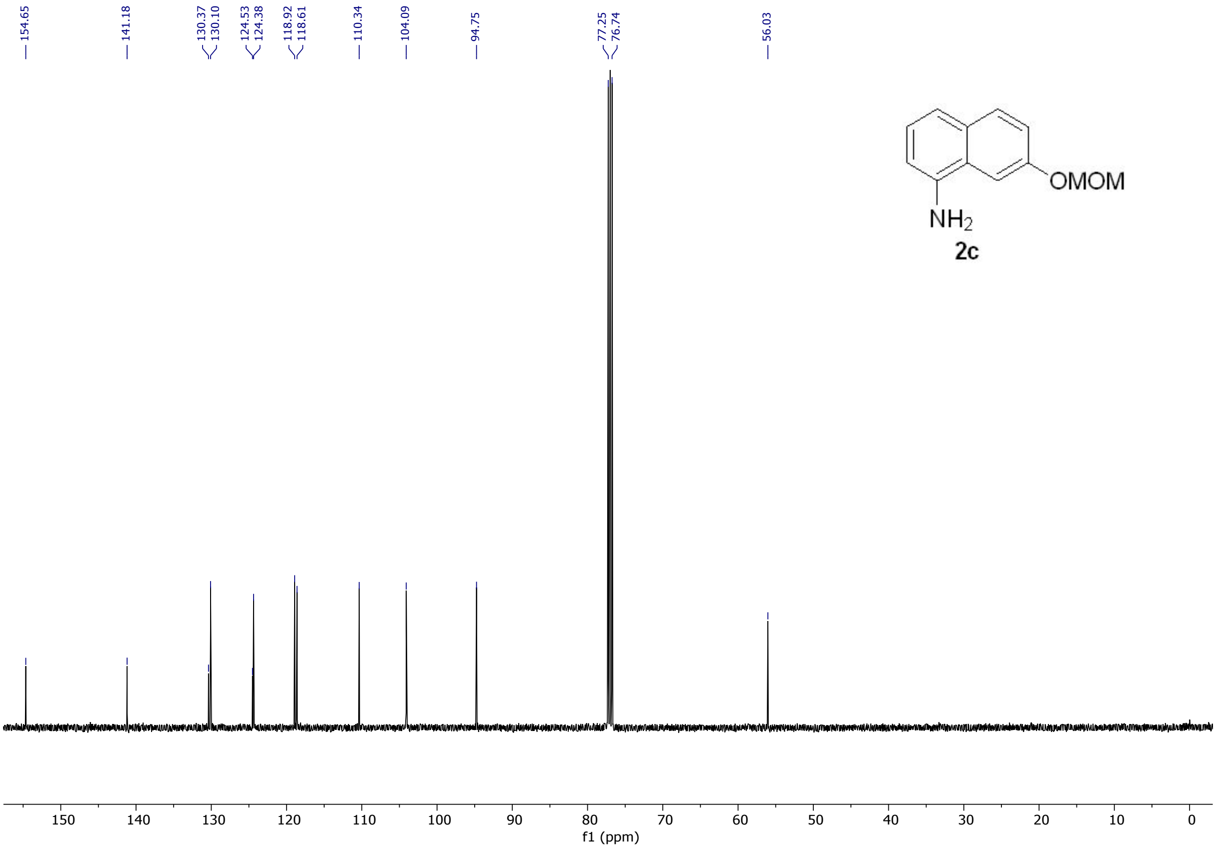

$^{1}H$ NMR (500 MHz, $CDCl_3$) of **3a**

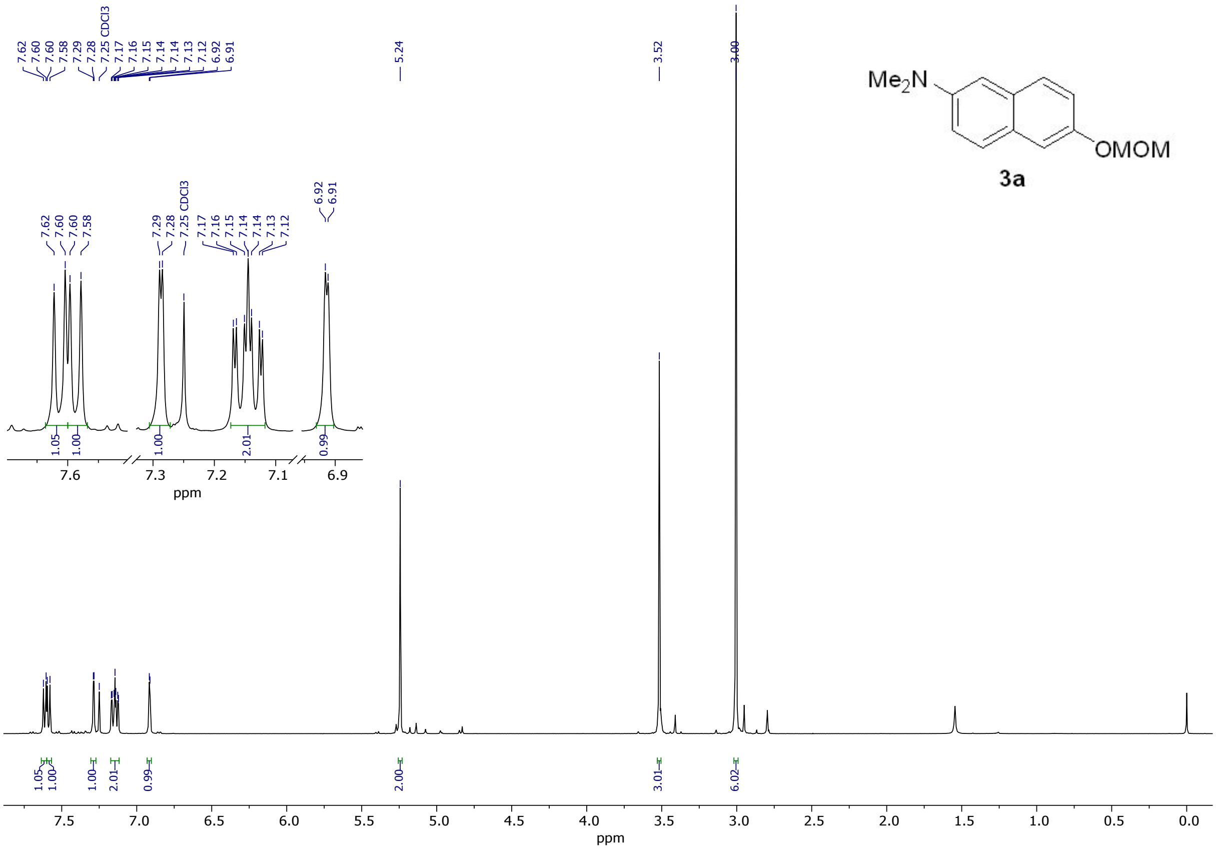


$^{13}C$ NMR (126 MHz, $CDCl_3$) of **3a**

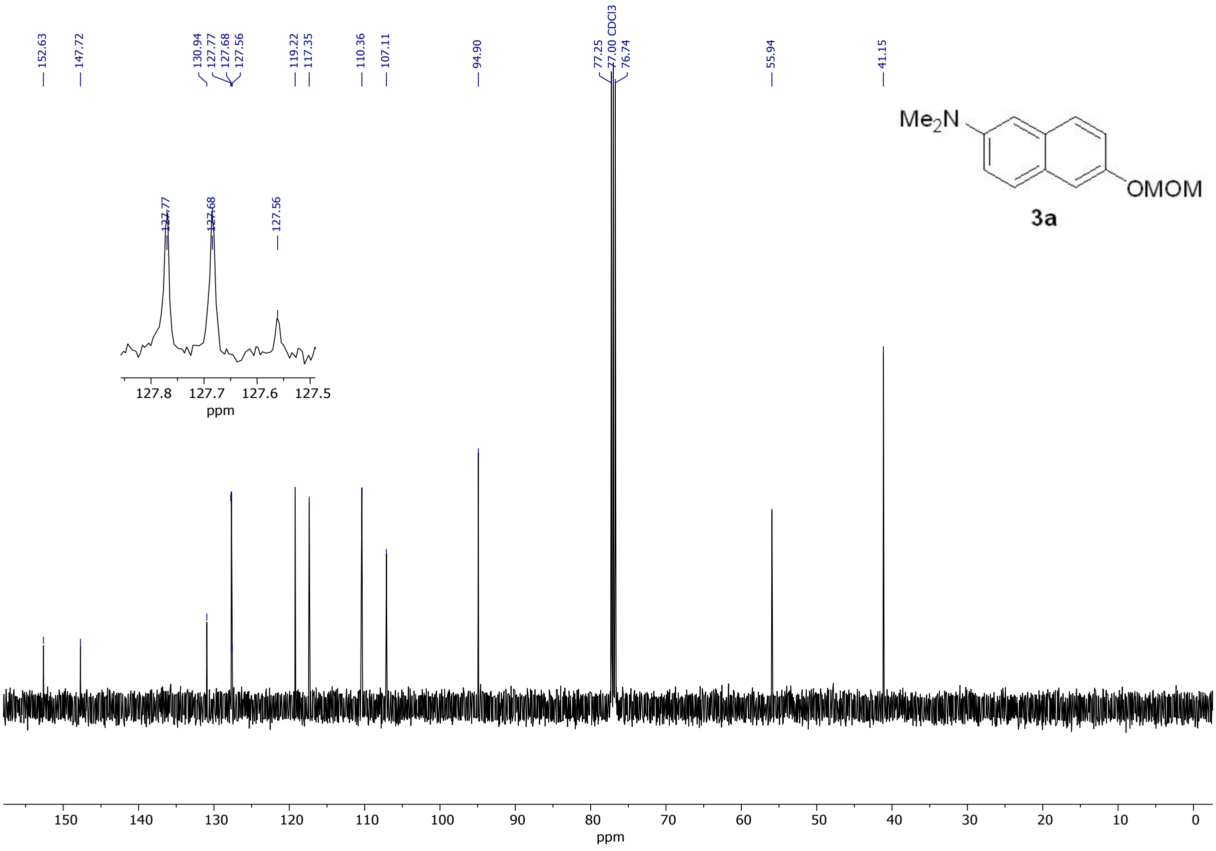

$^{1}H$ NMR (500 MHz, $CDCl_3$) of **3b**

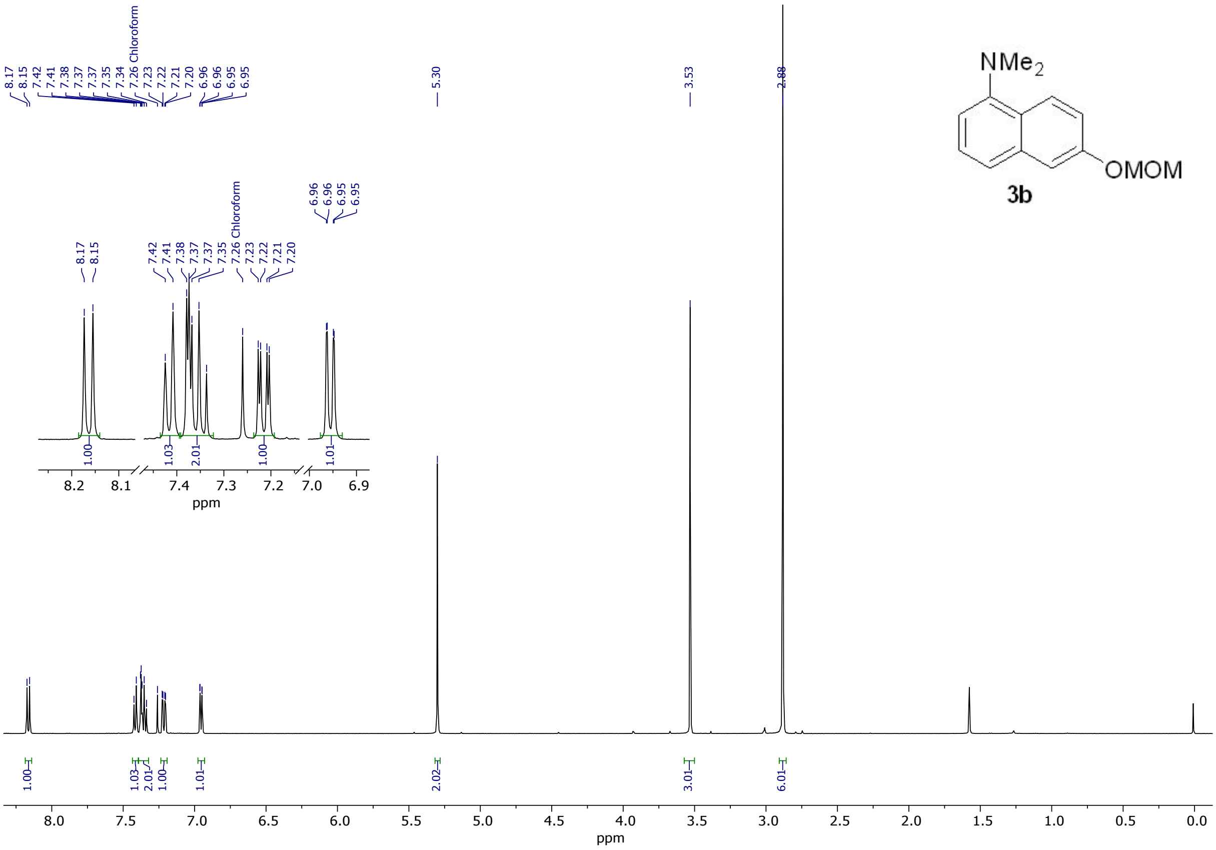


$^{13}C$ NMR (126 MHz, $CDCl_3$) of **3b**

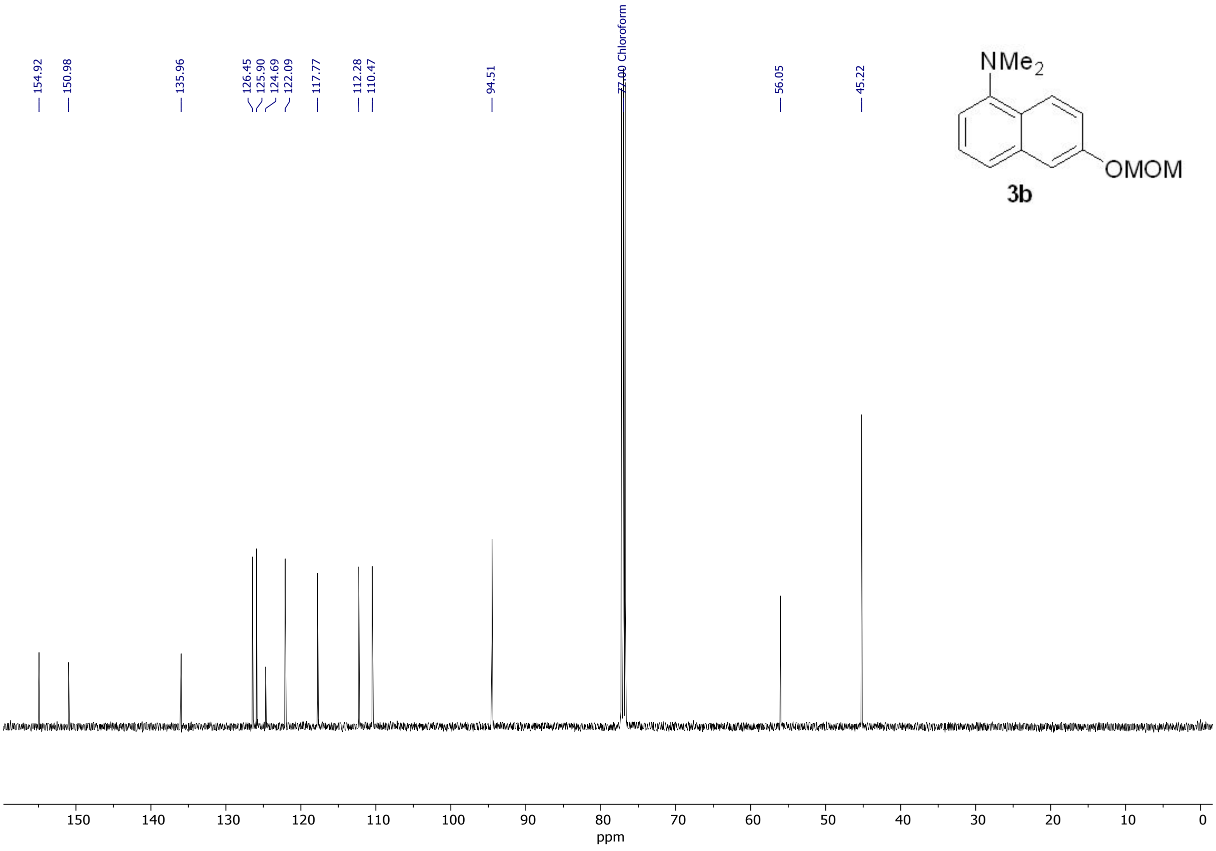

$^{1}H$ NMR (500 MHz, $CDCl_3$) of **3c**

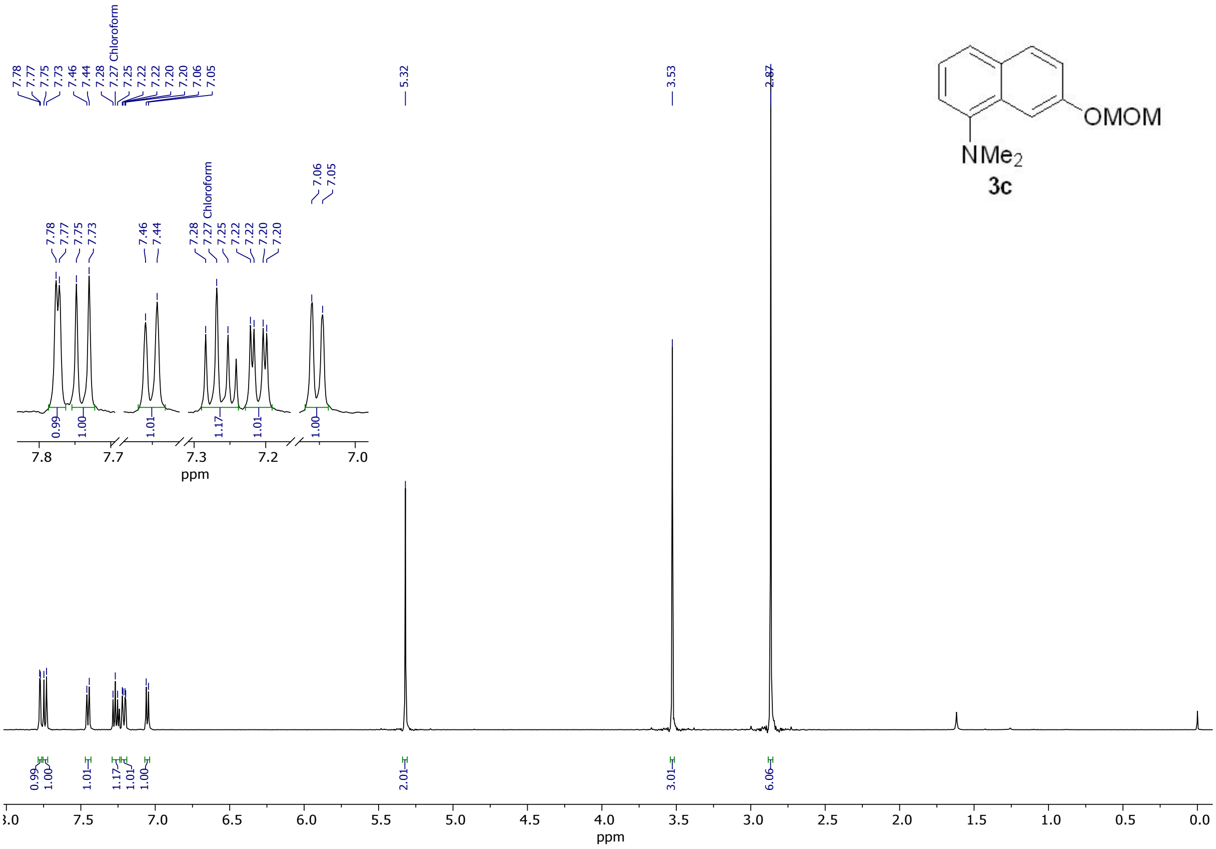


$^{13}C$ NMR (126 MHz, $CDCl_3$) of **3c**

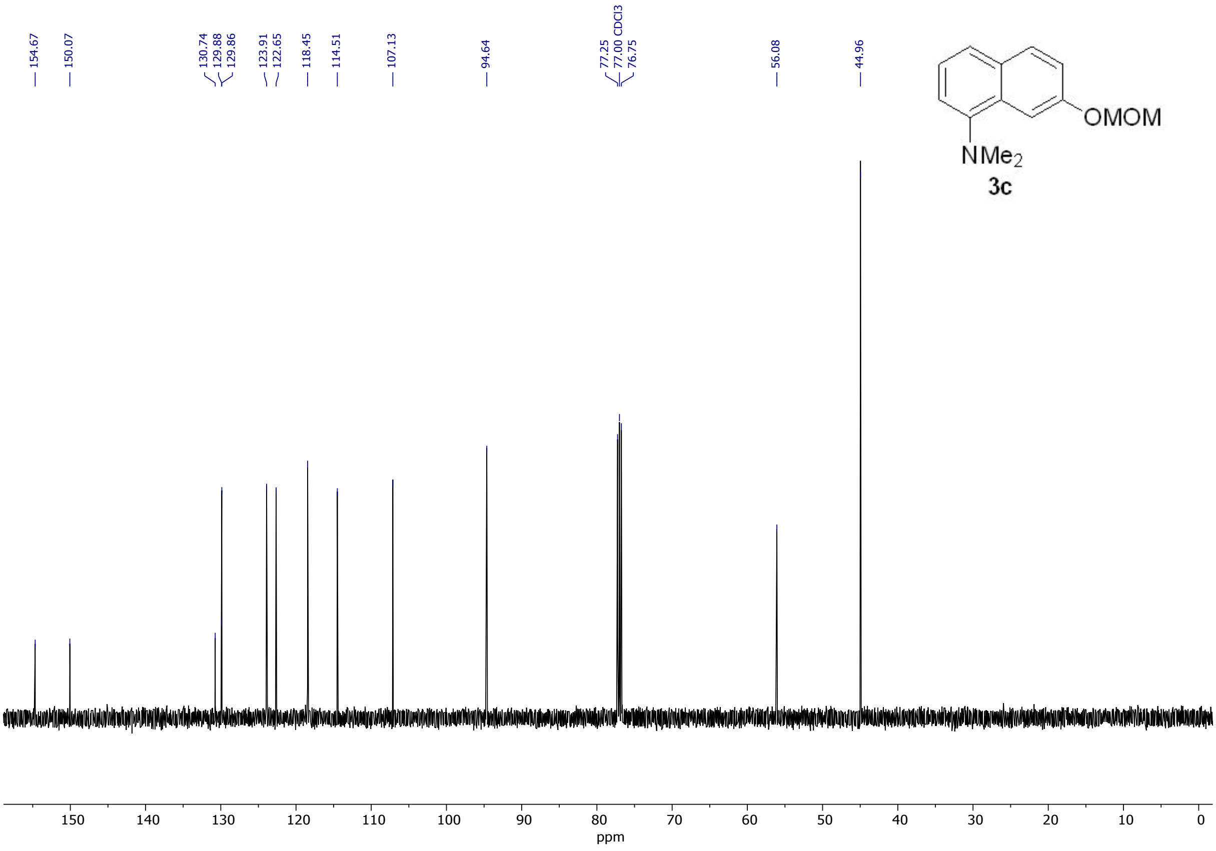

$^{1}H$ NMR (500 MHz, $CDCl_3$) of **4a**

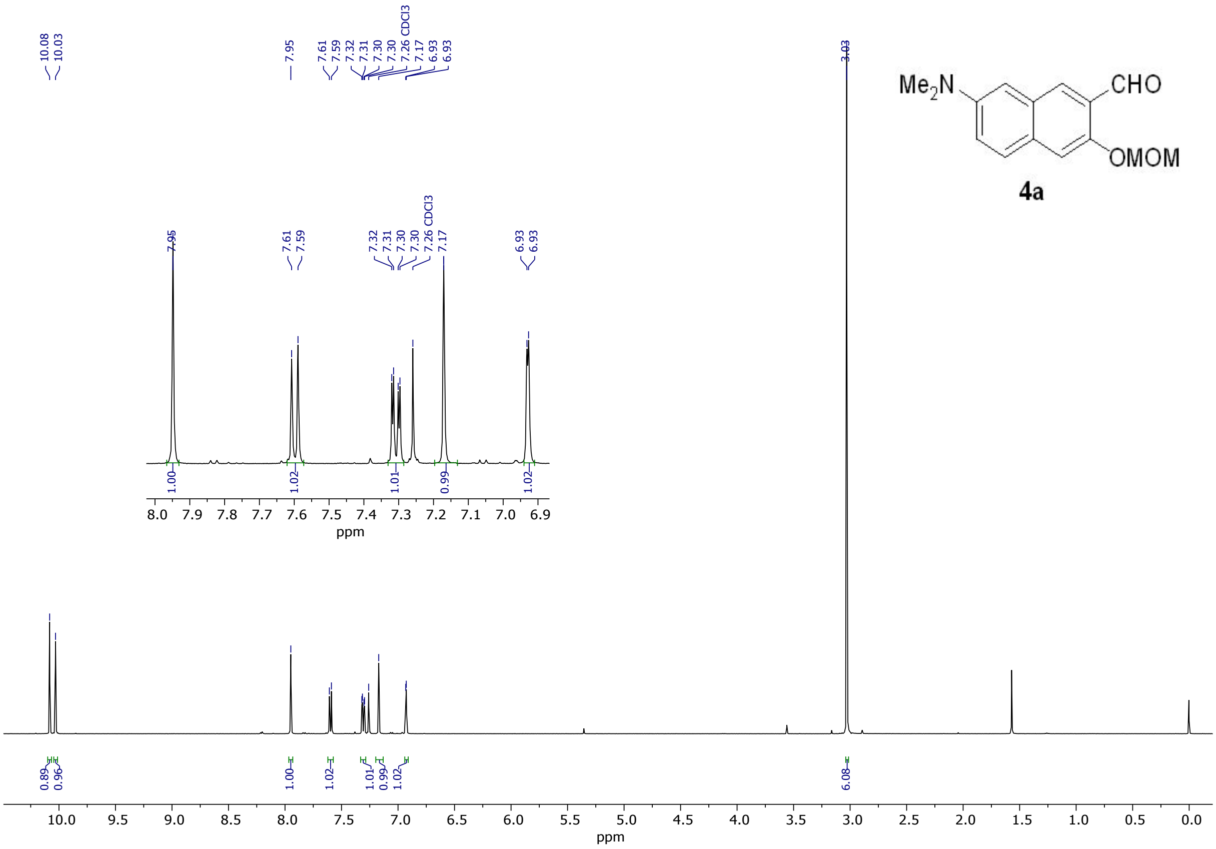


$^{13}C$ NMR (126 MHz, $CDCl_3$) of **4a**

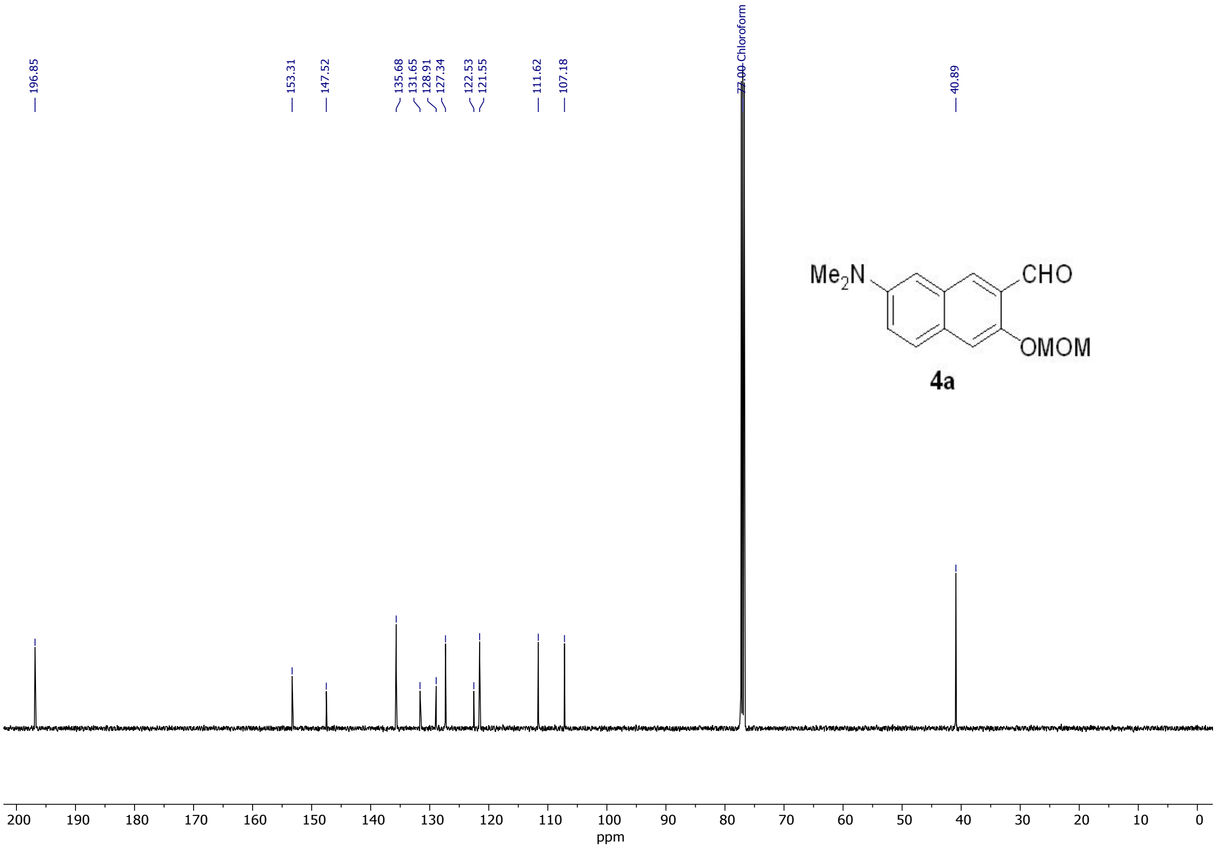

$^{1}$H NMR (500 MHz, DMSO- $d_6$) of **4b**

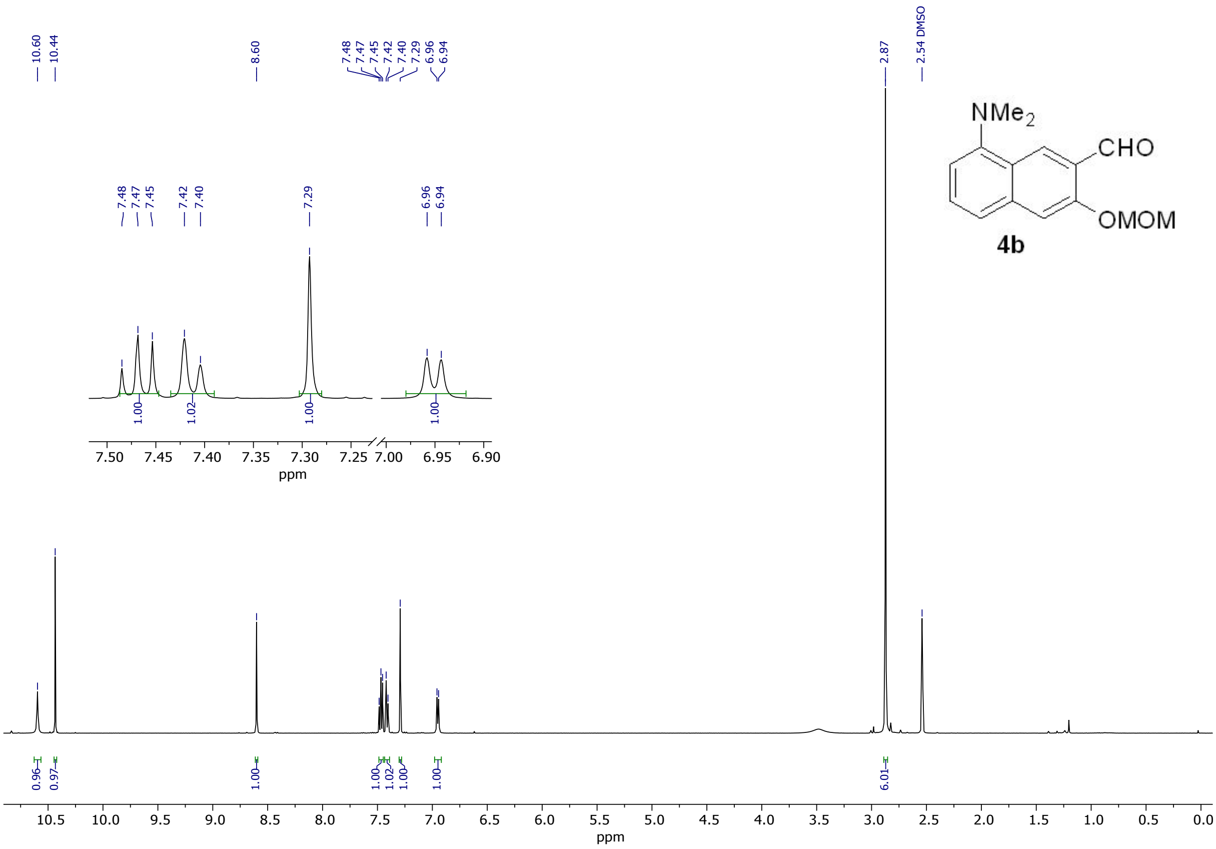


$^{13}$C NMR (126 MHz, DMSO- $d_6$) of **4b**

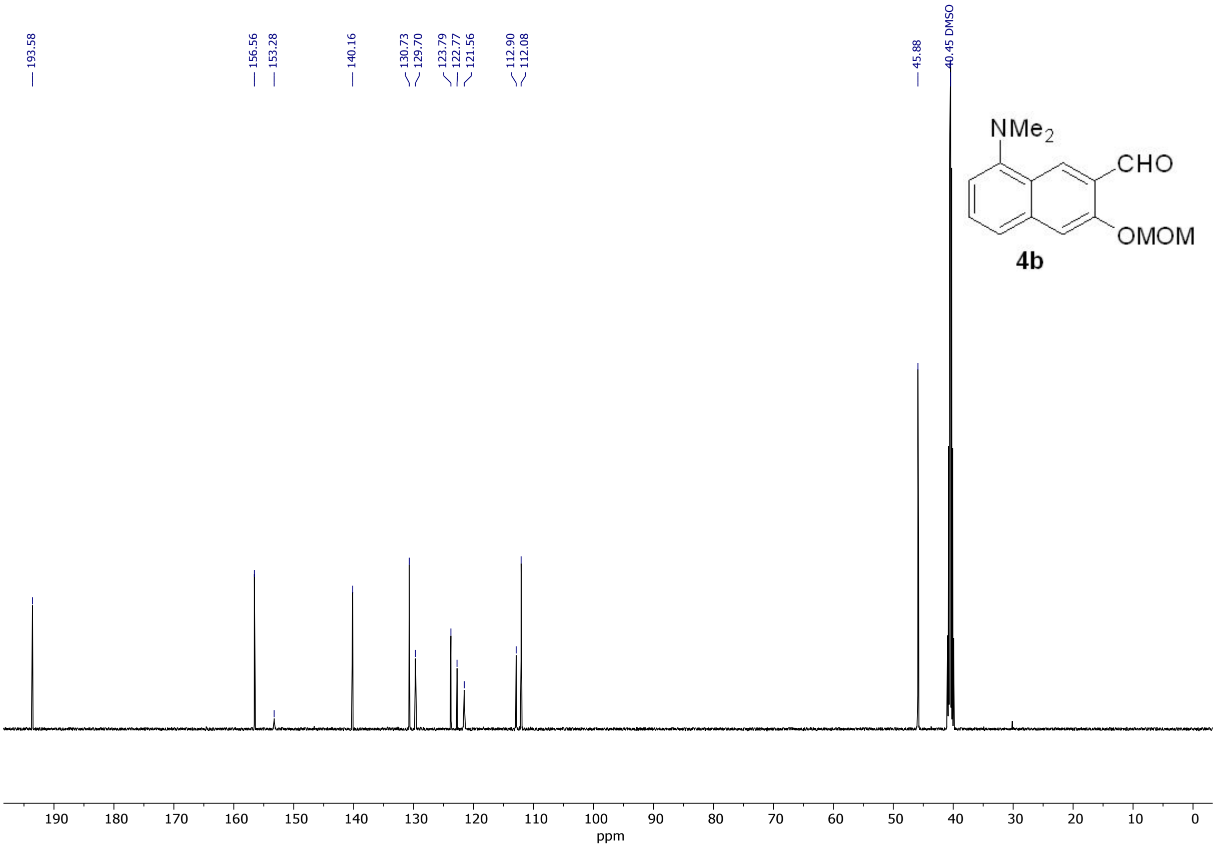

$^{1}$H NMR (500 MHz, $CDCl_3$) of **4c**

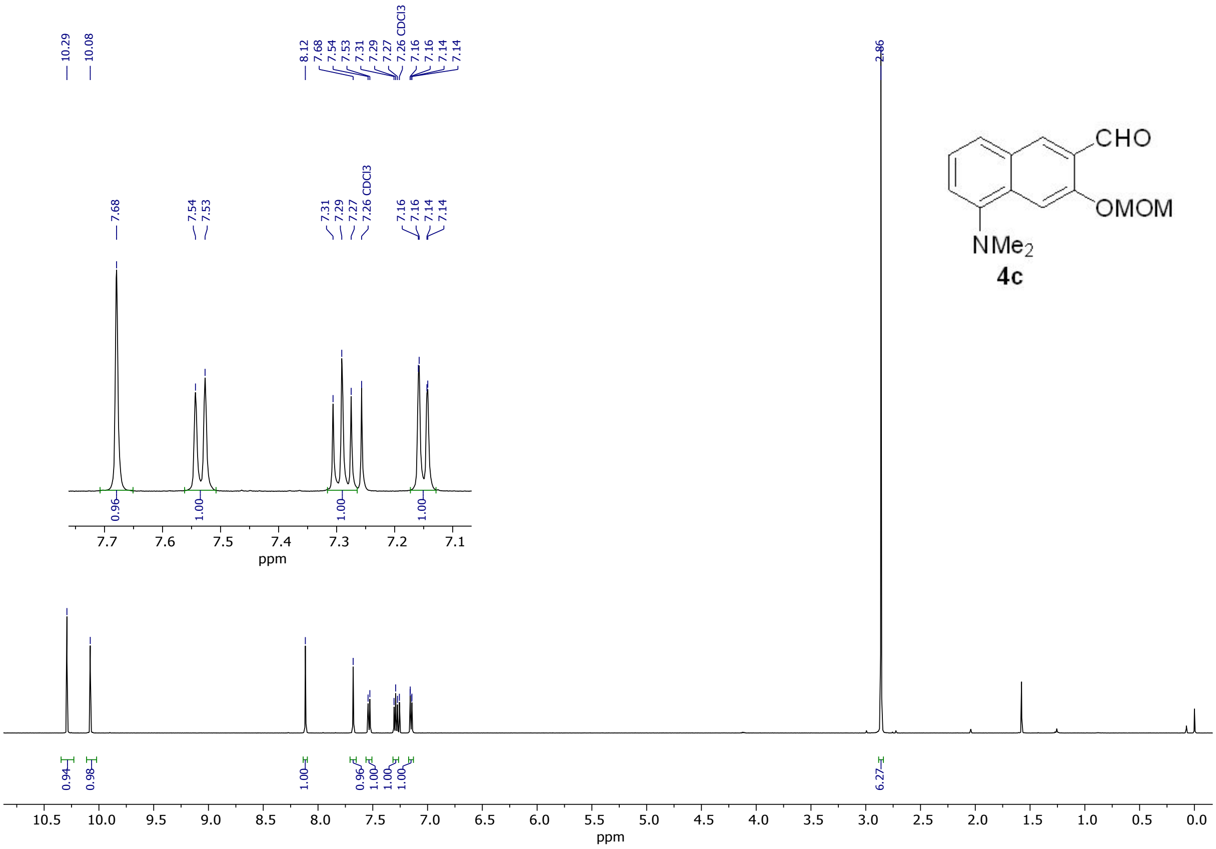


$^{13}$C NMR (126 MHz, $CDCl_3$) of **4c**

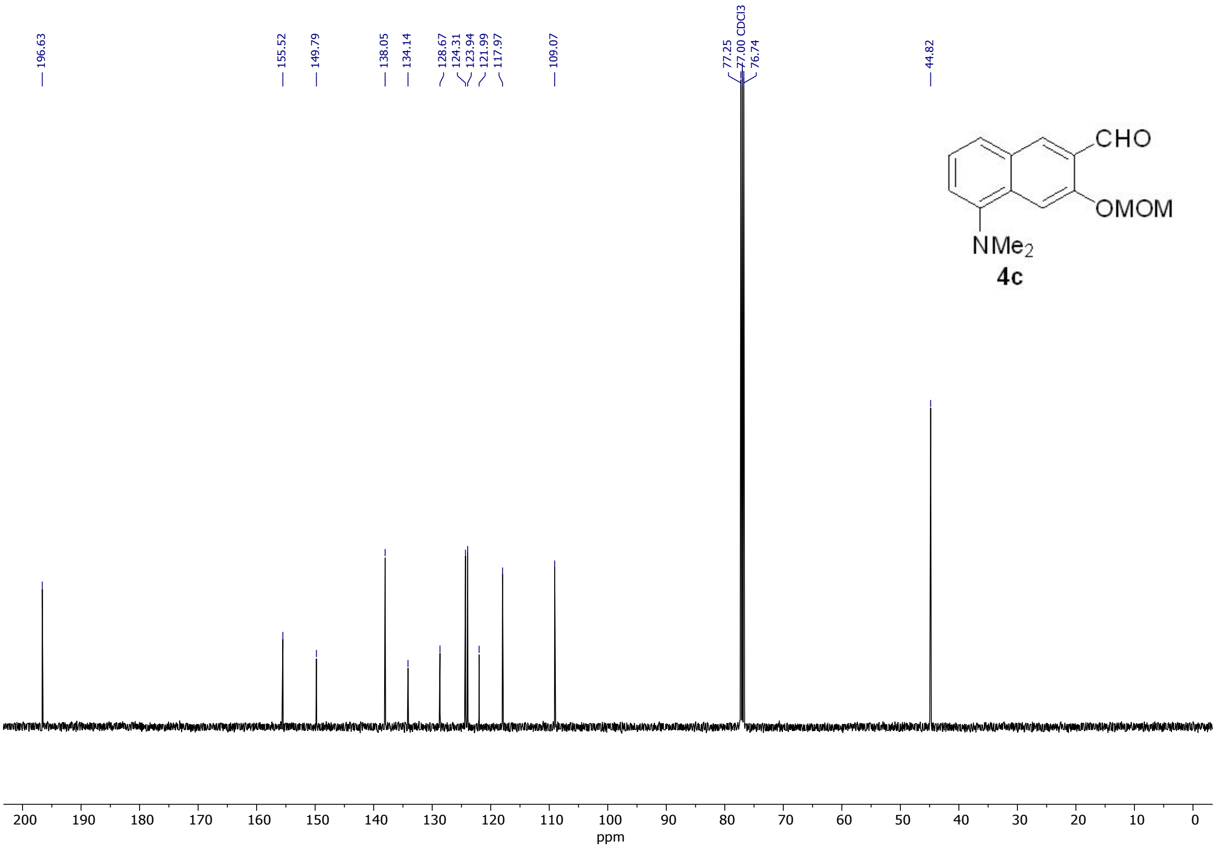

$^{1}H$ NMR (500 MHz, $CDCl_3$) of **7-BgCoum**

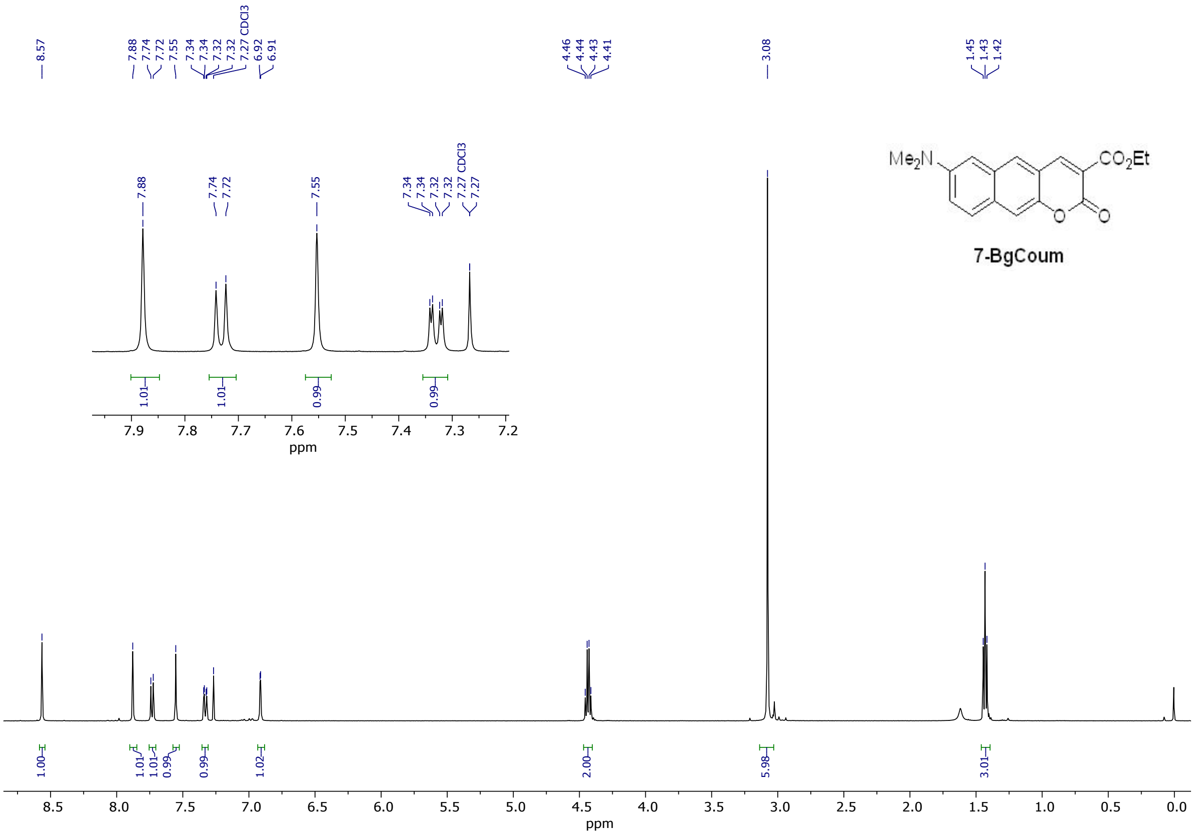


$^{13}C$ NMR (126 MHz, $CDCl_3$) of **7-BgCoum**

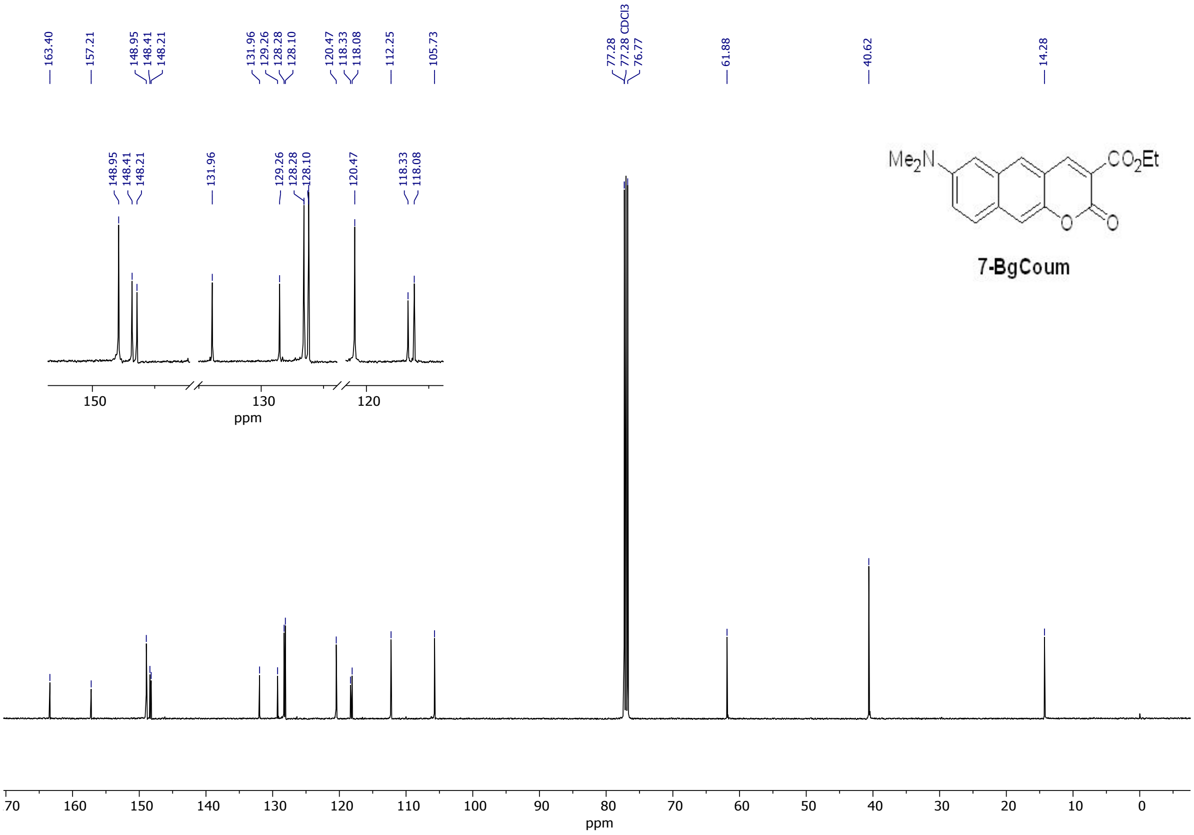

$^{1}$H NMR (500 MHz, $CDCl_3$) of **6-BgCoum**

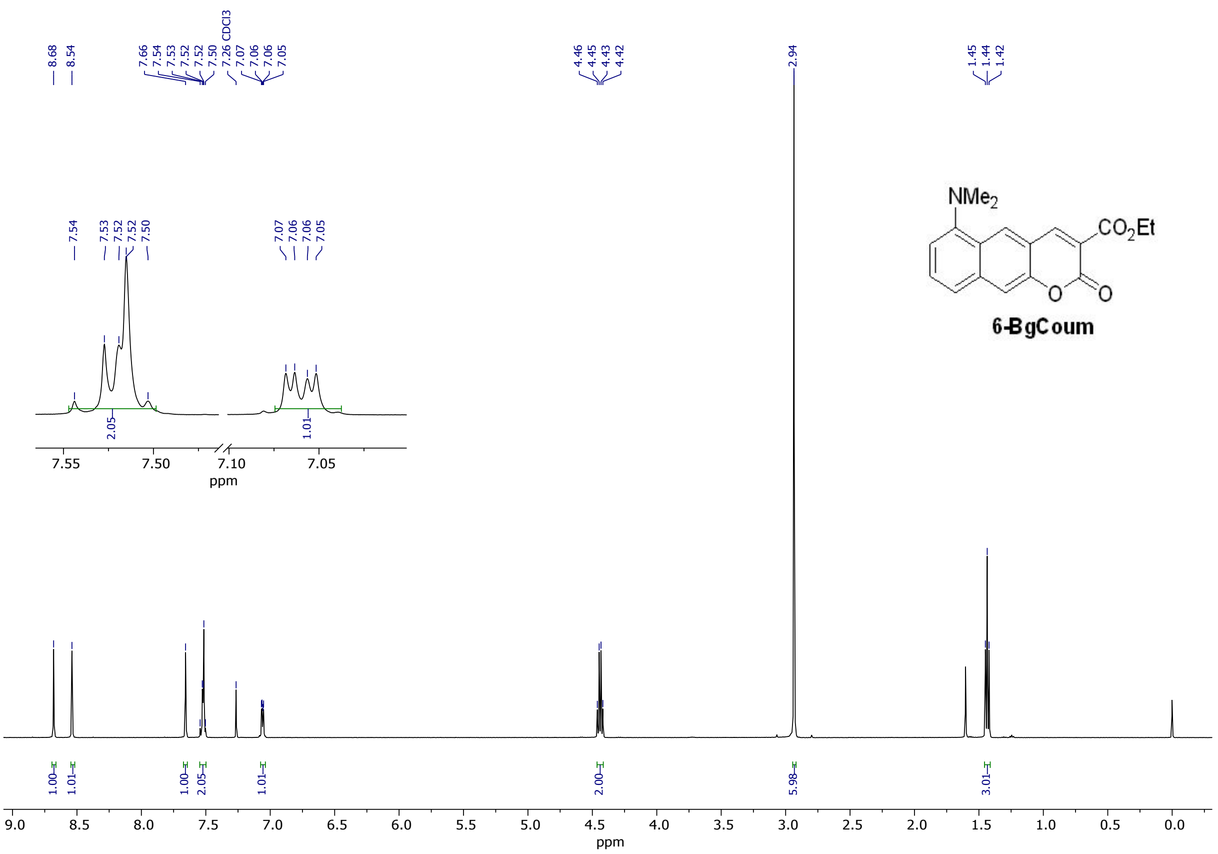


$^{13}$C NMR (126 MHz, $CDCl_3$) of **6-BgCoum**

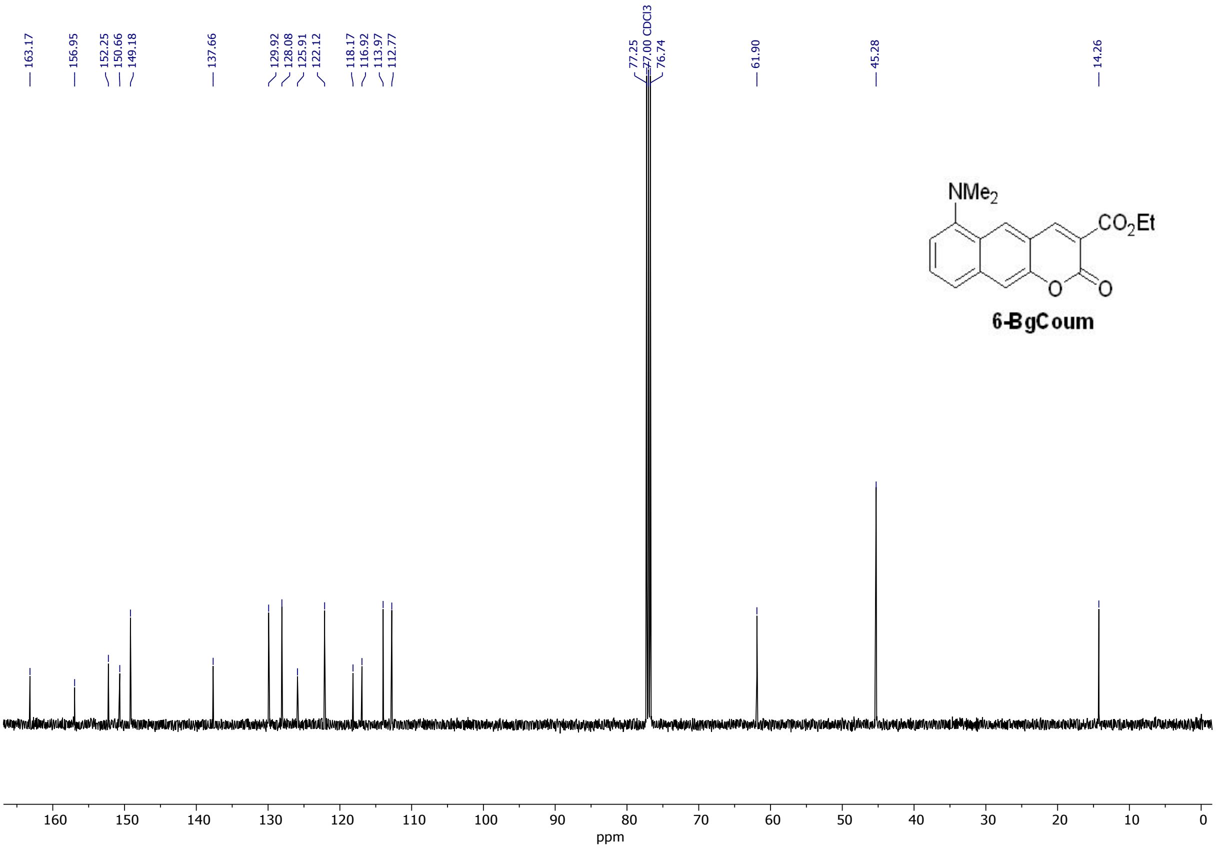

$^{1}$H NMR (500 MHz, $CDCl_3$) of **9-BgCoum**

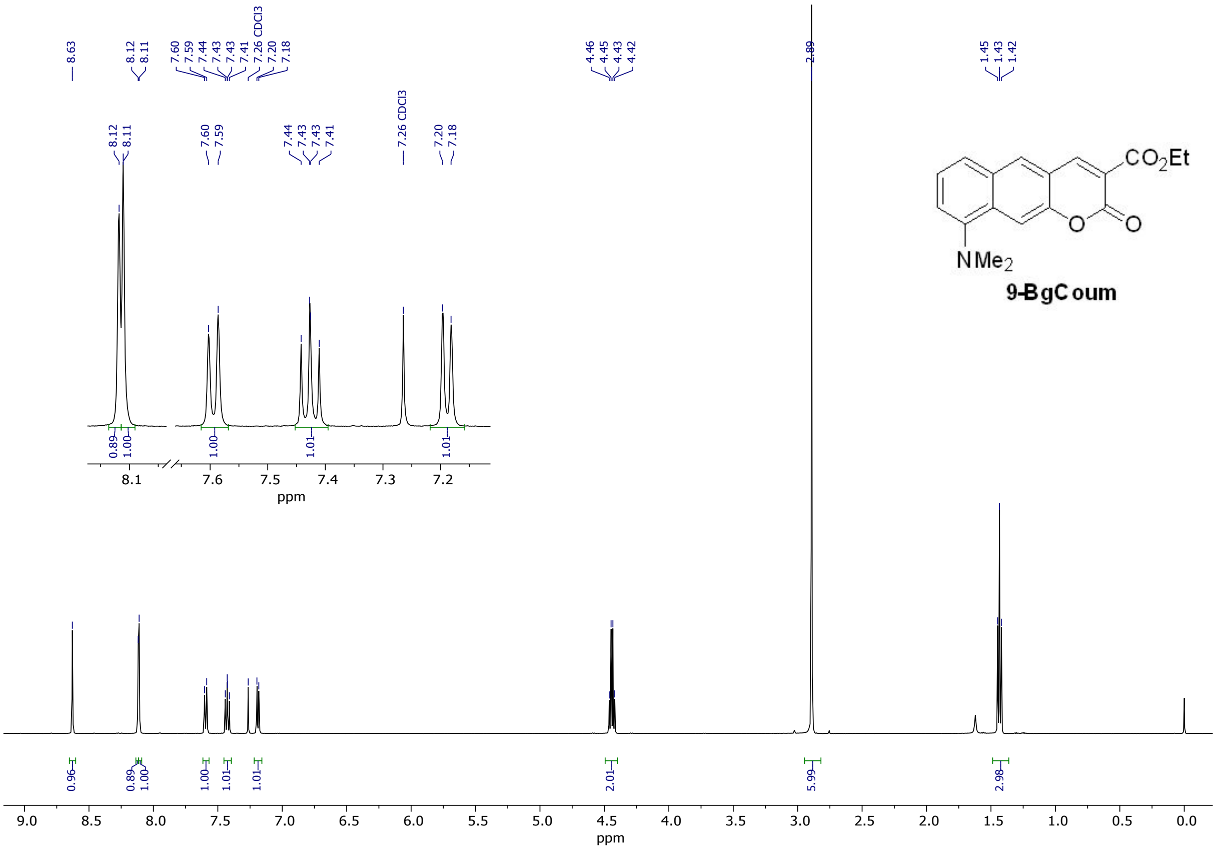


$^{13}$C NMR (126 MHz, $CDCl_3$) of **9-BgCoum**

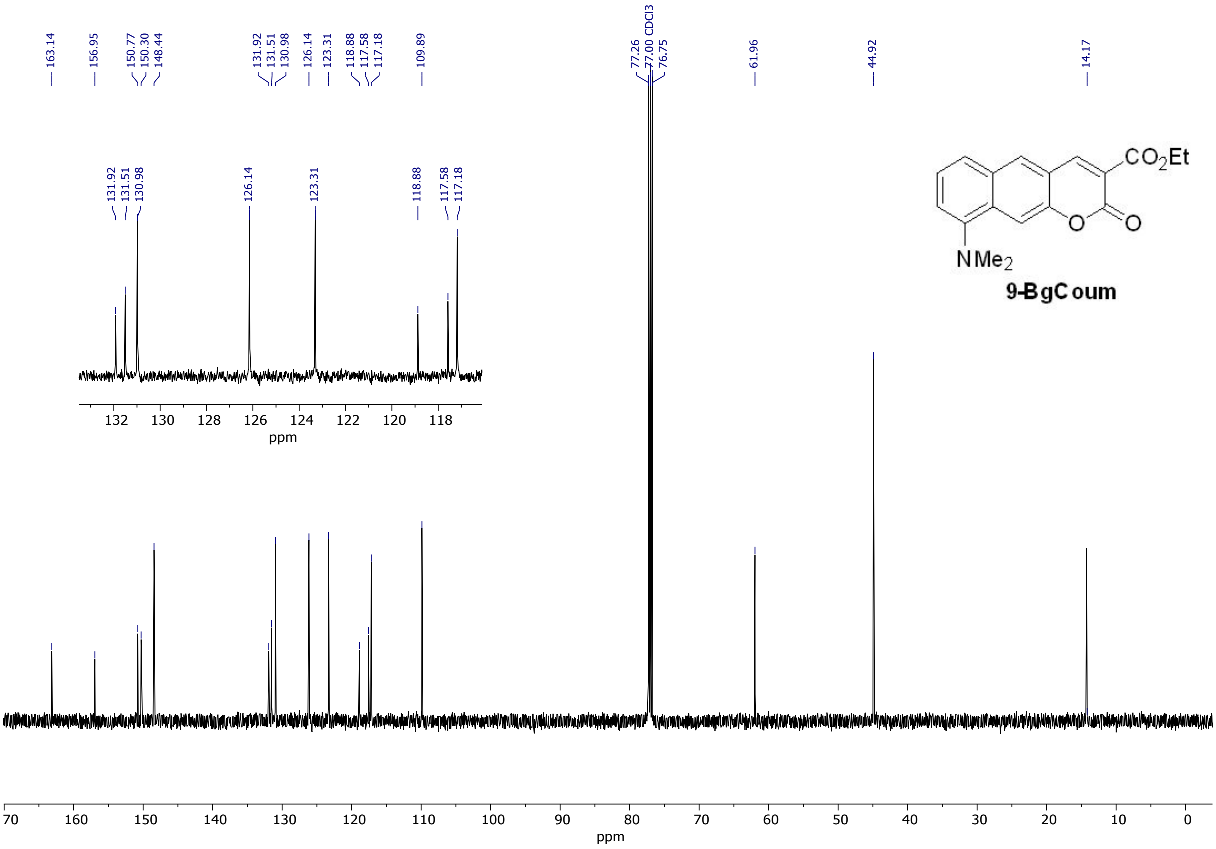

## References


(1) Rathore, K.; Lim, C. S.; Lee, Y.; Park, H. J.; Cho, B. R. A Two-Photon Probe for near-Membrane Zinc Ions. *Asian J. Org. Chem.* **2014**, *3* (10), 1070–1073. https://doi.org/10.1002/ajoc.201402112.

(2) Purc, A.; Espinoza, E. M.; Nazir, R.; Romero, J. J.; Skonieczny, K.; Jezewski, A.; Larsen, J. M.; Gryko, D. T.; Vullev, V. I. Gating That Suppresses Charge Recombination-The Role of Mono-N-Arylated Diketopyrrolopyrrole. *J. Am. Chem. Soc.* **2016**, *138* (39), 12826–12832. https://doi.org/10.1021/jacs.6b04974.

(3) Espinoza, E. M.; Larsen, J. M.; Vullev, V. I. What Makes Oxidized N-Acylanthranilamides Stable? *J. Phys. Chem. Lett.* **2016**, *7* (5), 758–764. https://doi.org/10.1021/acs.jpclett.5b02881.

(4) O'Mari, O.; Vullev, V. I. Electrochemical Analysis in Charge-Transfer Science: The Devil in the Details. *Curr. Opin. Electrochem.* **2022**, *31*, 100862. https://doi.org/10.1016/j.coelec.2021.100862.

(5) Bao, D.; Millare, B.; Xia, W.; Steyer, B. G.; Gerasimenko, A. A.; Ferreira, A.; Contreras, A.; Vullev, V. I. Electrochemical Oxidation of Ferrocene: A Strong Dependence on the Concentration of the Supporting Electrolyte for Nonpolar Solvents. *J. Phys. Chem. A* **2009**, *113* (7), 1259–1267. https://doi.org/10.1021/jp809105f.

(6) Bao, D.; Ramu, S.; Contreras, A.; Upadhyayula, S.; Vasquez, J. M.; Beran, G.; Vullev, V. I. Electrochemical Reduction of Quinones: Interfacing Experiment and Theory for Defining Effective Radii of Redox Moieties. *J. Phys. Chem. B* **2010**, *114* (45), 14467–14479. https://doi.org/10.1021/jp101730e.

(7) Espinoza, E. M.; Xia, B.; Darabedian, N.; Larsen, J. M.; Nuñez, V.; Bao, D.; Mac, J. T.; Botero, F.; Wurch, M.; Zhou, F.; Vullev, V. I. Nitropyrene Photoprobes: Making Them, and What Are They Good For? *European J. Org. Chem.* **2016**, *2016* (2), 343–356. https://doi.org/10.1002/ejoc.201501339.

(8) Laborda, E.; Henstridge, M. C.; Batchelor-Mc Auley, C.; Compton, R. G. Asymmetric Marcus–Hush Theory for Voltammetry. *Chem. Soc. Rev.* **2013**, *42* (12), 4894–4905. https://doi.org/10.1039/c3cs35487c.
